# An optimized procedure for direct access to 1*H*-indazole-3-carboxaldehyde derivatives by nitrosation of indoles†

**DOI:** 10.1039/c8ra01546e

**Published:** 2018-04-09

**Authors:** Arnaud Chevalier, Abdelaaziz Ouahrouch, Alexandre Arnaud, Thibault Gallavardin, Xavier Franck

**Affiliations:** Normandie Univ, CNRS, UNIROUEN, INSA Rouen, COBRA (UMR 6014 and FR 3038) 76000 Rouen France xavier.franck@insa-rouen.fr thibault.gallavardin@univ-rouen.fr

## Abstract

Indazole derivatives are currently drawing more and more attention in medicinal chemistry as kinase inhibitors. 1*H*-indazole-3-carboxaldehydes are key intermediates to access to a variety of polyfunctionalized 3-substituted indazoles. We report here a general access to this motif, based on the nitrosation of indoles in a slightly acidic environment. These very mild conditions allow the conversion of both electron-rich and electron-deficient indoles into 1*H*-indazole-3-carboxaldehydes.

## Abstract 2

The nitrosation of indoles under slightly acidic conditions and reverse addition conditions leads to the preparation of the corresponding indazole-3-carboxaldehydes in high yields and greatly minimizes side reactions.

## Graphical abstract

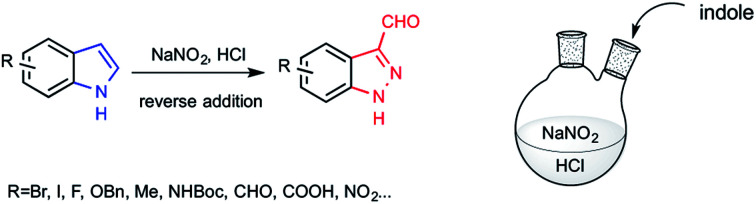

## Introduction

**Fig. 1:**
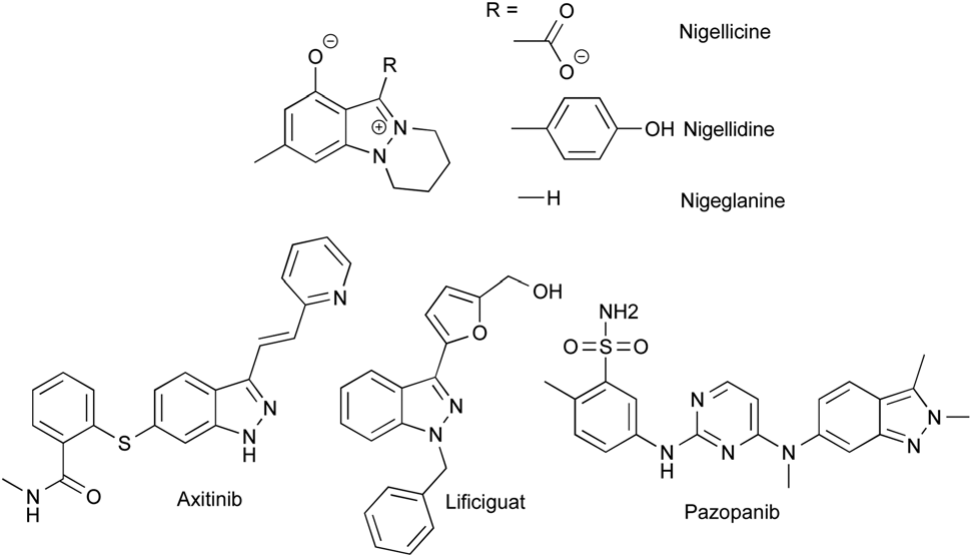
Structures of indazole containing natural compounds from *Nigella*, and kinase inhibitor drugs based on indazole scaffold.

Despite the fact that only a few indazole-containing natural compounds have been isolated to date, all from *Nigella* species,^1–3^ this structural motif is more and more prized for the development of bioactive compounds, particularly, for the design of tyrosine kinase and threonine kinase inhibitors. Indeed, indazoles can be considered as bioisosteres of indoles bearing two successive nitrogen atoms able to promote strong donor and acceptor hydrogen bonding within the hydrophobic pockets of proteins.^4^ The functionalization of indazoles in position 3 led to the discovery of several marketed drugs such as axitinib (Inlyta®), pazopanib (Votrient®) or lificiguat, and more indazole-based drugs are currently under development (Fig. 1).^5–19^

To synthetize 3-substituted indazoles, valuable intermediates are 1*H*-indazole-3-carboxaldehyde derivatives. Indeed, the aldehyde function can be further converted into alkenes through Knoevenagel^9,20^ and Wittig condensations,^14,15^ or into heteroaromatic compounds (*e.g.* oxazoles, thiazoles, benzimidazoles,^12–19^ or isoindazoles) *via* cyclisation reactions; it also provides convenient access to secondary alcohol and amine. Unfortunately, and in contrast to the reactivity of indoles, the direct Vilsmeier–Haack formylation at the C3 position of indazoles is ineffective. Thus alternative methods needed to be implemented to prepare these compounds. Direct metalation in position 3 by deprotonation or halogen–metal exchange was reported, but often leads to the opening of the five-membered heterocyclic ring.^21^ To circumvent this, several protocols using zincation,^22,23^ lithiation,^21,24^ or magnesiation,^25^ have been described *via* dianion formation or using suitable protecting groups. Alternative strategies to introduce the aldehyde function rely on palladium or copper catalyzed cyanation of 3-halogenoindazole followed by reduction with RANEY® nickel or DIBAL,^14,26^ or on a Heck coupling ozonolysis sequence.^9^ Ester functionalized indazoles in position 3 can be accessed using alternative strategies such as aryne cycloaddition,^27–29^ or nitrosation of *ortho*-toluidine derivatives;^30–32^ they can also be converted into the corresponding aldehyde through a reduction-reoxidation sequence.

**Scheme 1:**
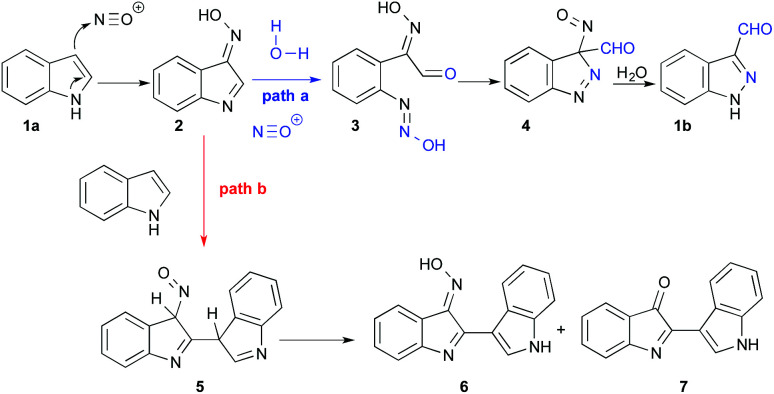
Pathways for nitrosation of 1a and side reaction forming dimers.

The present work explores a different approach to 3-functionalized indazoles, based on the nitrosation of indoles, as first described by Büchi in 1986.^33^ This reaction proceeds through a multistep pathway (Scheme 1, path a) starting with nitrosation of the C3 position of indole 1a, leading to oxime 2. This oxime promotes the addition of water at position 2, which then triggers the opening of the ring; the reaction is terminated by ring-closure to provide the *1H*-indazole-3-carboxaldehyde 1b. Typically, this reaction is carried out by slow addition of acid to a mixture of indole and sodium nitrite, providing good yields with electron deficient indoles.^6^ However, the reported yields are often disappointing when electron rich indoles are used (typically 10–60%) and the precise reaction conditions are generally omitted.^34,35^ The main explanation for these moderate yields is a documented side reaction leading to the formation of deep red-coloured dimers 7 and 6 originating from nucleophilic addition of indole to the intermediate 2 (Scheme 1, path b).^36,37^

The synthetic utility of indole to indazole transformation, as well as the complexity of the reaction process, prompted us to re-evaluate the reaction conditions in order to manipulate the different reactions pathways. The present article provides an optimized procedure suitable for either electron-rich or electron-poor indoles, which greatly minimizes side reactions and delivers significant improvements in the yields.

## Results and discussion

The reaction conditions were first investigated using indole 1a and 5-bromoindole (11a) as starting materials (Table 1). To avoid side reactions such as dimer formation, we envisaged working under low indole concentration. For that purpose, a reverse addition of indole to the nitrosating mixture (composed of NaNO_2_ and HCl in various proportions), was used. During the optimization process, yields were evaluated by ^1^H NMR using piperonal as internal standard. All the reactions were carried under argon to avoid the formation of nitrogen oxide species such as NO_2_˙ in the presence of oxygen.

**Table 1:** Optimisation of the reaction. Addition of indole (1 equiv.) to the nitrosating mixture containing NaNO_2_, HCl in water : DMF 5.3 : 3

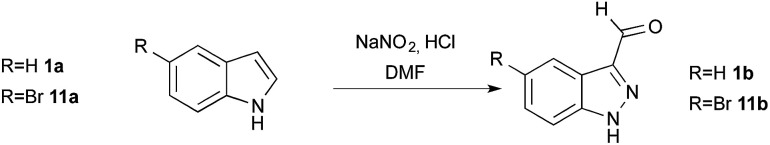					
Entry	Indole	Addition time	Addition temp. (°C)	Stoichiometry (NaNO_2_ : HCl)	Yield
1	1a	Rapid	rt	8 : 2.7	0%^a^
2	11a	Rapid	rt	8 : 2.7	13%^a^
3	1a	Rapid	0	8 : 2.7	0%^a^
4	11a	Rapid	0	8 : 2.7	41%^a^
5	1a	30 min	rt	8 : 2.7	5%^a^
6	11a	30 min	rt	8 : 2.7	19%^a^
7	1a	30 min	0	8 : 2.7	40%^a^
8	11a	30 min	0	8 : 2.7	72%^a^
9	1a	1 h	0	8 : 2.7	48%^a^
10	11a	1 h	0	8 : 2.7	>95%^a^
11	1a	2 h	0	8 : 2.7	99%^b^
12	11a	2 h	0	8 : 2.7	94%^b^
13	1a	2 h	0	8 : 12	0%^b^
14	11a	2 h	0	8 : 12	46%^a^
15	1a	2 h	0	4 : 2.7	52%^b^
16	1a	2 h	0	8 : 7	69%^b^

a Yields determined by ^1^H NMR using piperonal internal standard.

b Isolated yield.

Indole 1a and its 5-bromo derivative 11a were first reacted at room temperature with a nitrosating mixture composed of 8 equivalents of NaNO_2_ and 2.7 equivalents of HCl in DMF : water. While this procedure gave no product with indole 1a (Table 1, entry 1), a modest yield of 13% of 5-bromo-1*H*-indazole-3-carboxaldehyde 11b was obtained from 5-bromo-indole 11a (entry 2). Reducing the temperature to 0 °C, also failed to give the desired indazole from indole 1a, while 5-bromo-indole 11a gave the corresponding product in 41% yield (entries 3–4). In the latter experiment, dimers were the main products isolated as deep red compounds as previously described (Scheme 1, path b).^36–39^ Addressing this issue, a slow addition of indole to the nitrosating mixture was probed in order to maintain a low concentration of the nucleophilic indole during the second step of the reaction, to favor the trapping of intermediate 2 by water. Slow addition of indole 1a over 30 min at room temperature provided a 5% yield of indazole (entry 5) whereas at 0 °C, an encouraging improvement to 40% yield was obtained (entry 7). 5-Bromo-indole 11a followed the same trend with yields increasing from 19% to 72% (entries 6 and 8, respectively). When addition was performed over 1 h (entry 9), 48% of indazole 1b was obtained and the reaction was almost quantitative with indole 11a (entry 10). An even slower addition over 2 h was necessary to reach 99% yield with indole 1a and 94% yield with 11a (entries 11 and 12, respectively).

Having determined the preferred addition rate, we next examined the influence of the stoichiometry of the reagents. The chemistry of nitrogen oxide is complex due to the existence of numerous oxidation states (from 1/2 to 5)^40^ and nitrosations are usually achieved under strongly acidic conditions. However, in entries 1–12, the reactions were conducted with an excess of NaNO_2_ (8 equiv.) with respect to HCl (2.7 equiv.), and the pH value before and after the addition of indole was acidic (3.5 and 4.9, respectively).

**Scheme 2:**
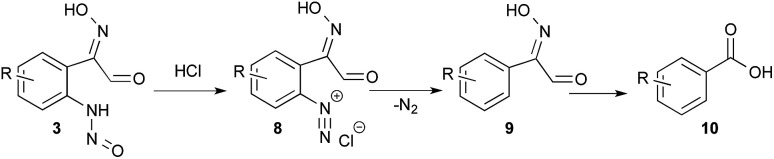
Dediazoniation process in acidic conditions, followed by oxidation as a plausible explanation to the formation of carboxylic acid side product.

When an excess of HCl (12 equiv.) was used, the pH was lower than 1 before and after the addition of indole; no indazole was isolated from indole 1a (entry 13) and only 46% from 5-bromo-indole 11a (entry 14). In this latter experiment 30% of *m*-bromo-benzoic acid (10) was also isolated. This side reaction could be explained from the *N*-nitrosoaniline intermediate 3 (Scheme 2). Indeed, under acidic conditions intermediate 3 can evolve to a highly reactive diazonium chloride salt leading to dediazoniation, then the remaining phenyl glyoxal oxime derivative 9 may undergo an oxidative decarboxylation to the corresponding carboxylic acid.^41^

When lowering the amount of NaNO_2_ from 8 to 4 equivalents, keeping the amount of HCl unchanged at 2.7 equivalents (entry 15), only 52% yield of indazole 1b was obtained. Similarly, when the amount of HCl was raised to 7 equiv. (pH value evolved from 3.5 to 4.2) only 69% of indazole 1b was isolated (entry 16).

Such a high variation of reactivity points out the complexity of the chemistry of nitrogen oxides, as modification of the stoichiometry of NaNO_2_ and HCl influences the pH value, and therefore also changes the nature of the nitrosating agents (hydrated nitrosonium ion H_2_ONO^+^, dinitrogen trioxide N_2_O_3_ or NOCl).^40,42–44^ Unfortunately, discrimination between these species is difficult as they can be in equilibrium.

**Fig. 2:**
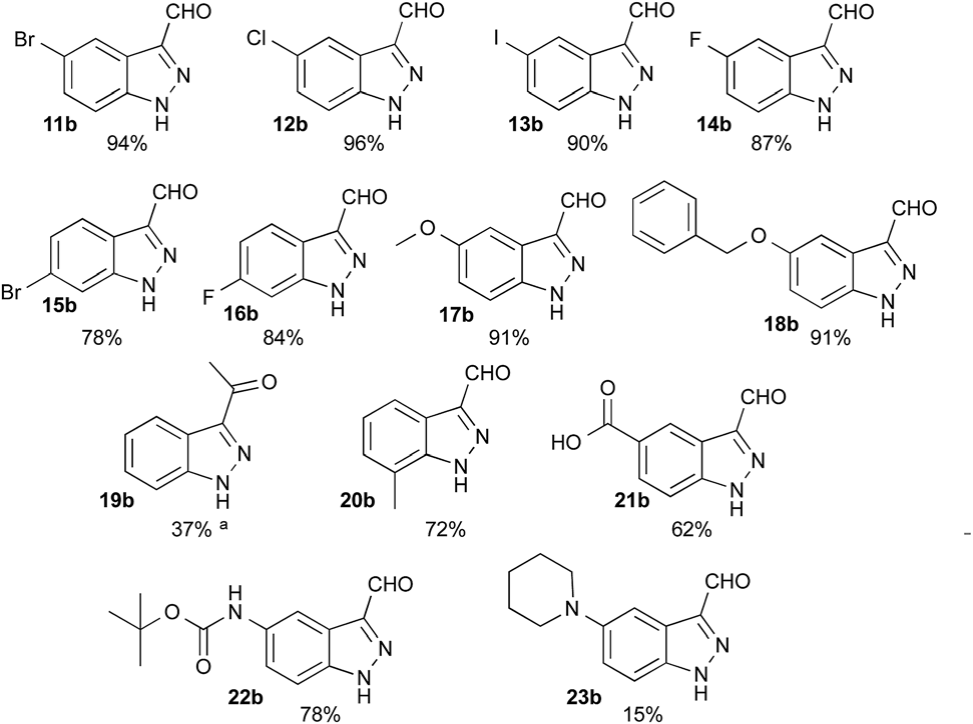
Scope of the reaction with corresponding isolated yields. Slow addition (2 h) at 0 °C of (1 equiv.) of the corresponding indole in DMF to a solution of NaNO_2_ (8 equiv.) and HCl (2.7 equiv.) in water : DMF 5.3 : 3. ^a^After addition, reaction was performed at 50 °C for 48 h.

Having defined the optimum conditions as: 8 equiv. of NaNO_2_, 2.7 equiv. of HCl and slow addition (2 h) of indole to the nitrosating mixture at 0 °C followed by stirring at room temperature, the scope of this reaction was then studied (Fig. 2). Indoles 11a–16a bearing halogens at position 5 or 6 afforded the corresponding indazoles 11b–16b with yields ranging from 78% to 96%. For these substrates, TLC analysis during addition at 0 °C showed consumption of the starting materials and the appearance of several products such as 2, 3 or 4. These intermediates were all converted to the corresponding 1*H*-indazole-3-carboxaldehyde after appropriate times (see Experimental section). Alternatively, heating at 50 °C for 3 to 5 h after the addition was complete reduced the reaction time, without affecting the yield. Indoles bearing electron-donating substituents such as methoxy 17a and benzyloxy 18a both provided indazoles in high yields (91%). Interestingly, this method allowed us to obtain the keto-indazole 19b from 2-methylindole 19a in a moderate 37% yield after heating 48 h at 50 °C.

The suitability of this procedure for substrates containing acid sensitive functional groups such as 5-NHBoc-indole 22a was probed, providing the corresponding 1*H*-indazole-3-carboxaldehyde 22b in good yield 78% without affecting the Boc group. On the other hand, 5-piperidyl-indole 23a was converted to its 5-piperidyl-1*H*-indazole-3-carboxaldehyde 23b in only 15%, major by-products being the 4-nitro-5-piperidyl-1*H*-indazole-3-carboxaldehyde and dimers.

Using this protocol, indoles bearing electron-withdrawing groups such as 5-CHO 24a or 5-CN 25a displayed moderate reactivity with 56% and 57% yields of the respective products (Table 2). Indeed, the indoles were only partially consumed during the addition time at 0 °C, and then generated dimers, negating the benefit of slow addition. Consequently, the temperature of addition for these slightly electron deficient indoles was investigated; at room temperature 5-CHO 24a and 5-CN 25a provided better yields with 65% (entry 3) and 66% (entry 4), respectively, but starting material still remained after the end of the addition. Increasing the amount of acid to 7 equiv. to generate more nitrosonium ion did not give a complete solution to the problem (entry 5 and 6). When the addition temperature was increased to 50 °C, a lower yield was obtained (entries 7 and 8) due to degradation of the nitrosating mixture at this temperature.^42,45^ Finally, to overcome this degradation, the rapid addition 24a and 25a was probed, but this produced large amounts of dimers, yielding only 45% of 24b and 34% of 25b (entry 9 and 10).

**Table 2:** Optimisation of the reaction conditions for electron-deficient indoles. Addition of indole (1 equiv.) to the nitrosating mixture containing NaNO_2_, HCl in water : DMF 5.3 : 3

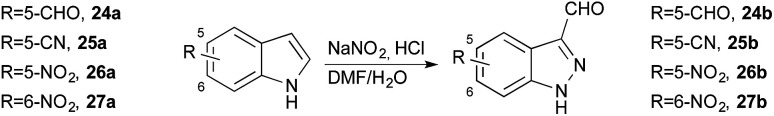					
Entry	Indole	Addition time	Addition : reaction temperature (°C)	Stoichiometry (NaNO_2_ : HCl)	Yield^a^
1	24a	2 h	0 : 50	8 : 2.7	56%
2	25a	2 h	0 : 50	8 : 2.7	57%
3	24a	2 h	rt : 50	8 : 2.7	66%
4	25a	2 h	rt : 50	8 : 2.7	65%
5	24a	2 h	rt : 50	8 : 7	73%
6	25a	2 h	rt : 50	8 : 7	60%
7	24a	2 h	50 : 50	8 : 7	39%
8	25a	2 h	50 : 50	8 : 7	16%
9	24a	Rapid	50 : 50	8 : 7	45%
10	25a	Rapid	50 : 50	8 : 7	34%
11	26a	2 h	rt : 50	8 : 2.7	0%
12	26a	Rapid	50 : 50	8 : 7	29%
13	26a	Rapid	80 : 80	8 : 7	99%
14	27a	Rapid	80 : 80	8 : 7	75%

a Isolated yield.

Nitro indoles were even less reactive, as no reaction occurred during the two-hour addition at room temperature for 5-nitro-indole 26a (entry 11). Therefore, rapid addition of indole was undertaken at 50 °C using 7 equivalents of HCl; the reaction was still very slow and didn't reach completion after 12 h (entry 12). Further increasing the reaction temperature to 80 °C proved to be effective and allowed us to obtain a quantitative yield of 26b (entry 13) and 75% of 27b (entry 14).

## Conclusions

A straightforward and efficient access to substituted 1*H*-indazole-3-carboxaldehyde derivatives is presented starting from readily accessible indoles. Nitrosation of indoles was previously described but no systematic study had been reported. Here we demonstrated the versatility of a new procedure, which can be adapted to a large variety of functionalized indoles, even those bearing acid-sensitive functionality. In general, electron-rich indoles proved to be very reactive toward the nitrosating mixture, producing reactive intermediates 2–4 rapidly even at 0 °C; the latter being converted to the corresponding indazole at room temperature. On the other hand, electron-neutral or slightly electron-deficient indoles required higher temperatures to generate reactive intermediates 2–4 and 50 °C for the latter to be converted to the indazole. At the other end of the series, electron-poor indoles such as nitro derivatives required 80 °C for full conversion to indazoles.

The reverse addition procedure depicted here provides a straightforward and high yielding conversion of indoles to their corresponding 1*H*-indazole-3-carboxaldehydes, despite the complexity of nitrogen oxides chemistry. This improved synthetic protocol should find applications in medicinal chemistry considering the emergence of indazole as pharmacophore.

## Experimental

### Materials and methods

Column chromatography purifications were performed on silica gel (40–63 μm). Thin-layer chromatography (TLC) analyses were carried out on Merck DC Kieselgel 60 F-254 aluminum sheets. The spots were visualized through illumination with UV lamp (*λ* = 254 nm and 360 nm) and/or staining with 4-hydrazinobenzenesulfonic acid. IR spectra were recorded with a universal ATR sampling accessory. ^1^H and ^13^C NMR spectra (C13APT or C13CPD experiments) were recorded on a 300 MHz spectrometer. Chemical shifts are expressed in parts per million (ppm) from the residual non-deuterated solvent signal contained in CDCl_3_ (*δ*_H_ = 7.26, *δ*_C_ = 77.16), in acetone-*d*_6_ (*δ*_H_ = 2.05, *δ*_C_ = 29.84) and in DMSO-*d*_6_ (*δ*_H_ = 2.50, *δ*_C_ = 39.52). Multiplicities are described as s (singlet), d (doublet), t (triplet), brs (broad peak) *etc.* Coupling constants, *J* values, are reported in Hz. High-resolution mass spectra (HRMS) were obtained using an orthogonal acceleration time-of-flight (oa-TOF) mass spectrometer equipped with an electrospray source and in the positive and negative modes (ESI+/−).

#### General procedure for 1 mmol of indole

To a solution of NaNO_2_ (550 mg, 8 mmol, 8 equiv.) in 4 mL of deionized water and 3 mL of DMF at 0 °C was added slowly HCl (1.33 mL of 2 N aq., 2.7 mmol, 2.7 equiv.) and the resulting mixture was kept under argon for 10 min. A solution of indole (1 mmol, 1 equiv.) in DMF (3 mL) was then added at 0 °C over a period of 2 h using a syringe pump. After addition, the reaction was continued depending on the starting indole used (see below).

### 1*H*-indazole-3-carboxaldehyde (1b)

The general procedure was used starting from (351 mg, 3 mmol) of indole 1a. After addition, the reaction was stirred 3 h at room temperature. The product was purified by column chromatography on silica gel, eluting with petroleum ether/EtOAc, 8 : 2 to provide the pure compound as a white solid (435 mg, 99%). Silica gel TLC *R*_f_ 0.37 (petroleum ether/EtOAc, 3 : 2); mp 141 °C; ^1^H NMR (300 MHz, DMSO-*d*_6_) *δ* 14.17 (brs, 1H), 10.20 (s, 1H), 8.14 (d, *J* = 8.5 Hz, 1H), 7.70 (d, *J* = 8.5 Hz, 1H), 7.49 (dt, *J* = 7.0, 1.0 Hz, 1H), 7.37 (dt, *J* = 7.0, 1.0 Hz, 1H); ^13^C NMR (75 MHz, DMSO-*d*_6_) *δ* 187.4, 143.4, 141.1, 127.3, 123.8, 120.7, 120.2, 111.2; IR (neat) *ν* = 3254, 3174, 1671, 1458, 1331, 1251, 1092, 792 and 739 cm^−1^; HRMS (ESI−), *m*/*z* calculated for C_8_H_5_N_2_O [M − H]^−^ 145.0390, found: 145.0402.

### 5-Bromo-1*H*-indazole-3-carboxaldehyde (11b)

The general procedure was used starting from (4.95 g, 25 mmol) of 5-bromo-indole 11a. After addition, the reaction was stirred 2 h at room temperature, then heated for 3 h at 50 °C. The product was purified by column chromatography on silica gel, eluting with petroleum ether/EtOAc, 8 : 2 to provide the pure compound as a brown solid (5.35 g, 94%). Silica gel TLC *R*_f_ 0.31 (petroleum ether/EtOAc, 3 : 2); mp 222 °C; ^1^H NMR (300 MHz, CDCl_3_) *δ* 10.53 (brs, 1H), 10.26 (s, 1H), 8.52 (d, *J* = 1.0 Hz, 1H), 7.59 (dd, *J* = 9.0, 1.5 Hz, 1H), 7.46 (d, *J* = 9.0 Hz, 1H); ^13^C NMR (75 MHz, DMSO-*d*_6_) *δ* 187.5, 144.5, 141.3, 131.3, 124.3, 123.1, 117.6, 113.8. IR (neat) *ν* = 3213, 2924, 1663, 1455, 1344, 1103, 922, 798 and 683 cm^−1^; HRMS (ESI−), *m*/*z* calculated for C_8_H_4_N_2_O^79^Br [M^79^Br − H]^−^ 222.9507, found: 222.9507.

### 5-Chloro-1*H*-indazole-3-carboxaldehyde (12b)

The general procedure was used starting from (303 mg, 2 mmol) of 5-chloro-indole 12a. After addition, the reaction was stirred 12 h at room temperature. The product was purified by column chromatography on silica gel, eluting with petroleum ether/EtOAc, 8 : 2 to provide the pure compound as a brown solid (344 mg, 96%). Silica gel TLC *R*_f_ 0.40 (petroleum ether/EtOAc, 3 : 2); mp 216 °C; ^1^H NMR (300 MHz, acetone-*d*_6_) *δ* 13.28 (brs, 1H), 10.19 (s, H), 8.16 (dd, *J* = 2.0, 0.5 Hz, 1H), 7.75 (dd, *J* = 9.0, 0.5 Hz, 1H), 7.47 (dd, *J* = 9.0 Hz, *J* = 2.0 Hz, 1H); ^13^C NMR (75 MHz, acetone-*d*_6_) *δ* 187.5, 144.6, 141.0, 130.0, 128.8, 122.5, 121.1, 113.5 ppm; IR (neat) *ν* = 3243, 1659, 1447, 1333, 1100, 926, 798 and 685 cm^−1^; HRMS (ESI−), *m*/*z* calculated for C_8_H_4_^35^ClN_2_O [M − H]^−^ 179.0012, found: 179.0012.

### 5-Iodo-1*H*-indazole-3-carboxaldehyde (13b)

The general procedure was used starting from (486 mg, 2 mmol) of 5-iodo-indole 13a. After addition, the reaction was stirred 8 h at room temperature. The resulting mixture was extracted with EtOAc three times, washed three times with water, then with brine, dried over MgSO_4_ and concentrated under reduced pressure. The resulting crude mixture was purified by column chromatography on silica gel, eluting with petroleum ether/EtOAc, 8 : 2 to provide the pure compound as a brown solid (487 mg, 90%). Silica gel TLC *R*_f_ 0.40 (petroleum ether/EtOAc, 3 : 2); mp 260 °C; ^1^H NMR (300 MHz, DMSO-*d*_6_) *δ* 14.28 (brs, 1H), 10.16 (s, H), 8.46 (d, *J* = 0.9 Hz, 1H), 7.73 (dd, *J* = 8.7, *J* = 1.6 Hz, 1H), 7.56 (dd, *J* = 8.7, *J* = 0.5 Hz, 1H); ^13^C NMR (75 MHz, DMSO-*d*_6_) *δ* 187.2, 142.4, 140.2, 135.3, 129.1, 122.6, 113.5, 88.6; IR (neat) *ν* = 3179, 1669, 1446, 1305, 1098, 915, 794, 773, 746 and 676 cm^−1^; HRMS (ESI), calculated for C_8_H_4_N_2_OI [M − H]^−^*m*/*z* 270.9368, found: 270.9370.

### 5-Fluoro-1*H*-indazole-3-carboxaldehyde (14b)

The general procedure was used starting from (270 mg, 2 mmol) of 5-fluoro-indole 14a. After addition, the reaction was stirred 5 h at room temperature. The product was purified by column chromatography on silica gel, eluting with petroleum ether/EtOAc, 8 : 2 to provide the pure compound as a yellowish solid (284 mg, 87%). Silica gel TLC *R*_f_ 0.35 (petroleum ether/EtOAc, 3 : 2); mp 170 °C; ^1^H NMR (300 MHz, acetone-*d*_6_) *δ* 13.20 (brs, 1H), 10.19 (s, 1H), 7.74–7.84 (m, 2H), 7.34 (td, *J* = 9.1, 2.5 Hz, 1H); ^13^C NMR (75 MHz, acetone-*d*_6_) *δ* 187.4, 160.5 (d, ^1^*J*_(C–F)_ = 238 Hz), 145.1, 139.4, 121.9 (d, ^3^*J*_(C–F)_ = 11 Hz), 117.6 (d, ^2^*J*_(C–F)_ = 27 Hz), 113.49 (d, ^3^*J*_(C–F)_ = 9.7 Hz), 105.9 (d, ^2^*J*_(C–F)_ = 25 Hz); IR (neat) *ν* = 3315, 3185, 1683, 1656, 1486, 1446, 1344, 1316, 1175, 1062, 952, 782 and 735 cm^−1^; HRMS (ESI−) calculated for C_8_H_4_FN_2_O [M − H]^−^*m*/*z* 163.0308, found: 163.0304.

### 6-Bromo-1*H*-indazole-3-carboxaldehyde (15b)

The general procedure was used starting from (196 mg, 3 mmol) of 6-bromo-indole 15a. After addition, the reaction was stirred 2 h at room temperature, then 3 h at 50 °C. The resulting mixture was extracted with EtOAc three times, washed three times with water, then with brine, dried over MgSO_4_ and concentrated under reduced pressure. The resulting crude mixture was purified by column chromatography on silica gel, eluting with petroleum ether/EtOAc, 8 : 2 to provide the pure compound as a brown solid (175 mg, 78%). Silica gel TLC *R*_f_ 0.43 (petroleum ether/EtOAc, 3 : 2); mp 229 °C; ^1^H NMR (300 MHz, CDCl_3_) *δ* 10.49 (brs, 1H), 10.26 (s, 1H), 8.20 (dd, *J* = 8.5, 0.5 Hz, 1H), 7.76 (dd, *J* = 1.5, 0.5 Hz, 1H), 7.49 (dd, *J* = 8.5, 1.5 Hz, 1H); ^13^C NMR (75 MHz, DMSO-*d*_6_) *δ* 187.4, 143.5, 141.2, 127.3, 123.8, 120.7, 120.3, 111.1. IR (neat) *ν* = 3342, 2980, 1671, 1597, 1480, 1281, 1196, 1064, 1034, 791, 744 and 492 cm^−1^; HRMS (ESI−), calculated for C_8_H_4_N_2_O^79^Br [M − H]^−^*m*/*z* 222.9507, found: 222.9498.

### 6-Fluoro-1*H*-indazole-3-carboxaldehyde (16b)

The general procedure was used starting from (270 mg, 2 mmol) of 6-fluoro-indole 16a. After addition, the reaction was stirred 5 h at room temperature. The resulting mixture was extracted with EtOAc three times, washed three times with water, then with brine, dried over MgSO_4_ and concentrated under reduced pressure. The resulting crude mixture was purified by column chromatography on silica gel, eluting with petroleum ether/EtOAc, 8 : 2 to provide the pure compound as a yellowish solid (277 mg, 84%). Silica gel TLC *R*_f_ 0.42 (petroleum ether/EtOAc, 3 : 2); mp 186 °C; ^1^H NMR (300 MHz, acetone-*d*_6_) *δ* 13.13 (brs, 1H), 10.20 (s, 1H), 8.22 (dd, *J* = 9.0, 5.0 Hz, 1H), 7.45 (ddd, *J* = 9.0, 2.0, 0.5 Hz, 1H), 7.20 (ddd, *J* = 9.5, 9.0, 2.0 Hz, 1H); ^13^C NMR (75 MHz, acetone-*d*_6_) *δ* 187.6, 163.2 (d, *J* = 244 Hz), 145.2, 142.9 (d, *J* = 13 Hz), 123.8 (d, *J* = 11 Hz), 118.5, 114.2 (d, *J* = 26 Hz), 97.4 (d, *J* = 27 Hz). IR (neat) *ν* = 3142, 1695, 1675, 1633, 1463, 1333, 1149, 862, 807, and 727 cm^−1^; HRMS (ESI−) calculated for C_8_H_4_FN_2_O, [M − H]^−^*m*/*z* 163.0308, found: 163.0304.

### 5-Methoxy-1*H*-indazole-3-carboxaldehyde (17b)

The general procedure was used starting from (441 mg, 3 mmol) of 5-methoxy-indole 17a. After addition, the reaction was stirred 3 h at room temperature. The resulting mixture was extracted with EtOAc three times, washed three times with water, then with brine, dried over MgSO_4_ and concentrated under reduced pressure. The resulting crude mixture was purified by column chromatography on silica gel, eluting with petroleum ether/EtOAc, 8 : 2 to provide the pure compound as a yellowish solid (480 mg, 91%). Silica gel TLC *R*_f_ 0.29 (petroleum ether/EtOAc, 3 : 2); mp 215 °C; ^1^H NMR (300 MHz, DMSO-*d*_6_) *δ* 14.06 (brs, 1H), 10.16 (s, 1H), 7.61 (d, *J* = 9.0 Hz, 1H), 7.49 (d, *J* = 2.5 Hz, 1H), 7.12 (dd, *J* = 9.0, 2.5 Hz, 1H), 3.84 (s, 3H); ^13^C NMR (75 MHz, DMSO-*d*_6_) *δ* = 187.3, 156.6, 143.1, 136.9, 121.3, 119.3, 112.3, 99.7, 55.4; IR (neat) *ν* = 3201, 1663, 1452, 1258, 1216, 1076, 795, 765, and 719 cm^−1^; HRMS (ESI−), calculated for [M − H]^−^*m*/*z* 175.0508, found: 175.0503.

### 5-Benzyloxy-1*H*-indazole-3-carboxaldehyde (18b)

The general procedure was used starting from (446 mg, 3 mmol) of 5-benzyloxy-indole 18a. After addition, the reaction was stirred 3 h at room temperature. The resulting mixture was extracted with EtOAc three times, washed three times with water, then with brine, dried over MgSO_4_ and concentrated under reduced pressure. The resulting crude mixture was purified by column chromatography on silica gel, eluting with petroleum ether/EtOAc, 8 : 2 to provide the pure compound without any further purification as a brownish solid (457 mg, 91%). Silica gel TLC *R*_f_ 0.40 (petroleum ether/EtOAc, 3 : 2); mp 230 °C; ^1^H NMR (300 MHz, DMSO-*d*_6_) *δ* 14.11 (brs, 1H), 10.16 (s, 1H), 7.63 (m, 2H), 7.49 (m, 2H), 7.30–7.45 (m, 3H), 7.22 (dd, *J* = 2.3, 9.0 Hz, 1H), 5.17 (s, 2H); ^13^C NMR (75 MHz, DMSO-*d*_6_) *δ* 187.2, 155.6, 143.0, 137.1, 136.9, 128.4, 127.8, 127.7, 121.2, 119.7, 112.5, 101.2, 69.6; IR (neat) *ν* = 3247, 1656, 1456, 1263, 1226, 1072, 1005, 948, 793 and 724 cm^−1^; HRMS (ESI−), calculated for C_15_H_11_N_2_O_2_ [M − H]^−^*m*/*z* 251.0821, found: 251.0817.

### 1-(1*H*-indazol-3-yl)ethanone (19b)

The general procedure was used starting from (262 mg, 2 mmol) of 3-methyl-indole 19a. After addition, the reaction was stirred 48 h at 50 °C. The resulting mixture was extracted with EtOAc three times, washed three times with water, then with brine, dried over MgSO_4_ and concentrated under reduced pressure. The resulting crude mixture was purified by column chromatography on silica gel, eluting with petroleum ether/EtOAc, 8 : 2 to provide the pure compound as a brown solid (118 mg, 37%). Silica gel TLC *R*_f_ 0.45 (petroleum ether/EtOAc, 3 : 2); mp 172 °C; ^1^H NMR (300 MHz, acetone-*d*_6_) *δ* 12.84 (brs, 1H), 8.29 (dt, *J* = 8.0, 1.0 Hz, 1H), 7.68 (dt, *J* = 8.5, 1.0 Hz, 1H), 7.44 (ddd, *J* = 8.5, 7.0, 1.0 Hz, 1H), 7.31 (ddd, *J* = 8.0, 7.0, 1.0 Hz, 1H), 2.66 (s, 3H); ^13^C NMR (75 MHz, acetone-*d*_6_) *δ* 195.0, 144.5, 142.5, 127.7, 124.0, 122.8, 122.4, 111.4, 26.7; IR (neat) *ν* = 3212, 3191, 1653, 1448, 1342, 1210, 1156, 954, 750 and 610 cm^−1^; HRMS (ESI−), calculated for C_9_H_7_N_2_O [M − H]^−^*m*/*z* 159.0558, found: 159.0556.

### 7-Methyl-1*H*-indazole-3-carboxaldehyde (20b)

The general procedure was used starting from (262 mg, 2 mmol) of 7-methyl-indole 20a. After addition, the reaction was stirred 12 h at room temperature. The resulting mixture was extracted with EtOAc three times, washed three times with water, then with brine, dried over MgSO_4_ and concentrated under reduced pressure. The resulting crude mixture was purified by column chromatography on silica gel, eluting with petroleum ether/EtOAc 8 : 2 to provide the pure compound as a yellowish solid (229 mg, 72%). Silica gel TLC *R*_f_ 0.45 (petroleum ether/EtOAc, 3 : 2); mp 172 °C; ^1^H NMR (300 MHz, CDCl_3_) *δ* 10.31 (s, 1H), 8.14 (m, 1H), 7.28 (m, 2H), 2.62 (s, 3H); ^13^C NMR (75 MHz, CDCl_3_) *δ* 187.7, 145.3, 141.5, 128.2, 124.7, 120.9, 120.2, 119.5, 16.8; IR (neat) *ν* = 3252, 3077, 1672, 1449, 1326, 1144, 784 and 734 cm^−1^; HRMS (ESI−), calculated for C_9_H_7_N_2_O [M − H]^−^*m*/*z* 159.0558, found: 159.0549.

### 5-Carboxy-1*H*-indazole-3-carboxaldehyde (21b)

The general procedure was used starting from (483 mg, 3 mmol) of 5-carboxy-indole 21a. After addition, the reaction was stirred 2 h at 50 °C. The product was purified by centrifugation to provide (236 mg, 62%) of a white solid. Silica gel TLC *R*_f_ 0.21 (EtOAc); mp > 260 °C; ^1^H NMR (300 MHz, DMSO-*d*_6_) *δ* 14.63 (brs, 1H), 13.05 (br, 1H), 10.19 (s, 1H), 8.72 (s, 1H), 8.00 (d, *J* = 9.5, Hz, 1H), 7.76 (d, *J* = 9.5, 1H); ^13^C NMR (75 MHz, DMSO-*d*_6_) *δ* 187.5, 167.2, 144.3, 142.9, 127.8, 126.4, 123.3, 120.0, 111.3; IR (neat) *ν* = 3234, 2787, 1702, 1682, 1416, 1278, 1235, 829, 802, and 743 cm^−1^; HRMS (ESI−), calculated for C_9_H_4_N_2_O_3_ [M − H]^−^*m*/*z* 189.0300, found: 189.0296.

### 5-NHBoc-1*H*-indazole-3-carbaldehyde (22b)

The general procedure was used starting from (232 mg, 1 mmol) of 5-NHBoc-indole 22a. After addition, the reaction was stirred 3 h at room temperature. The resulting mixture was extracted with EtOAc three times, washed three times with water, then with brine, dried over MgSO_4_ and concentrated under reduced pressure. The product was purified by column chromatography on silica gel, eluting with CH_2_Cl_2_/EtOAc, 9 : 1 to provide the pure compound as a white solid (203 mg, 78%). Silica gel TLC *R*_f_ 0.26 (petroleum ether/EtOAc, 3 : 2); mp 181 °C; ^1^H NMR (300 MHz, acetone-*d*_6_) *δ* 13.06 (brs, 1H), 10.21 (s, 1H), 8.56 (br, 1NH), 8.53 (s, 1H), 7.64 (s, 2H), 1.50 (s, 9H); ^13^C NMR (75 MHz, acetone-*d*_6_) *δ* 187.5, 153.9, 145.0, 139.0, 137.0, 122.1, 121.6, 111.8, 109.6, 80.1, 28.5; IR (neat) *ν* = 3337, 2989, 1698, 1666, 1534, 1504, 1426, 1324, 1242, 1169, 1050, 908, 852, 813, 797, 723 and 641 cm^−1^; HRMS (ESI−), calculated for C_13_H_14_N_3_O_3_ [M − H]^−^*m*/*z* 260.1035, found: 260.1027.

### 5-Piperidyl-1*H*-indazole-3-carboxaldehyde (23b)

The general procedure was used starting from (200 mg, 1 mmol) of 5-piperidyl-indole 23a. After addition, the reaction was stirred 3 h at room temperature. The resulting mixture was extracted with EtOAc three times, washed three times with water, then with brine, dried over MgSO_4_ and concentrated under reduced pressure. The product was purified by column chromatography on silica gel, eluting with CH_2_Cl_2_ to provide the pure compound as a yellow solid (34 mg, 15%). Silica gel TLC *R*_f_ 0.23 (petroleum ether/EtOAc, 3 : 2); mp 75 °C; ^1^H NMR (300 MHz, CDCl_3_) *δ* 11.41 (brs, 1H), 10.27 (s, 1H), 7.66 (d, *J* = 2.0 Hz, 1H), 7.39 (d, *J* = 9.0 Hz, 1H), 7.26 (dd, *J* = 9.0, 2.0 Hz, 1H), 3.17 (m, 4H), 1.76 (m, 4H), 1.58 (m, 2H); ^13^C NMR (75 MHz, CDCl_3_) *δ* 187.6, 150.4, 144.2, 137.0, 122.8, 122.5, 110.8, 106.1, 52.3, 26.1, 24.3; IR (neat) *ν* = 2927, 2852, 1666, 1495, 1465, 1451, 1213, 1079, 949, 791 and 708 cm^−1^; HRMS (ESI−), calculated for C_13_H_14_N_3_O [M − H]^−^*m*/*z* 228.1137, found: 228.1129.

### 1*H*-indazole-3,5-dicarboxaldehyde (24b)

The general procedure was modified. To a solution of NaNO_2_ (550 mg, 8 mmol, 8 equiv.) in 1.6 mL of deionized water and 3 mL of DMF at 0 °C was added slowly HCl (3.5 mL of 2 N aq., 7 mmol, 7 equiv.). After 10 min at this temperature, a solution of indole-5-carbaldehyde 24a (145 mg, 1 mmol, 1 equiv.) in DMF (3 mL) was then added at room temperature over a period of 2 hours using a syringe pump. After addition, the reaction was stirred 2 h at room temperature, then 16 h at 50 °C. The resulting mixture was extracted with EtOAc three times, washed three times with water, then with brine, dried over MgSO_4_ and concentrated under reduced pressure. The resulting crude mixture was purified by column chromatography on silica gel, eluting with petroleum ether/EtOAc, 8 : 2 to provide the pure compound as a white solid (127 mg, 73%). Silica gel TLC *R*_f_ 0.28 (petroleum ether/EtOAc, 3 : 2); mp 242 °C; ^1^H NMR (300 MHz, DMSO-*d*_6_) *δ* 14.47 (brs, 1H), 10.22 (s, 1H), 10.09 (s, 1H), 8.68 (d, *J* = 1.5 Hz, 1H), 7.92 (dd, *J* = 8.5, 1.5 Hz, 1H), 7.80 (d, *J* = 8.5 Hz, 1H); ^13^C NMR (75 MHz, DMSO-*d*_6_) *δ* 192.6, 187.4, 144.8, 143.4, 132.5, 126.6, 125.6, 120.0, 112.1; IR (neat) *ν* = 3268, 1672, 1616, 1323, 1189, 1151, 1062, 836 and 793 cm^−1^; HRMS (ESI−), calculated for C_9_H_5_N_2_O_2_ [M − H]^−^*m*/*z* 173.0351, found: 173.0346.

### 5-Cyano-1*H*-indazole-3-carbaldehyde (25b)

The general procedure was modified. To a solution of NaNO_2_ (550 mg, 8 mmol, 8 equiv.) in 1.6 mL of deionized water and 3 mL of DMF at 0 °C was added slowly HCl (3.5 mL of 2 N aq., 7 mmol, 7 equiv.). After 10 min at this temperature, a solution of 5-cyano-indole 25a (142 mg, 1 mmol, 1 equiv.) in DMF (3 mL) was then added at room temperature over a period of 2 hours using a syringe pump. After addition, the reaction was stirred 2 h at room temperature, then 16 h at 50 °C. The resulting mixture was extracted with EtOAc three times, washed three times with water, then with brine, dried over MgSO_4_ and concentrated under reduced pressure. The resulting crude mixture was purified by column chromatography on silica gel, eluting with petroleum ether/EtOAc, 8 : 2 to provide the pure compound as a brown solid (103 mg, 60%). Silica gel TLC *R*_f_ 0.23 (petroleum ether/EtOAc, 3 : 2); mp 218 °C; ^1^H NMR (300 MHz, acetone-*d*_6_) *δ* 10.24 (s, 1H), 8.57 (m, 1H), 7.94 (dd, *J* = 8.5, 1.0 Hz, 1H), 7.79 (dd, *J* = 8.5, 1.5 Hz, 1H); ^13^C NMR (75 MHz, acetone-*d*_6_) *δ* 187.5, 145.5, 143.3, 130.4, 128.1, 121.1, 119.5, 113.6, 108.1; IR (neat) *ν* = 3292, 2230, 1674, 1621, 1417, 936, 825, 816 and 793 cm^−1^; HRMS (ESI−), calculated for C_9_H_4_N_3_O [M − H]^−^*m*/*z* 170.0354, found: 170.0352.

### 5-Nitro-1*H*-indazole-3-carboxaldehyde (26b)

To a solution of NaNO_2_ (1.65 g, 24 mmol, 8 equiv.) in 4.75 mL of deionized water at 0 °C was added slowly HCl (10.5 mL of 2 N aq., 21 mmol, 7 equiv.) and the resulting mixture was kept under argon for 10 min before adding 9 mL of DMF. A solution of 5-nitro-indole 26a (501 mg, 3 mmol, 1 equiv.) in DMF (9 mL) was then added at 0 °C. The reaction mixture was heated at 80 °C and stirred under argon for 6 h. The resulting mixture was extracted with EtOAc three times, washed with brine. The layers were separated and the aqueous layer was extracted with 25 mL of EtOAc, dried over MgSO_4_ and concentrated under reduced pressure. The resulting crude mixture was purified by column chromatography on silica gel, eluting with petroleum ether/EtOAc, 8 : 2 to provide the pure compound as a yellowish solid (568 mg, 99%). Silica gel TLC *R*_f_ 0.18 (petroleum ether/EtOAc, 3 : 2); mp 215 °C. ^1^H NMR (300 MHz, DMSO-*d*_6_) *δ* 14.65 (brs, 1H), 10.21 (s, 1H), 8.86 (d, *J* = 2.0 Hz, 1H), 8.26 (dd, *J* = 9.0 Hz, *J* = 2.0 Hz, 1H), 7.86 (d, *J* = 9.0, 1H); ^13^C NMR (75 MHz, DMSO-*d*_6_) *δ* 187.2, 145.1, 143.8, 142.9, 122.0, 119.36, 117.7, 112.4; IR (neat) *ν* = 3330, 1682, 1519, 1445, 1342, 1321, 1115, 855, 796, and 681 cm^−1^; HRMS (ESI−), calculated for C_8_H_4_N_3_O_3_ [M − H]^−^*m*/*z* 190.0253, found: 190.0243.

### 6-Nitro-1*H*-indazole-3-carboxaldehyde (27b)

To a solution of NaNO_2_ (550 mg, 8 mmol, 8 equiv.) in 1.6 mL of deionized water at 0 °C was added slowly (3.5 mL, 7 mmol, 7 equiv.) of HCl (2 N aq.) and the resulting mixture was kept under argon for 10 min before adding 3 mL of DMF. A solution of 6-nitroindole 27a (162 mg, 1 mmol, 1 equiv.) in DMF (3 mL) was then added at 0 °C. The reaction mixture was heated at 80 °C and stirred under argon for 6 h. The resulting mixture was extracted with EtOAc three times, washed with brine. The layers were separated and the aqueous layer was extracted with 25 mL of EtOAc, dried over MgSO_4_ and concentrated under reduced pressure. The resulting crude mixture was purified by column chromatography on silica gel, eluting with dichloromethane to provide the pure compound as a white solid (144 mg, 75%). Silica gel TLC *R*_f_ 0.34 (petroleum ether/EtOAc, 3 : 2); mp > 260 °C; ^1^H NMR (300 MHz, acetone-*d*_6_) *δ* 10.27 (s, 1H), 8.68 (d, *J* = 2.0 Hz, 1H), 8.39 (d, *J* = 9.0 Hz, 1H), 8.21 (dd, *J* = 9.0, 2.0 Hz, 2H); ^13^C NMR (75 MHz, acetone-*d*_6_) *δ* 187.5, 148.0, 145.1, 141.3, 124.6, 123.1, 119.0, 108.8; IR (neat) *ν* = 3293, 1668, 1519, 1423, 1347, 1310, 1062, 873, and 751 cm^−1^; HRMS (ESI−), calculated for C_8_H_4_N_3_O_3_ [M − H]^−^*m*/*z* 190.0253, found: 190.0245.

## Conflicts of interest

There are no conflicts to declare.

## Supplementary Material

RA-008-C8RA01546E-s001
