# Supplementary material for: An optimized procedure for direct access to 1H-indazole-3-carboxaldehyde derivatives by nitrosation of indoles
Source: RSC Adv. 2018 Apr 9;8(24):13121–8. doi: 10.1039/c8ra01546e (PMC9079728; doi:10.1039/c8ra01546e)
Supplement: RA-008-C8RA01546E-s001 [file RA-008-C8RA01546E-s001.pdf]

Electronic Supplementary Material (ESI) for RSC Advances.
This journal is © The Royal Society of Chemistry 2018

An optimized procedure for direct access to 1*H*-Indazole-3-carboxaldehyde derivatives by nitrosation of indoles.

Arnaud Chevalier, Abdelaaziz Ouahrouch, Alexandre Arnaud, Thibault Gallavardin* and Xavier Franck*

Normandie Univ, INSA Rouen, UNIROUEN, CNRS, COBRA, 76000 Rouen, France

Thibault Gallavardin, CNRS UMR 6014, C.O.B.R.A. & FR 3038, Université de Rouen, INSA de Rouen, 1 Rue Tesniere; 76131 Mont-Saint-Aignan cedex, France; Fax: (+33) 235522959; e-mail: thibault.gallavardin@univ-rouen.fr

Xavier Franck, CNRS UMR 6014, C.O.B.R.A. & FR 3038, Université de Rouen, INSA de Rouen, 1 Rue Tesniere; 76131 Mont-Saint-Aignan cedex, France; Fax: (+33) 235522959; e-mail: xavier.franck@insa-rouen.fr

I. Materials and Methods ……………………………………………………………………………………… p 1

II. Copies of 1H, 13C NMR and IR of compounds 1b-27b ……………………………………………… p 2

I. Materials and Methods

Commercially available reagents were used without further purification. Column chromatography purifications were performed on silica gel (40-63 µm). Thin-layer chromatography (TLC) analyses were carried out on Merck DC Kieselgel 60 F-254 aluminum sheets. The spots were visualized through illumination with UV lamp (λ = 254 nm and 360 nm) and/or staining with 4-hydrazinobenzenesulfonic acid. IR spectra were recorded with an ATR diamant Perkin Elmer. 1H and 13C NMR spectra (C13APT or C13CPD experiments) were recorded on a Bruker AVIII 300 MHz spectrometer (BBFO). Chemical shifts are expressed in parts per million (ppm) from the residual non-deuterated solvent signal contained in $CDCl_3$ (δH = 7.26, δC = 77.16), in Acetone-d_6 (δH = 2.05, δC = 29.84) and in DMSO-d_6 (δH = 2.50, δC = 39.52). Multiplicities are described as s (singlet), d (doublet), t (triplet), brs (broad peak) etc. Coupling constants, *J* values, are reported in Hz. High-resolution mass spectra (HRMS) were obtained using an orthogonal acceleration time-of-flight (oa-TOF) mass spectrometer equipped with an electrospray source and in the positive and negative modes (ESI+/-).

II. Copies of 1H and 13C NMR

1*H*-Indazole-3-carboxaldehyde (1b)

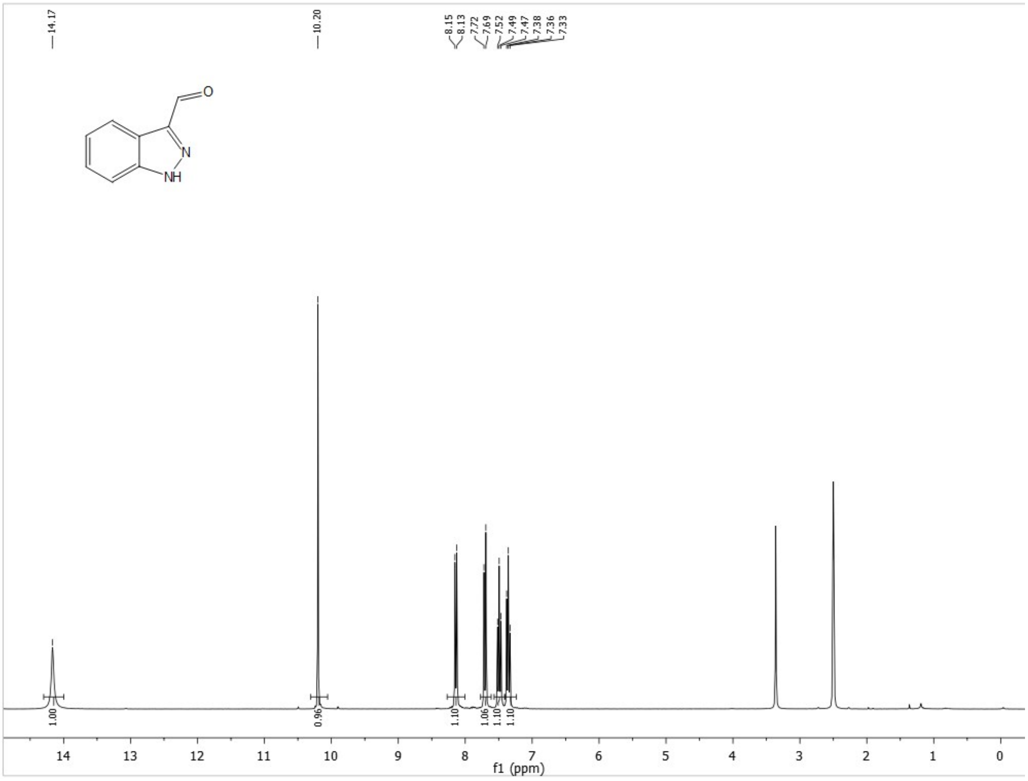

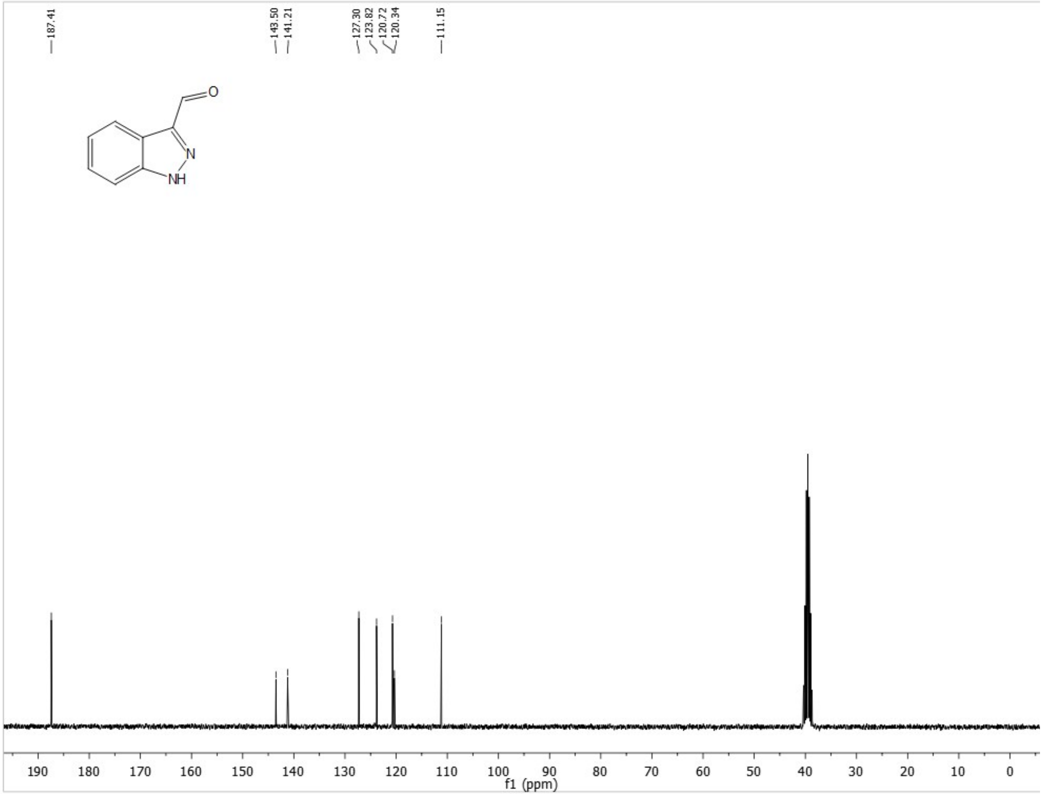

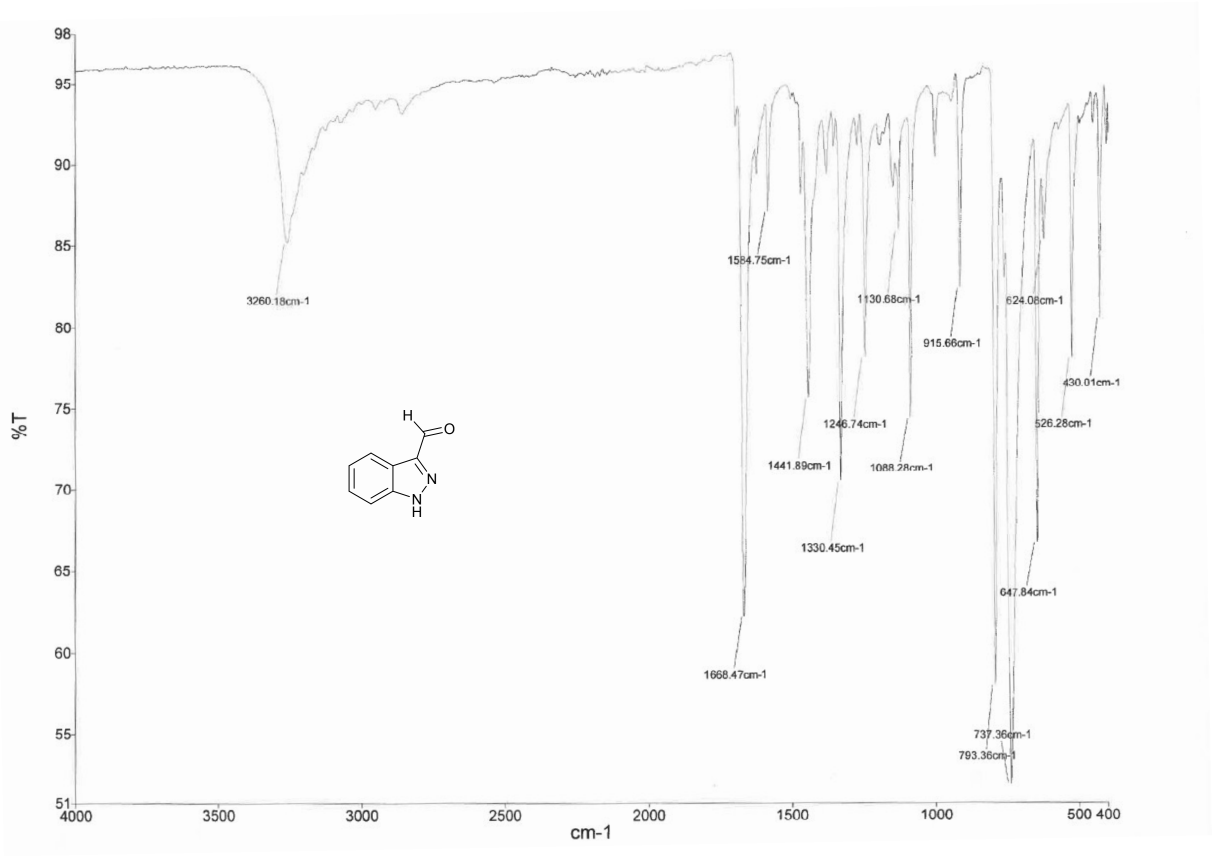

98
95
90
85
80
75
70
65
60
55
51
%T
4000
3500
3000
2500
2000
1500
1000
500 400
cm-1
3260.18cm-1
1584.75cm-1
1130.68cm-1
915.66cm-1
624.08cm-1
430.01cm-1
1246.74cm-1
526.28cm-1
1441.89cm-1
1088.28cm-1
1330.45cm-1
647.84cm-1
1668.47cm-1
737.36cm-1
793.36cm-1
H
O
N
N
H

5-Bromo-1*H*-Indazole-3-carboxaldehyde (11b)

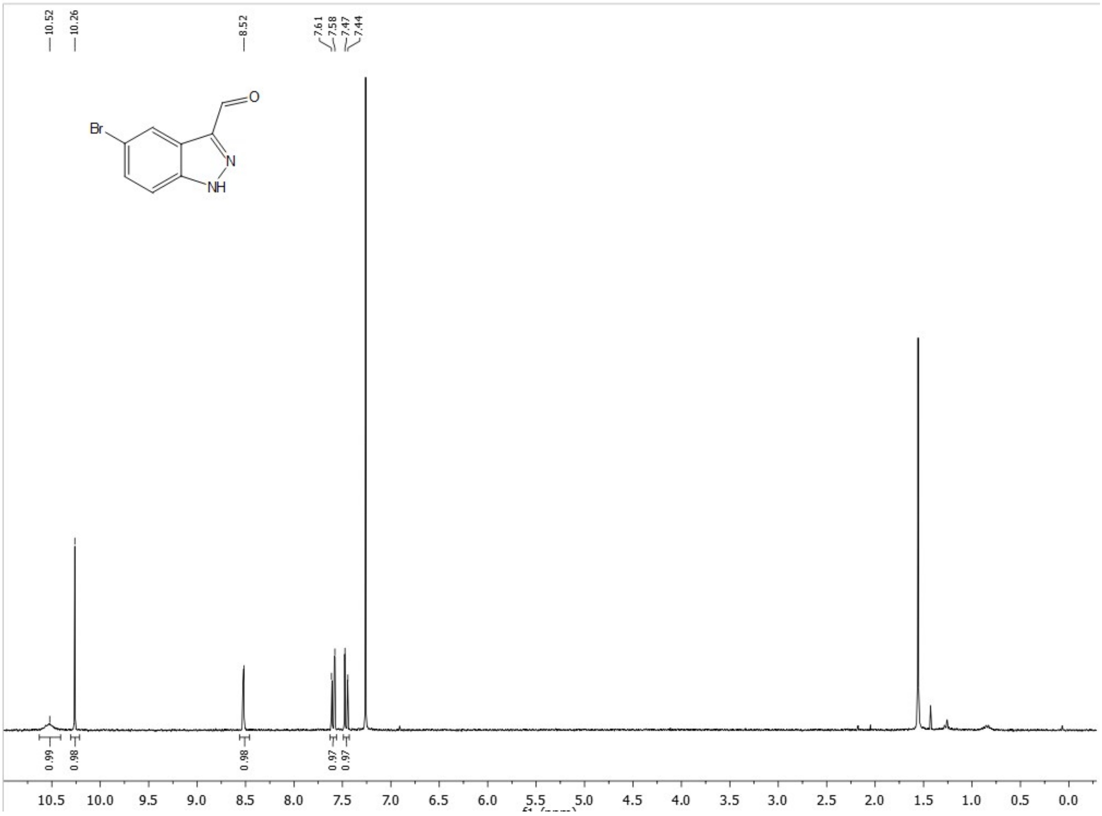

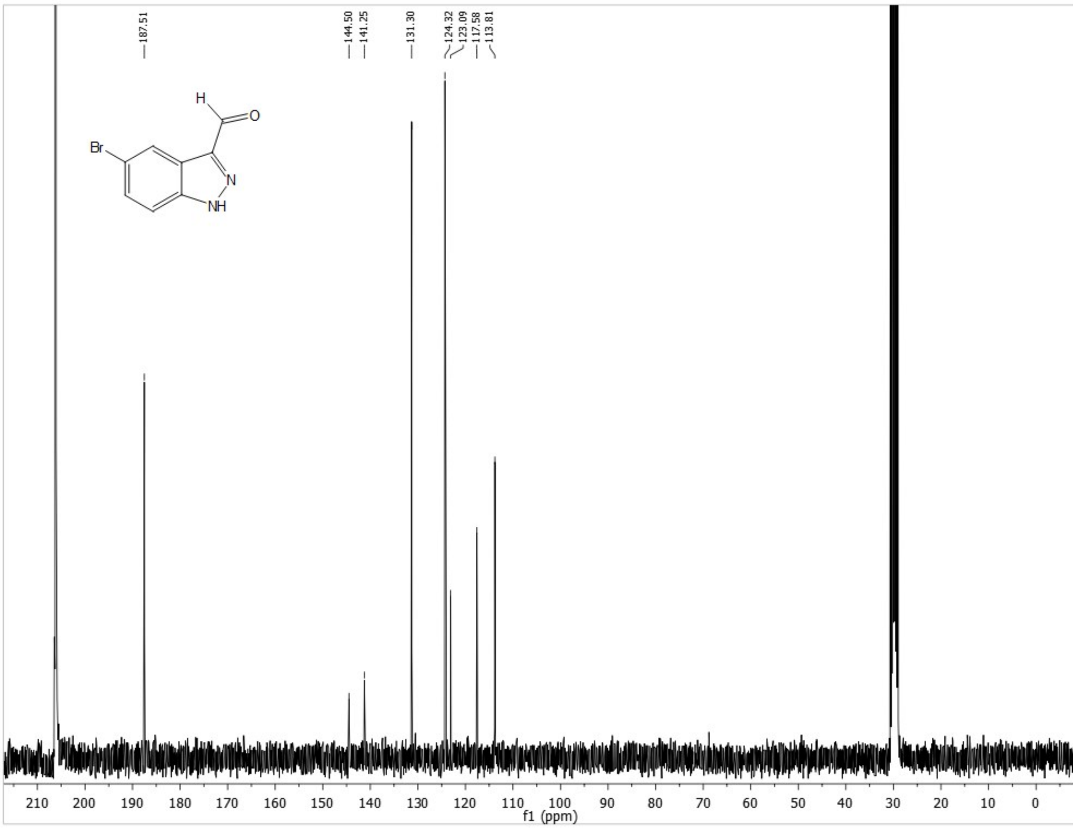

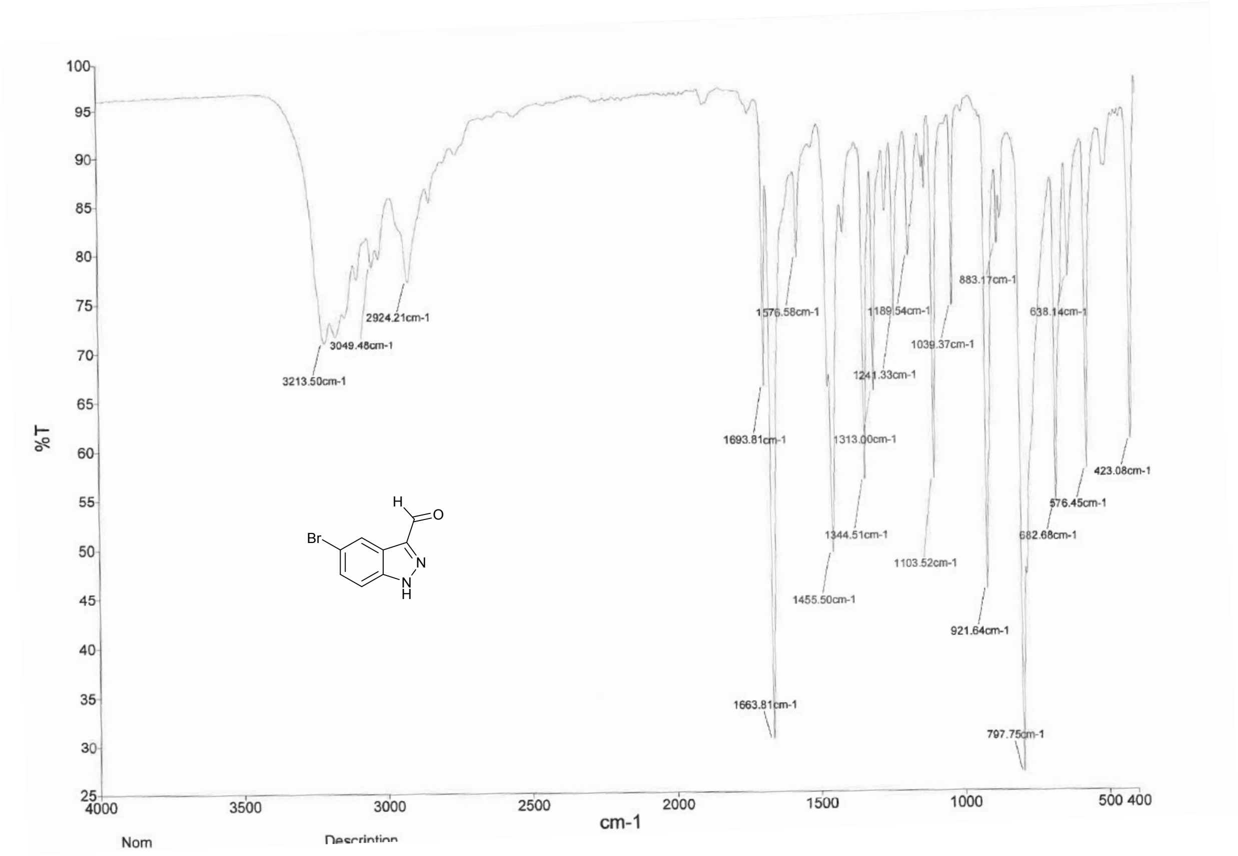
%T
cm-1
3213.50cm-1
3049.48cm-1
2924.21cm-1
1693.81cm-1
1663.81cm-1
1576.58cm-1
1455.50cm-1
1344.51cm-1
1313.00cm-1
1241.33cm-1
1189.54cm-1
1103.52cm-1
1039.37cm-1
921.64cm-1
883.17cm-1
797.75cm-1
682.68cm-1
638.14cm-1
576.45cm-1
423.08cm-1
Br
H
O
N
N
H
Nom

5-chloro-1*H*-indazole-3-carboxaldehyde (12b)

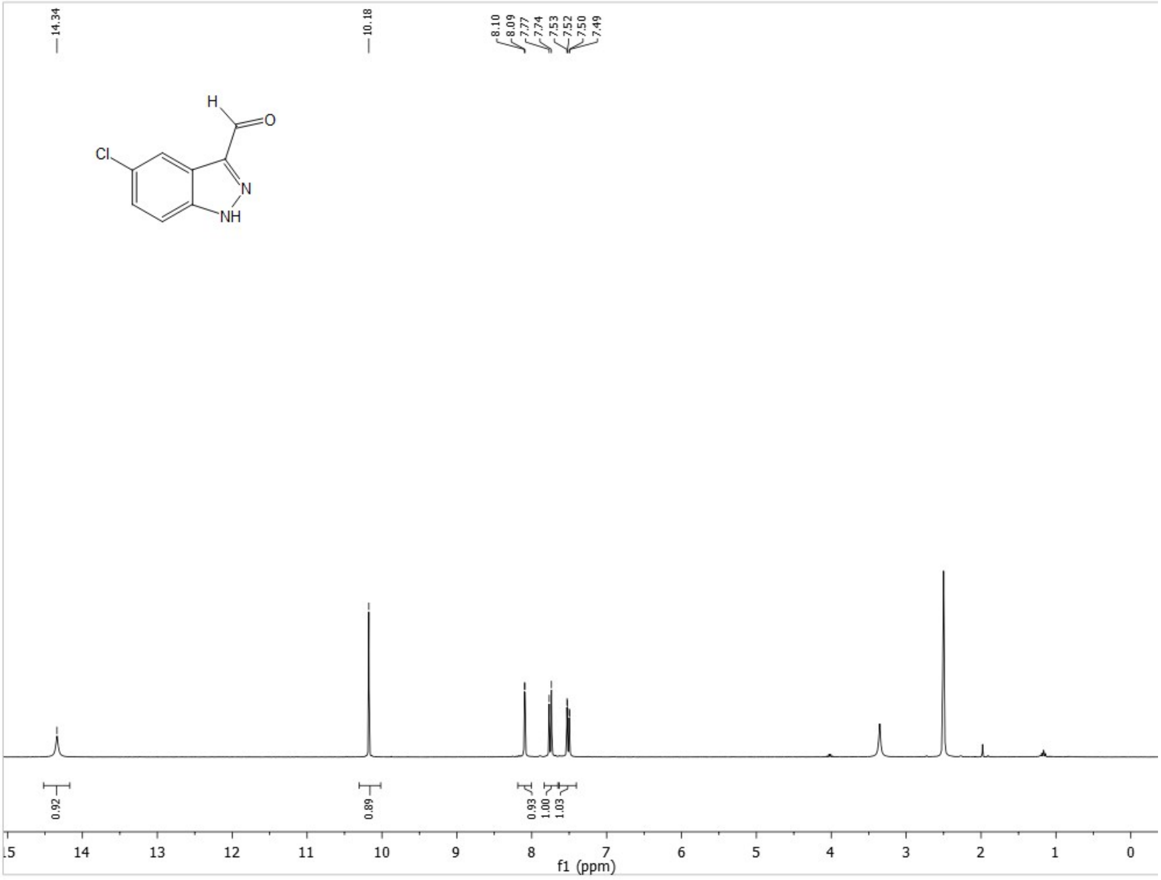

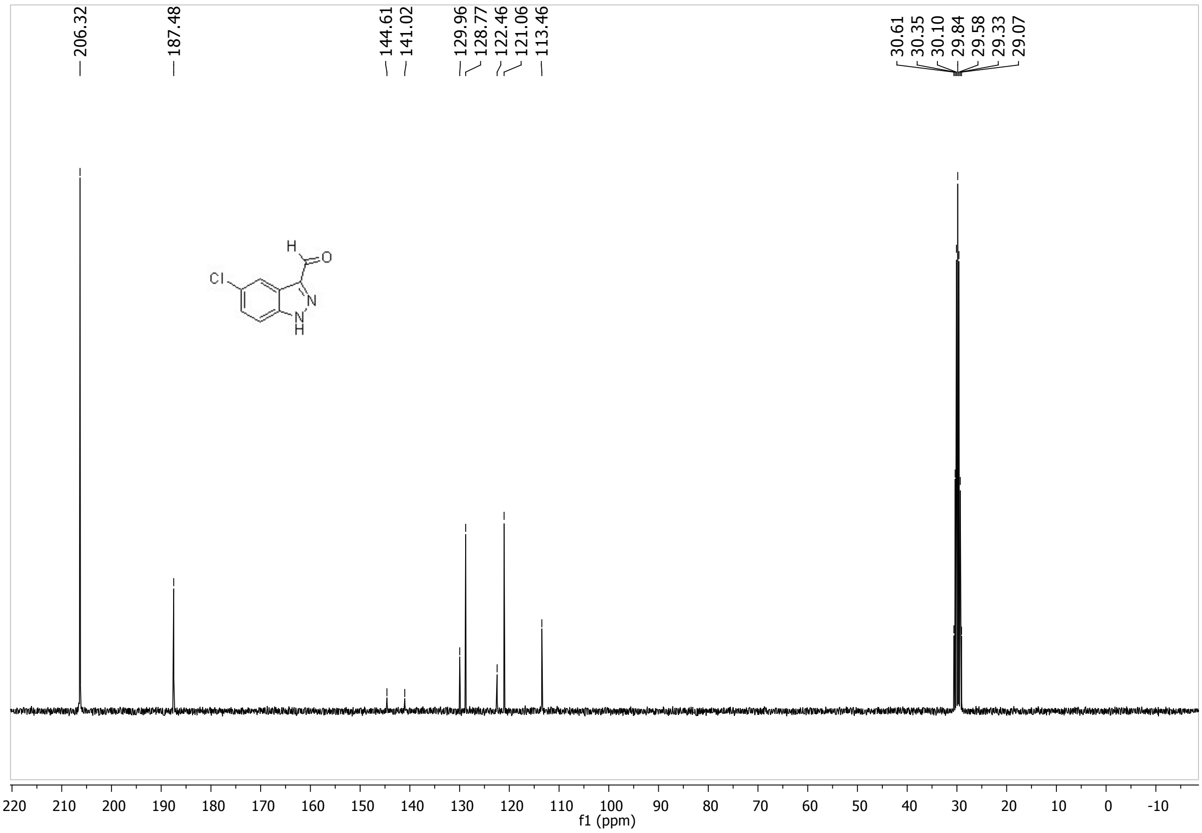

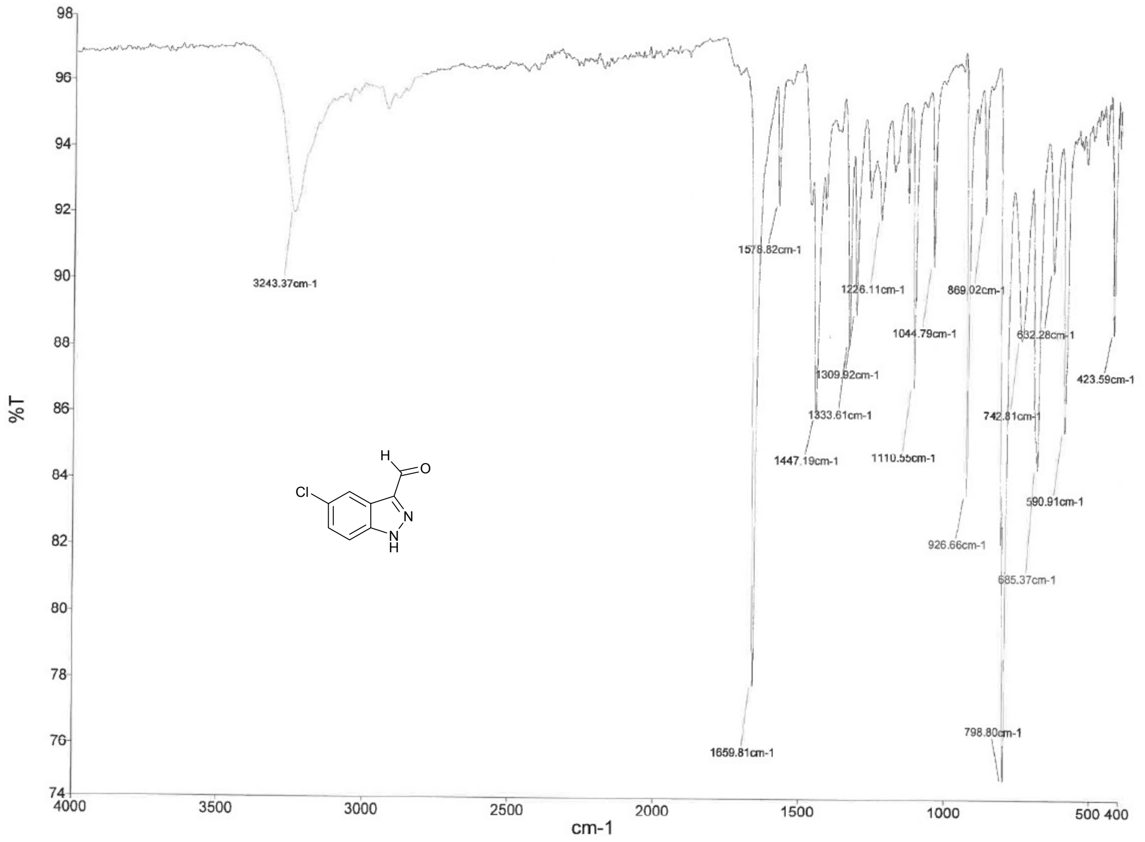
98
96
94
92
90
88
86
84
82
80
78
76
74
%T
4000
3500
3000
2500
2000
1500
1000
500 400
cm-1
3243.37cm-1
1578.82cm-1
1226.11cm-1
869.02cm-1
1044.79cm-1
632.28cm-1
1309.92cm-1
423.59cm-1
1333.61cm-1
742.81cm-1
1447.19cm-1
1110.55cm-1
590.91cm-1
926.66cm-1
685.37cm-1
798.80cm-1
1659.81cm-1
Cl
H
O
N
N
H

5-iodo-1*H*-indazole-3-carboxaldehyde (13b)

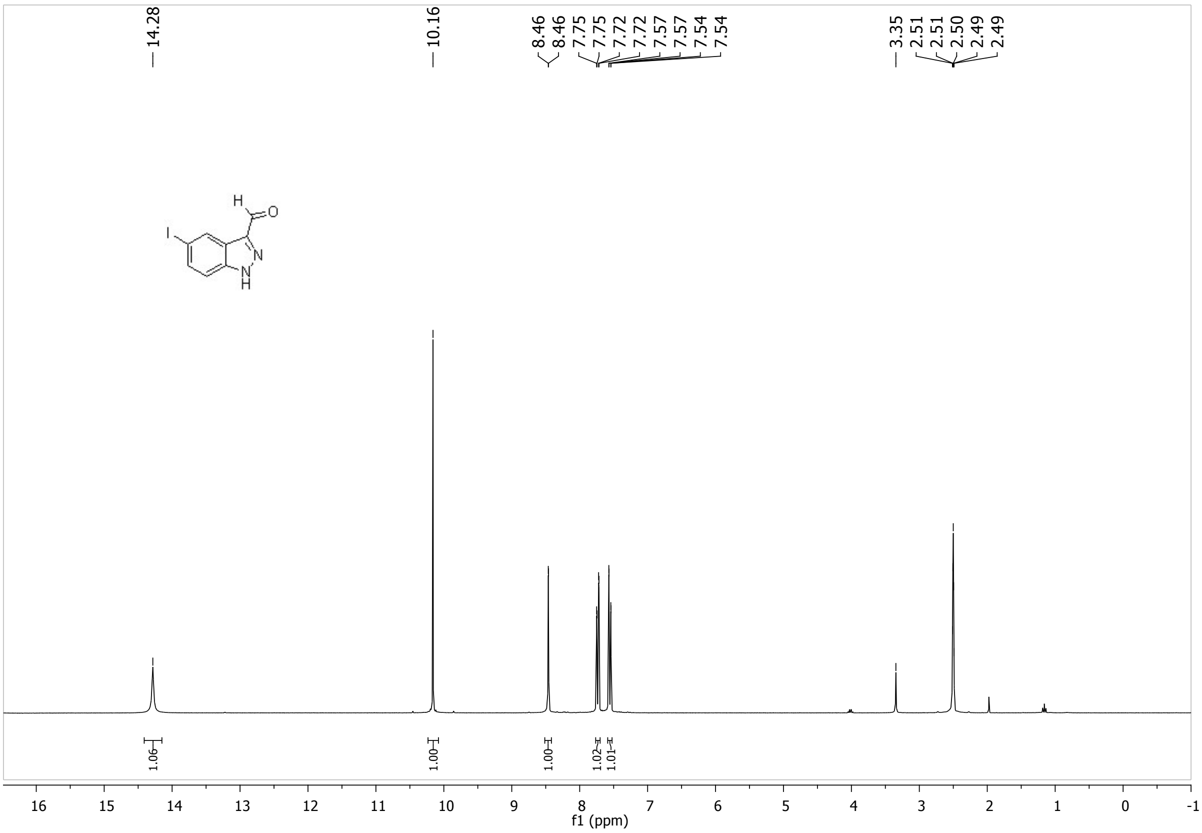

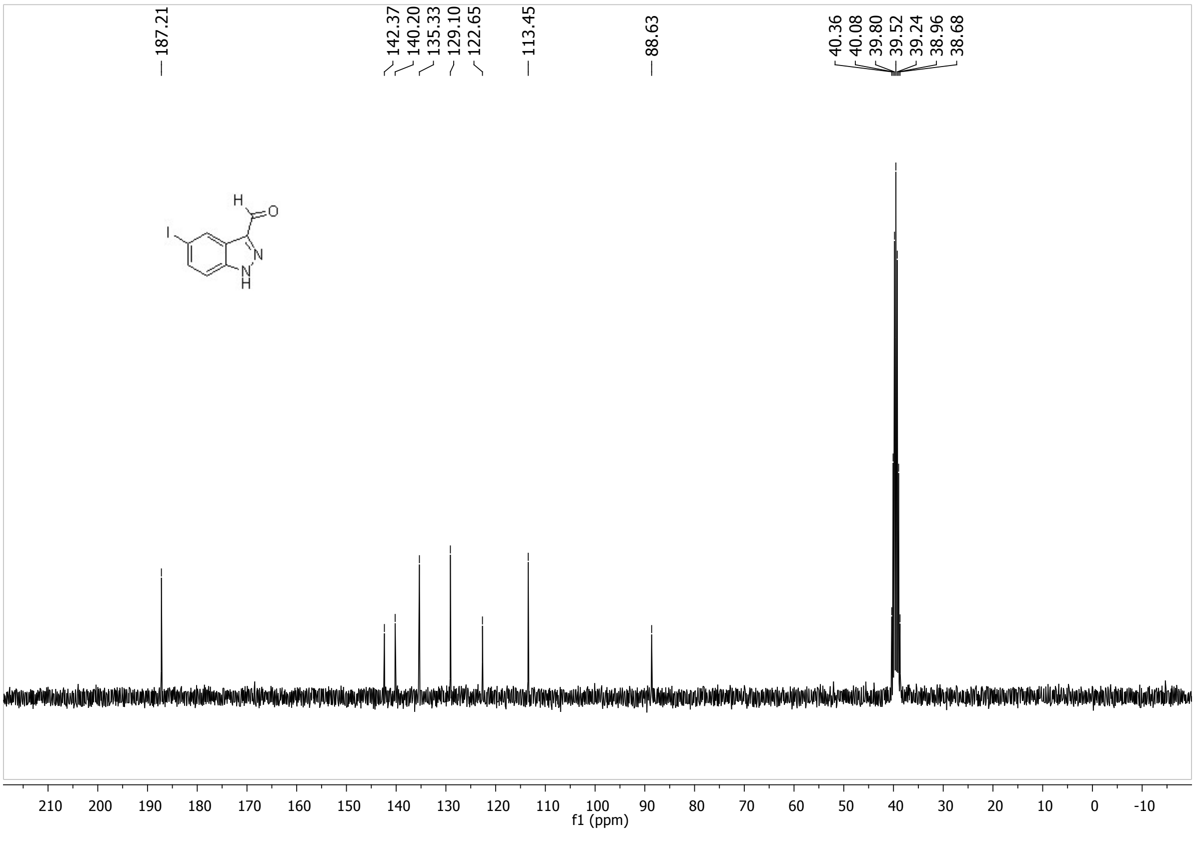

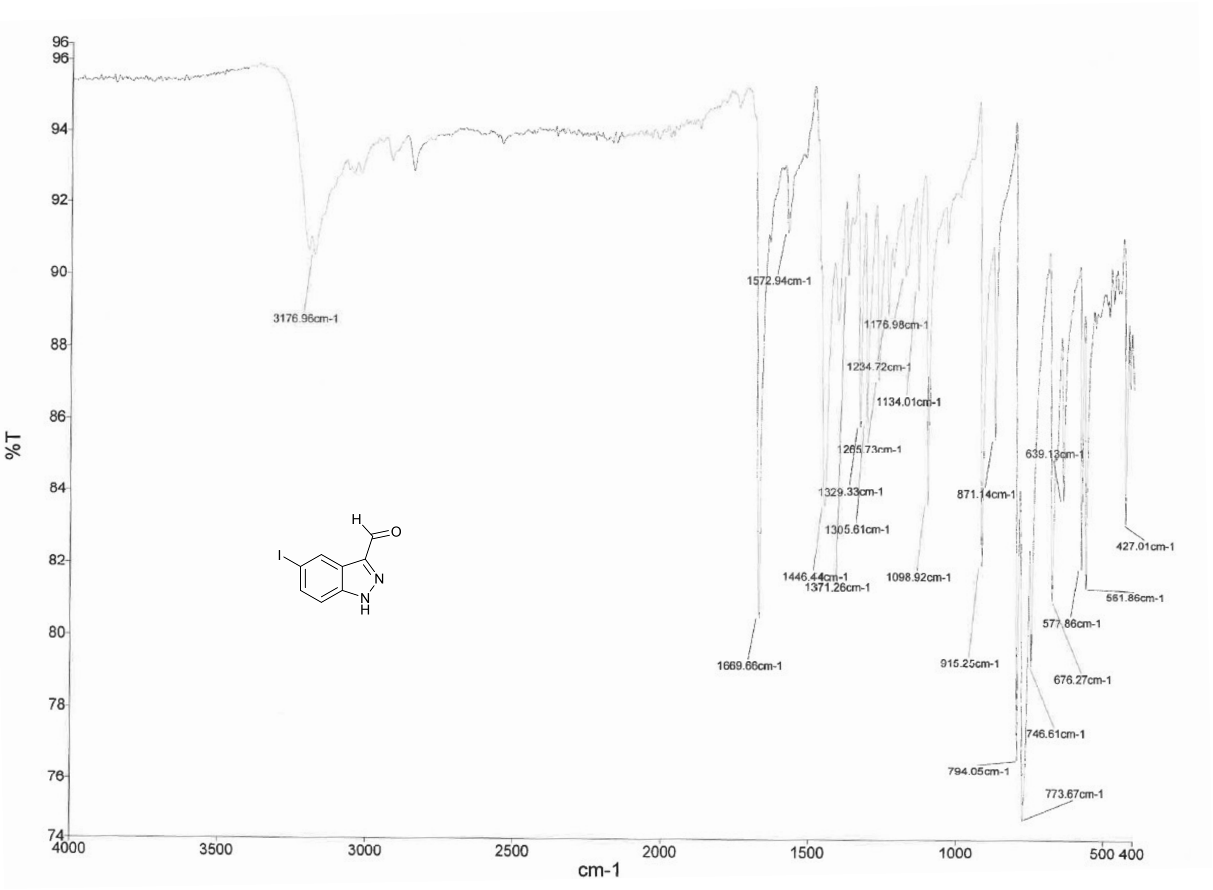
3176.96cm-1
1669.66cm-1
1572.94cm-1
1446.44cm-1
1371.26cm-1
1329.33cm-1
1305.61cm-1
1265.73cm-1
1234.72cm-1
1176.98cm-1
1134.01cm-1
1098.92cm-1
915.25cm-1
871.14cm-1
794.05cm-1
773.67cm-1
746.61cm-1
676.27cm-1
639.13cm-1
577.86cm-1
561.86cm-1
427.01cm-1
%T
cm-1

5-fluoro-1*H*-indazole-3-carboxaldehyde (14b)

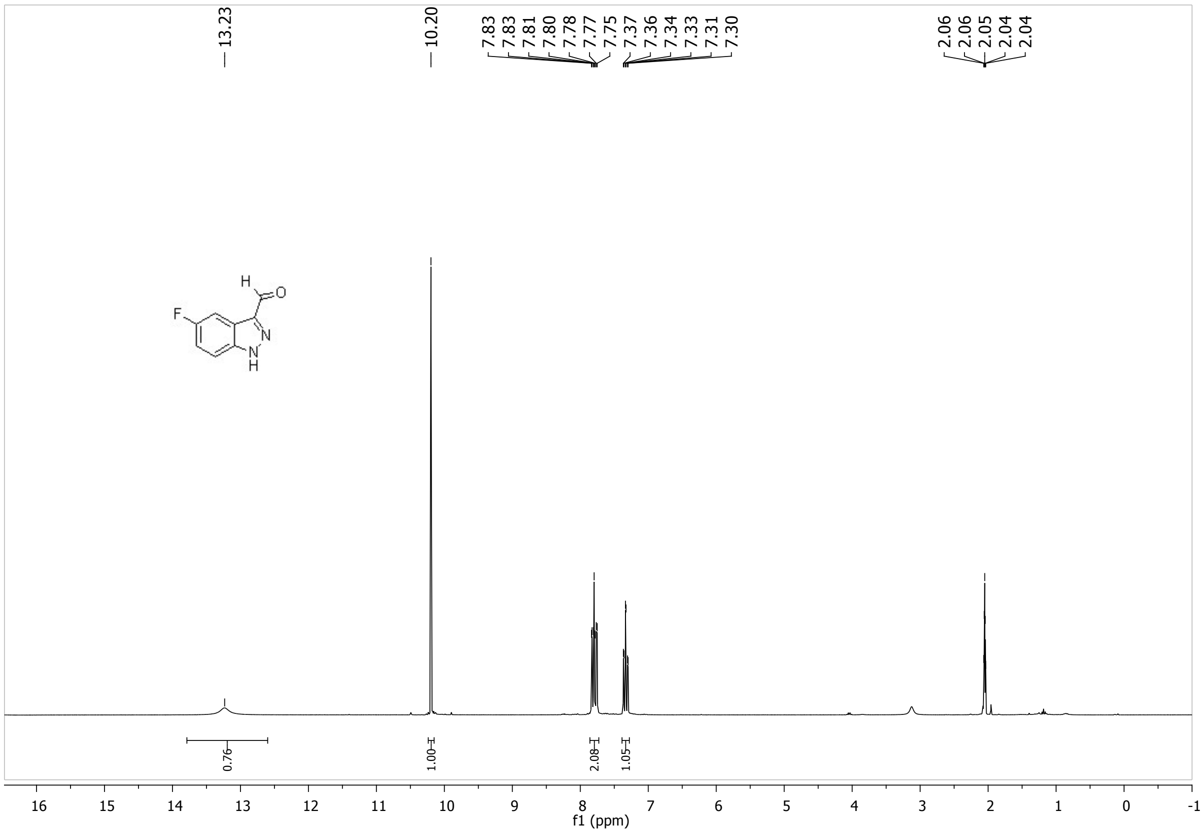

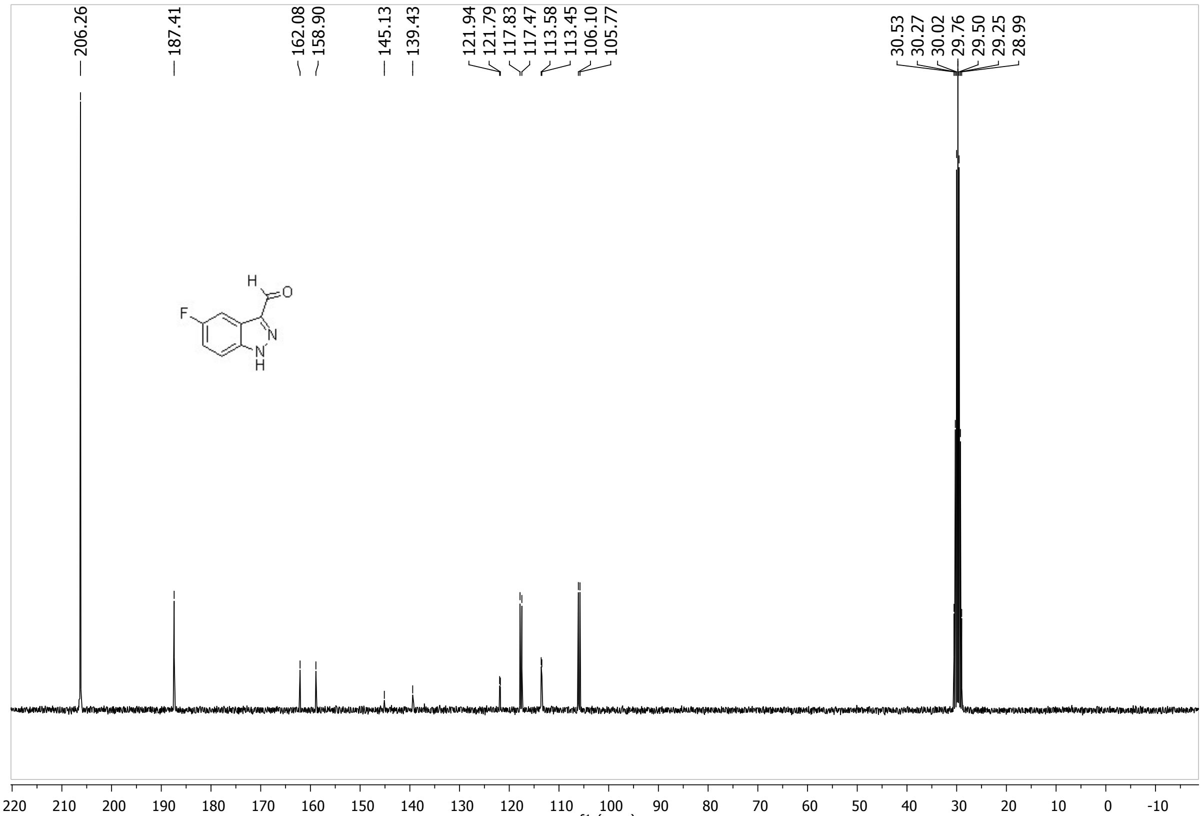

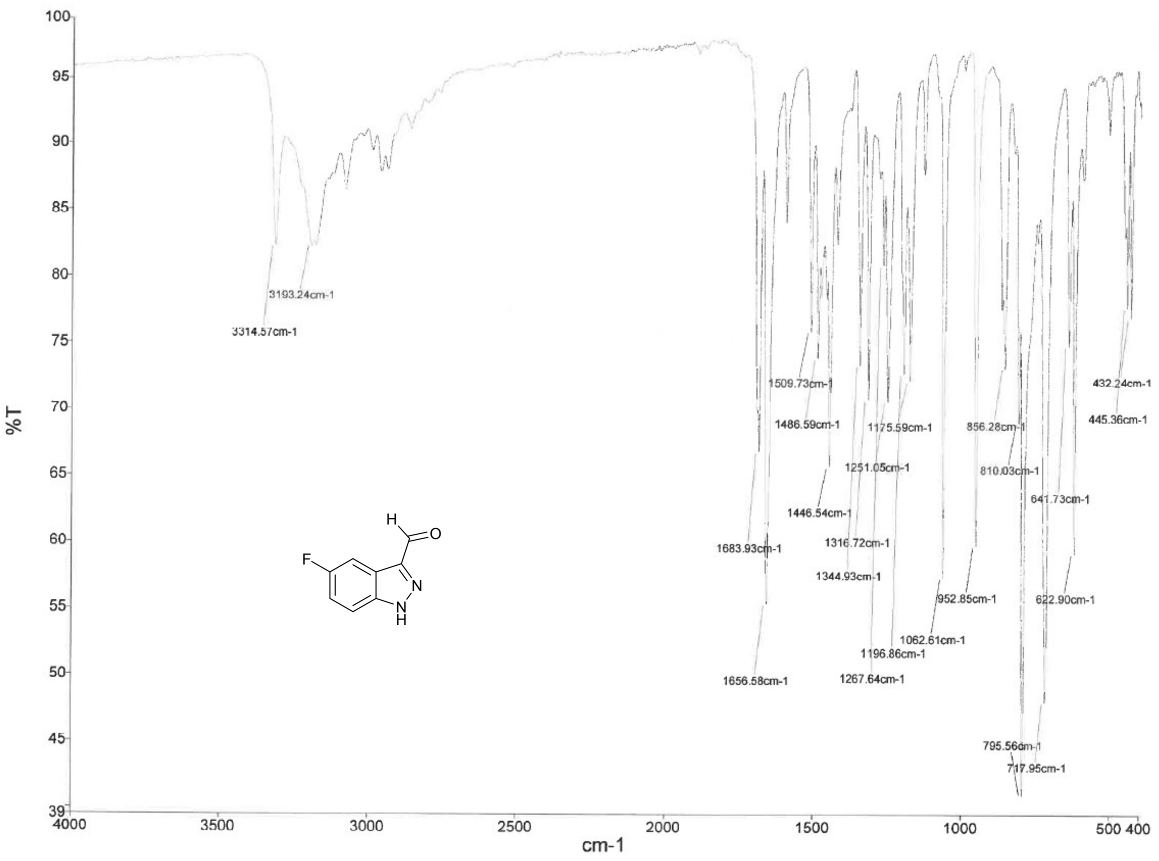

%T
cm-1
3314.57cm-1
3193.24cm-1
1683.93cm-1
1656.58cm-1
1509.73cm-1
1486.59cm-1
1446.54cm-1
1344.93cm-1
1316.72cm-1
1267.64cm-1
1251.05cm-1
1196.86cm-1
1175.59cm-1
1062.61cm-1
952.85cm-1
856.28cm-1
810.03cm-1
795.56cm-1
717.95cm-1
641.73cm-1
622.90cm-1
445.36cm-1
432.24cm-1

6-bromo-1*H*-Indazole-3-carboxaldehyde (15b)

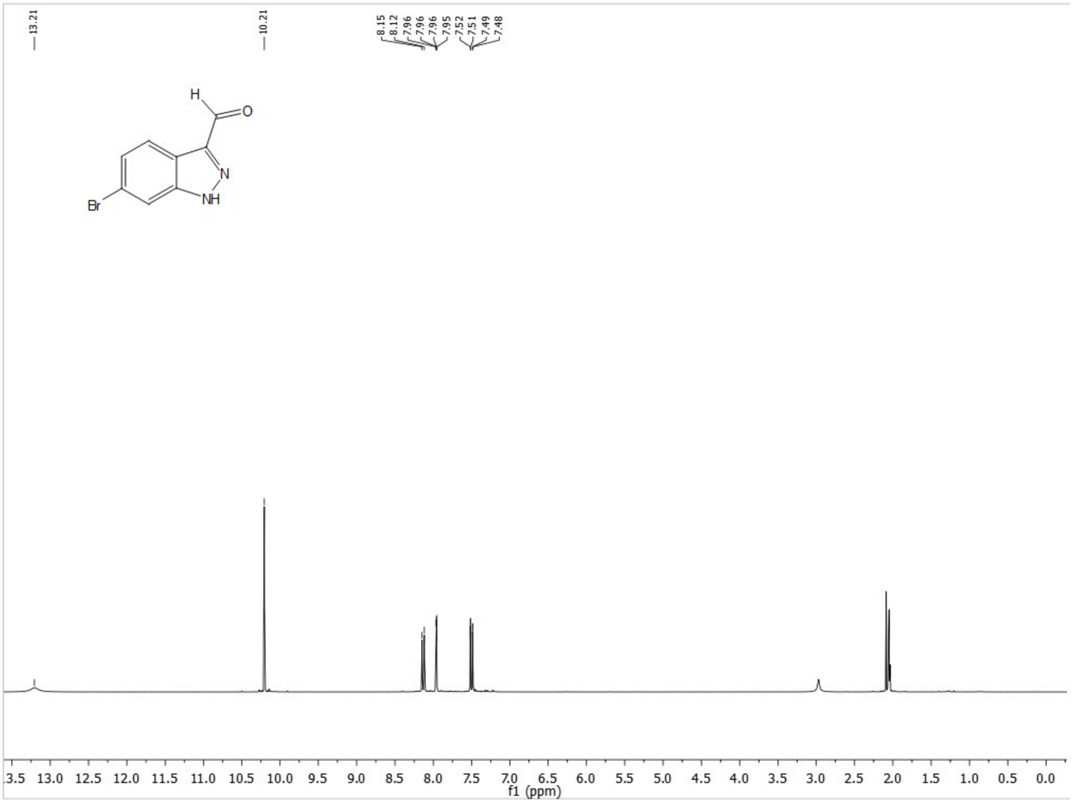

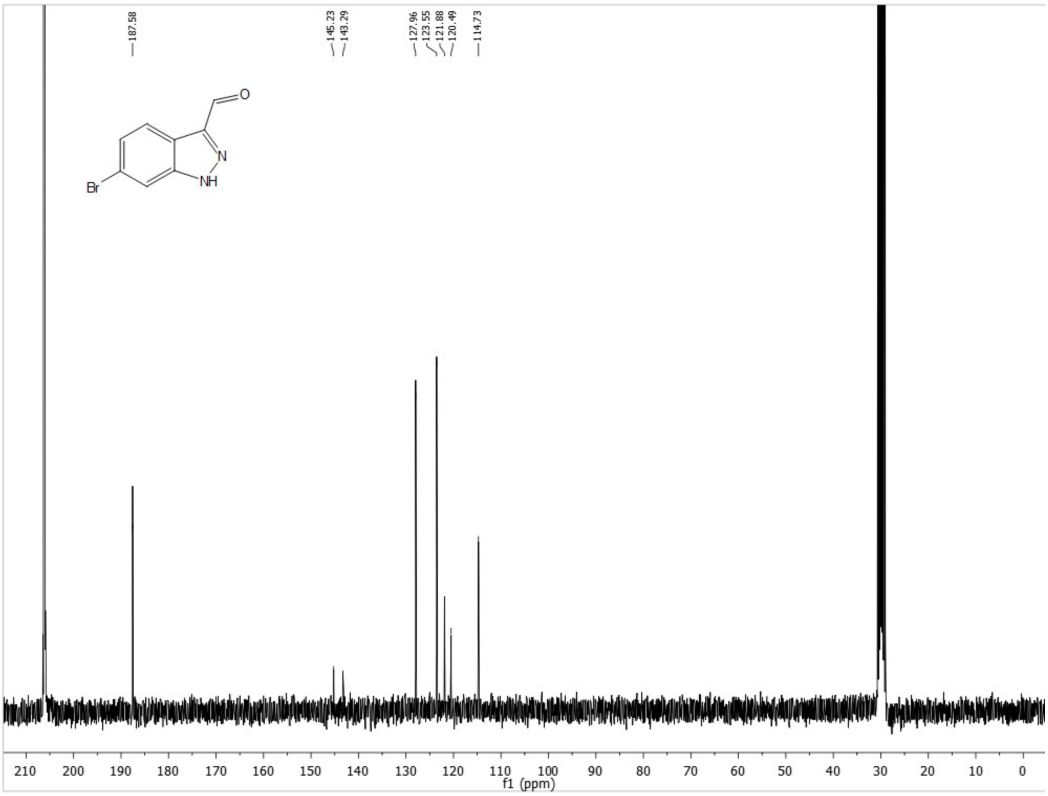

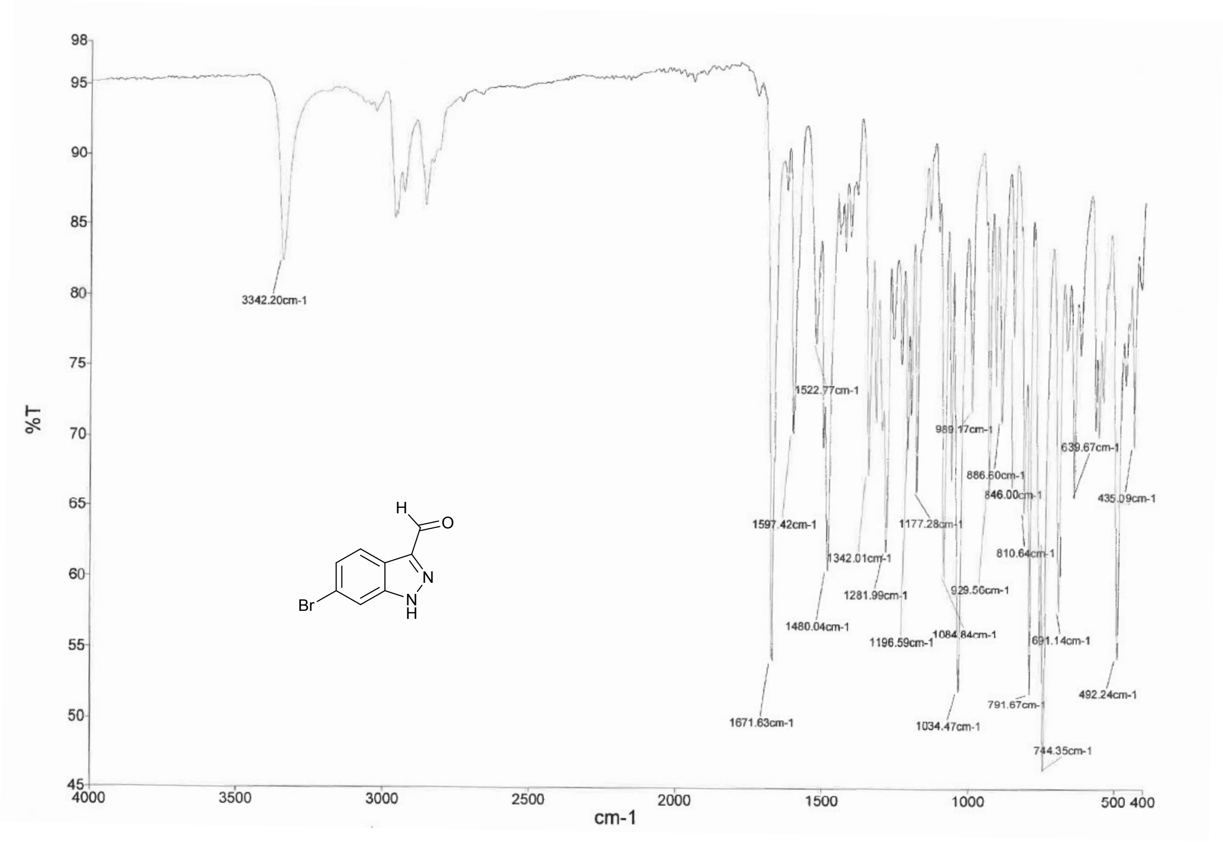
%T
cm-1
3342.20cm-1
1671.63cm-1
1597.42cm-1
1522.77cm-1
1480.04cm-1
1342.01cm-1
1281.99cm-1
1196.59cm-1
1177.28cm-1
1084.84cm-1
1034.47cm-1
989.17cm-1
929.56cm-1
886.60cm-1
846.00cm-1
810.64cm-1
791.67cm-1
744.35cm-1
691.14cm-1
639.67cm-1
492.24cm-1
435.09cm-1
Br
H
O
N
H

6-fluoro-1*H*-indazole-3-carboxaldehyde (16b).

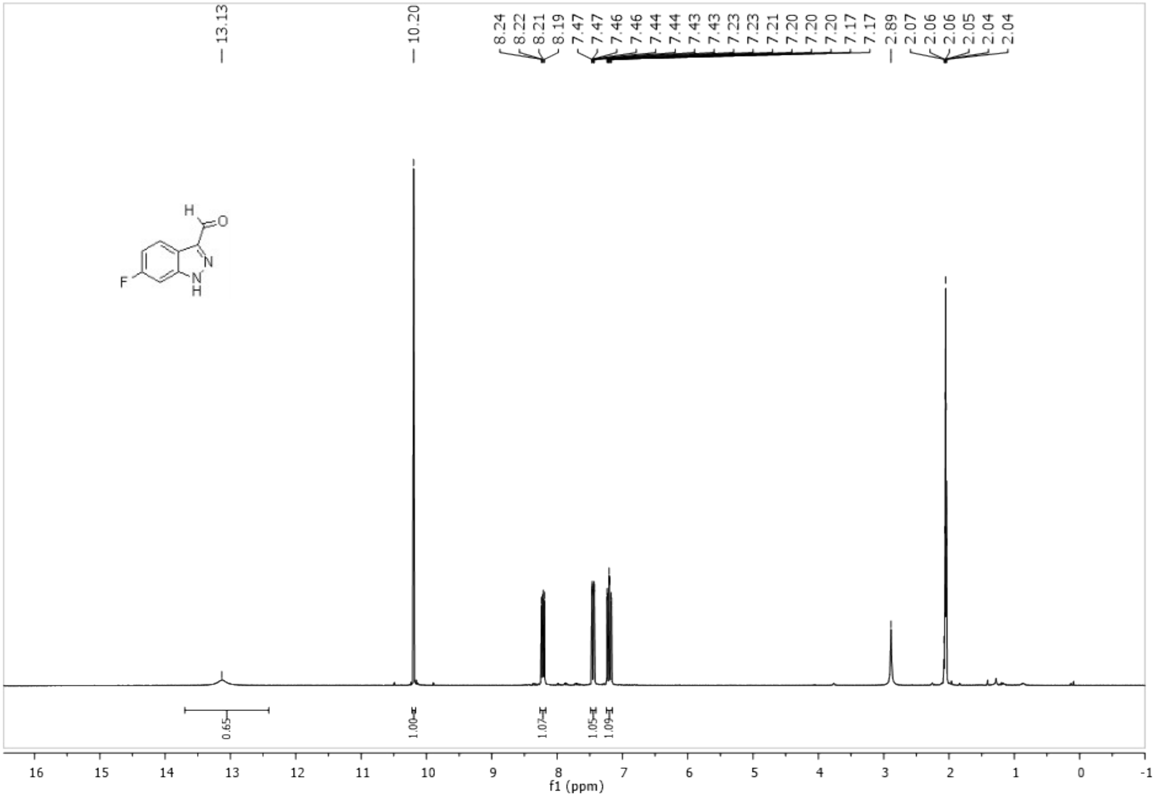

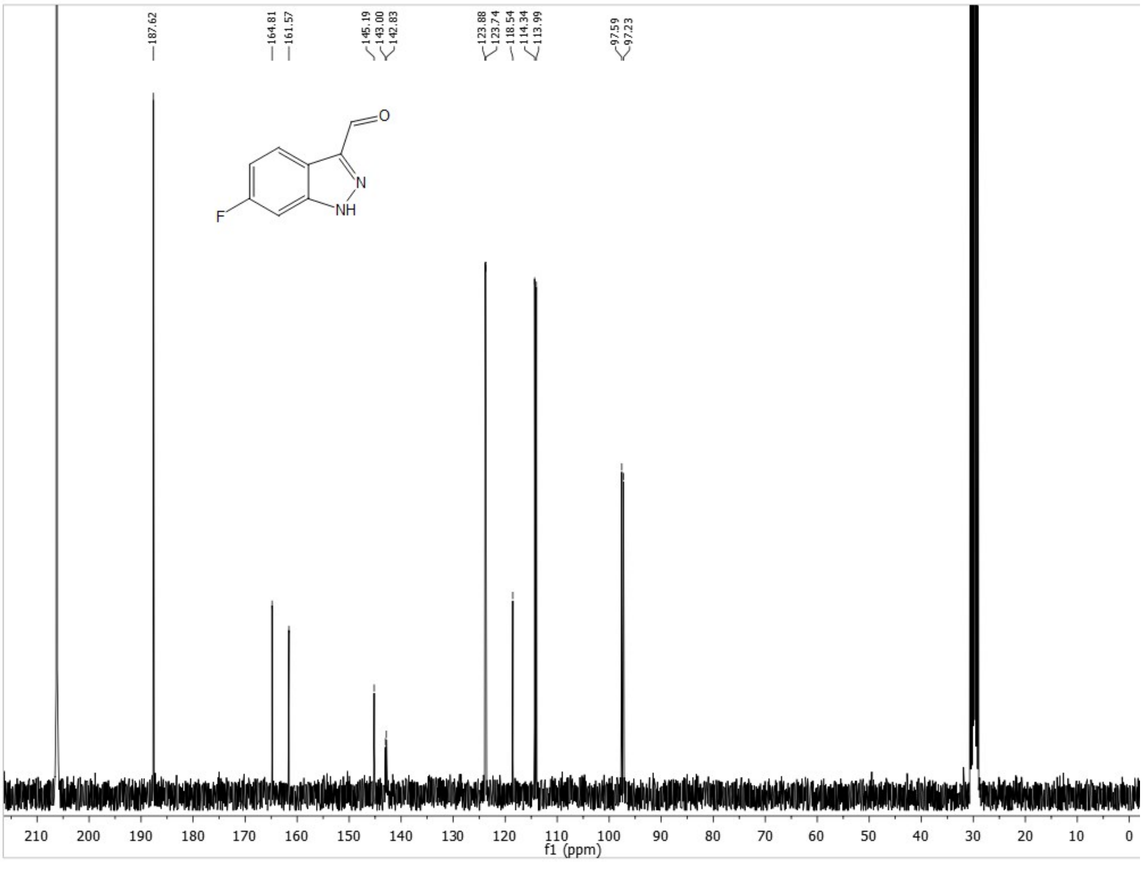

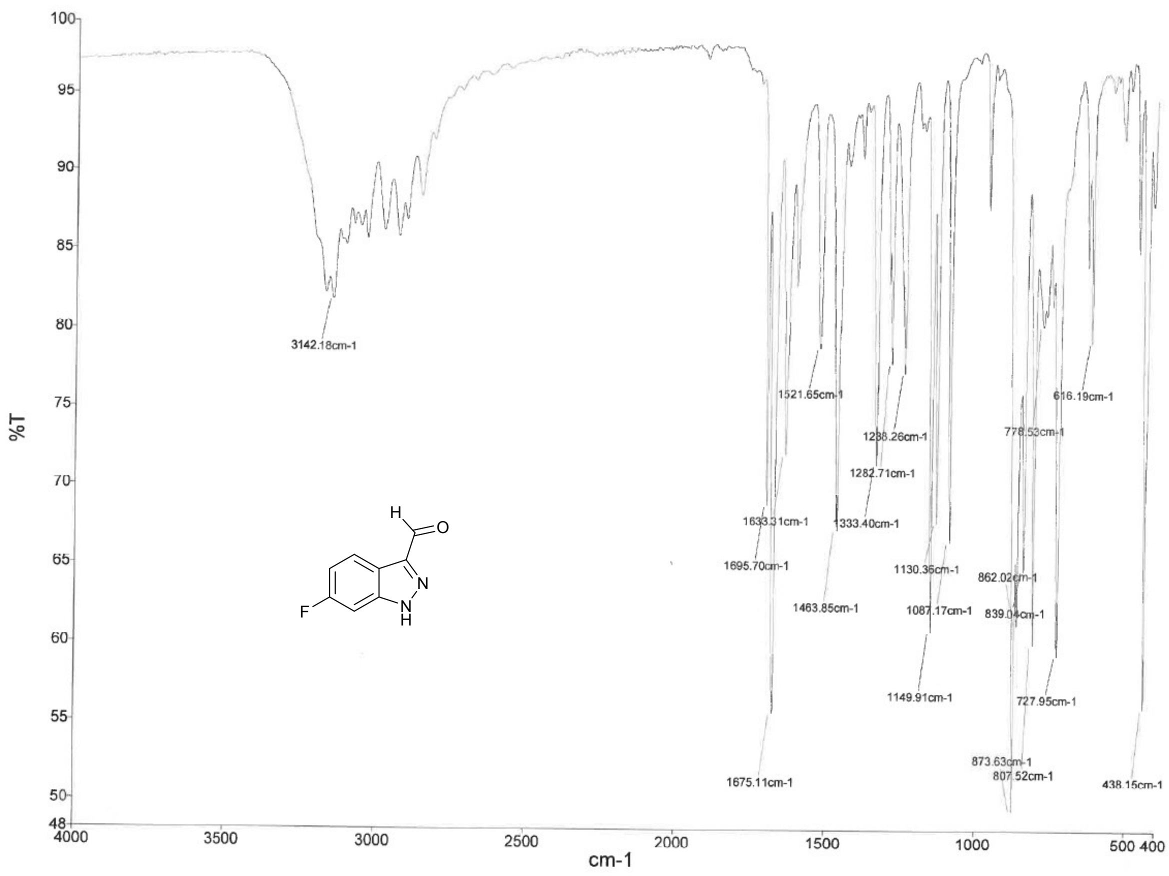

%T
cm-1
3142.18cm-1
1521.65cm-1
1288.26cm-1
1282.71cm-1
1633.31cm-1
1333.40cm-1
1695.70cm-1
1463.85cm-1
1130.36cm-1
1087.17cm-1
1149.91cm-1
1675.11cm-1
862.02cm-1
839.04cm-1
778.53cm-1
727.95cm-1
873.63cm-1
807.52cm-1
616.19cm-1
438.15cm-1

5-Methoxy-1*H*-Indazole-3-carboxaldehyde (17b)

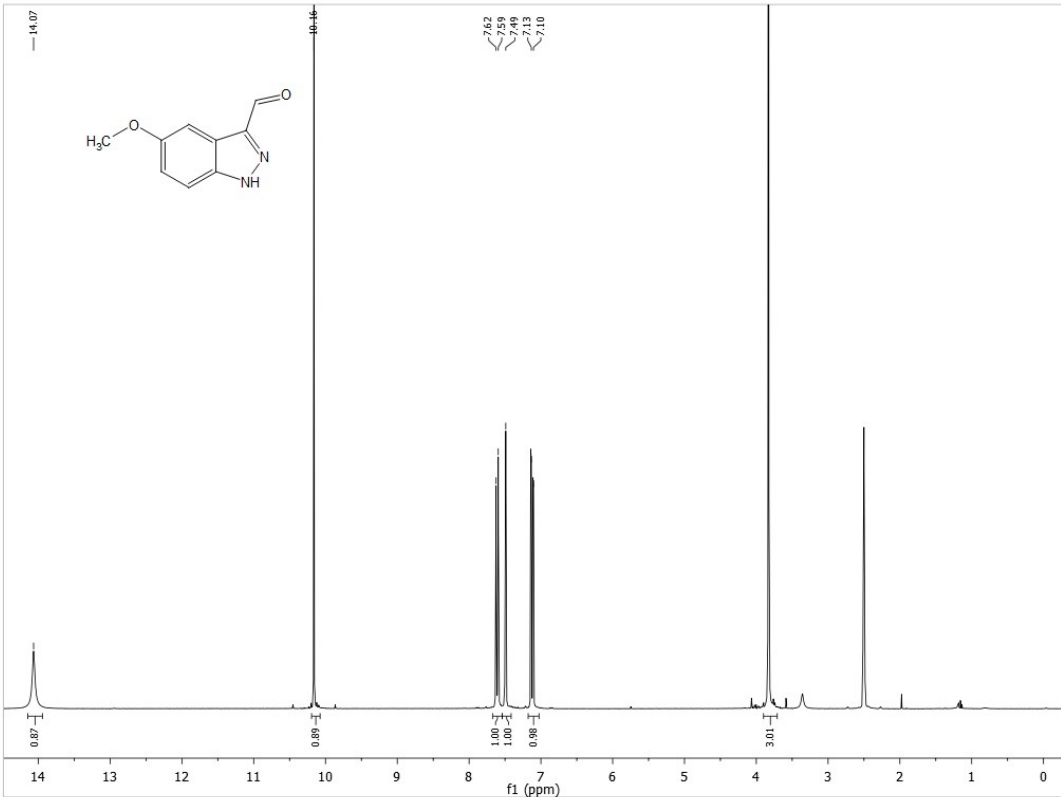

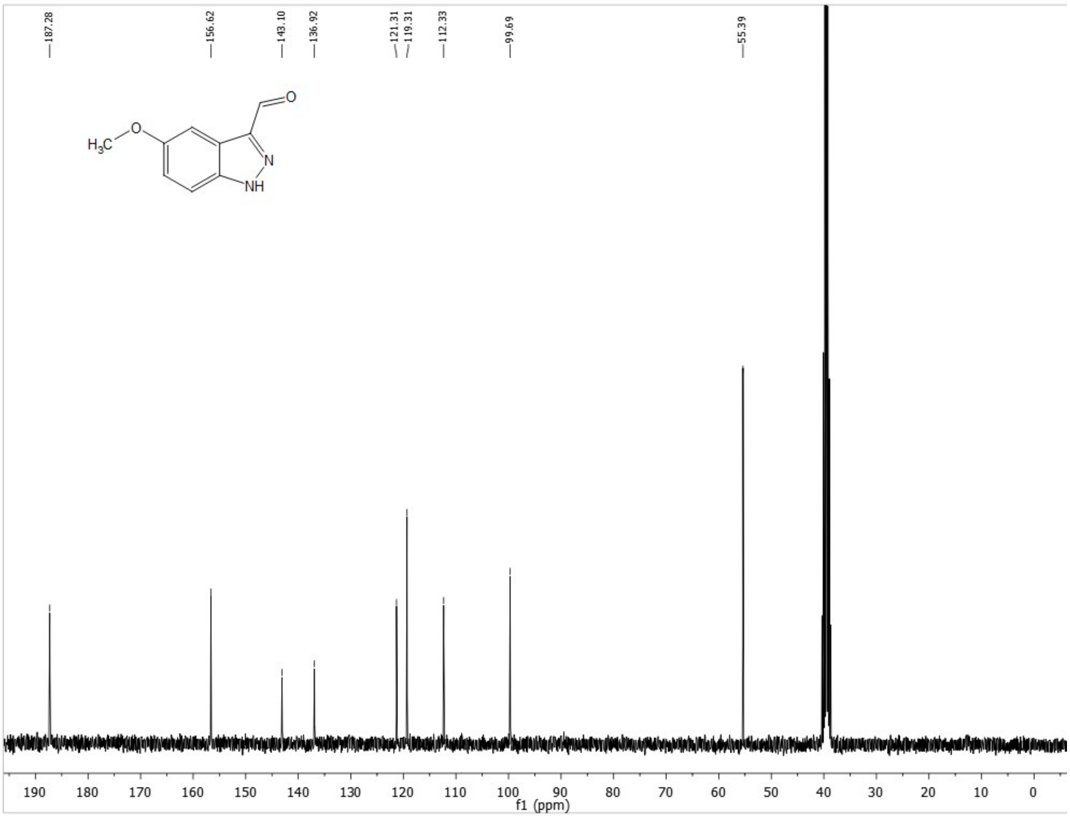

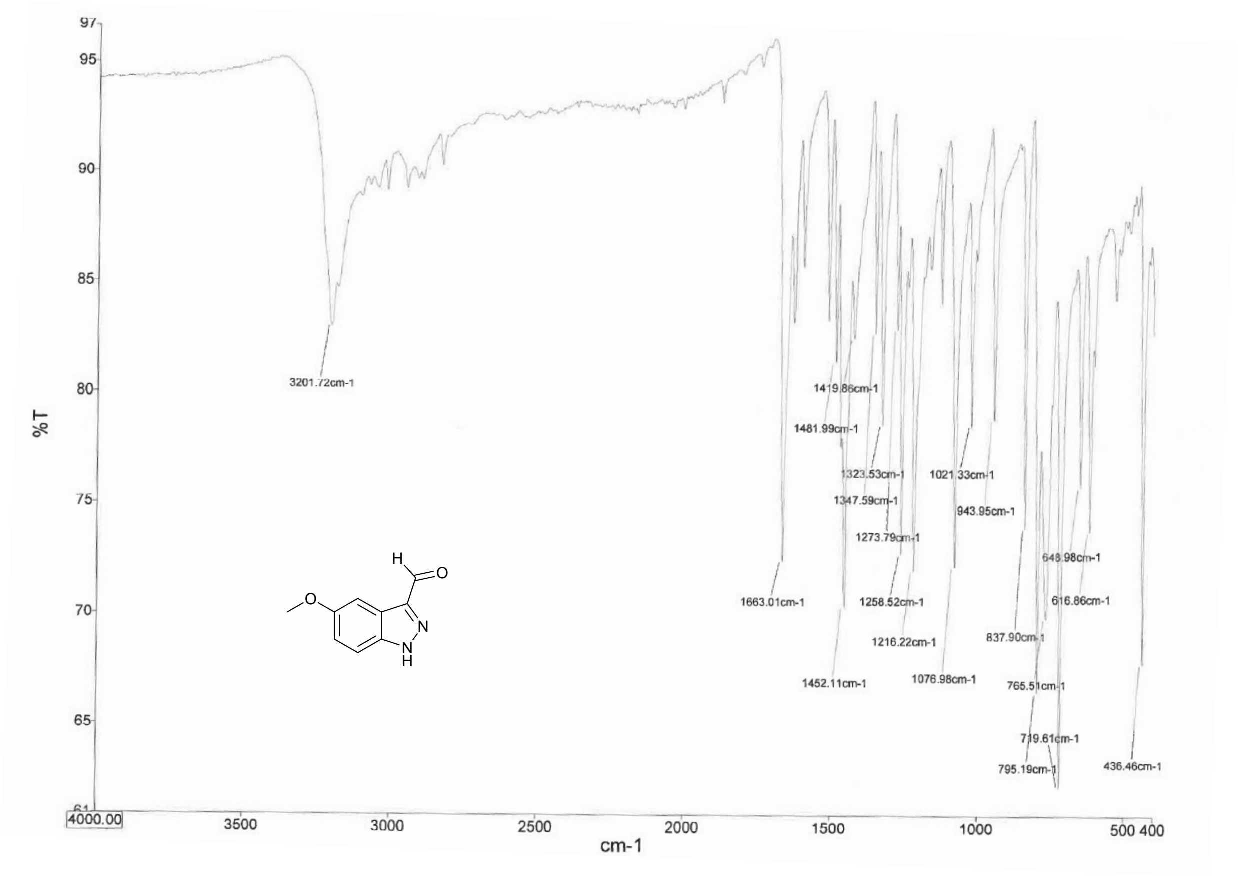
%T
cm-1
4000.00
3500
3000
2500
2000
1500
1000
500 400
3201.72cm-1
1663.01cm-1
1419.86cm-1
1481.99cm-1
1323.53cm-1
1347.59cm-1
1273.79cm-1
1258.52cm-1
1216.22cm-1
1452.11cm-1
1076.98cm-1
1021.33cm-1
943.95cm-1
837.90cm-1
765.51cm-1
719.61cm-1
795.19cm-1
648.98cm-1
616.86cm-1
436.46cm-1

5-Benzyloxy-1*H*-indazole-3-carboxaldehyde (18b)

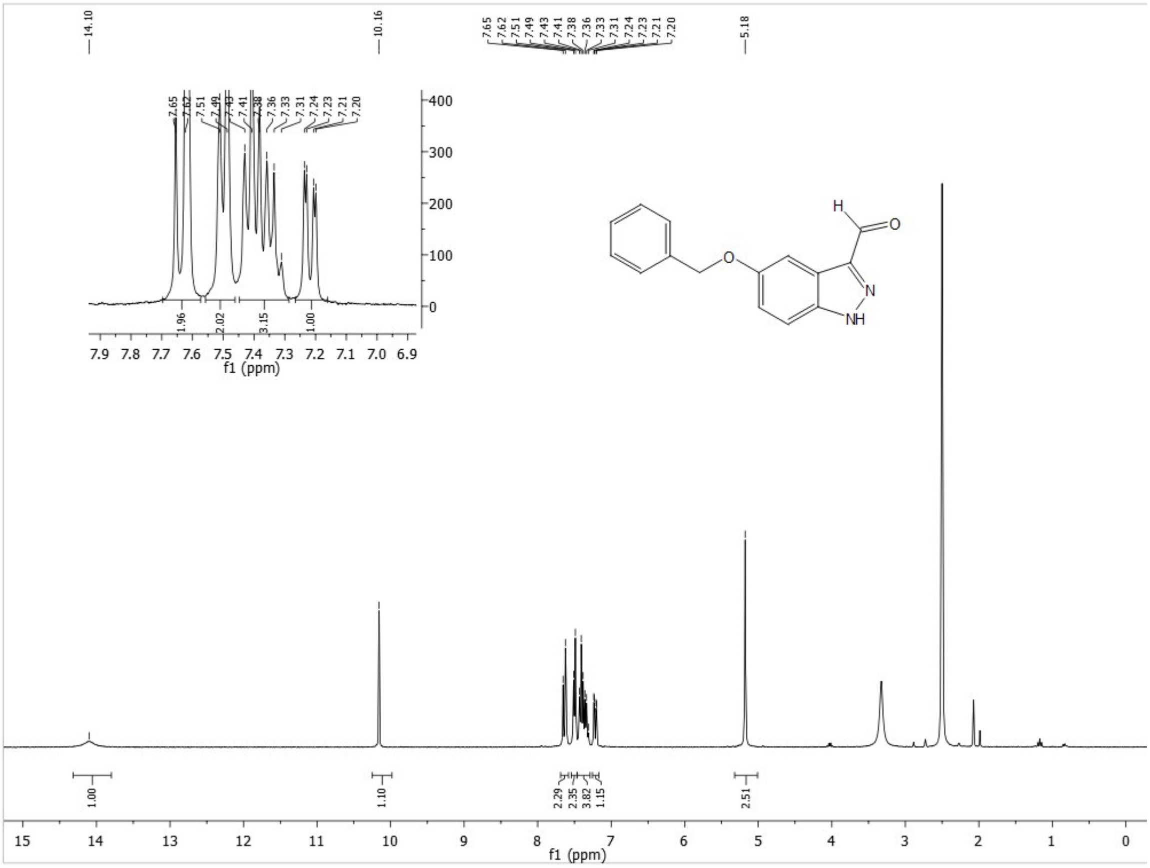

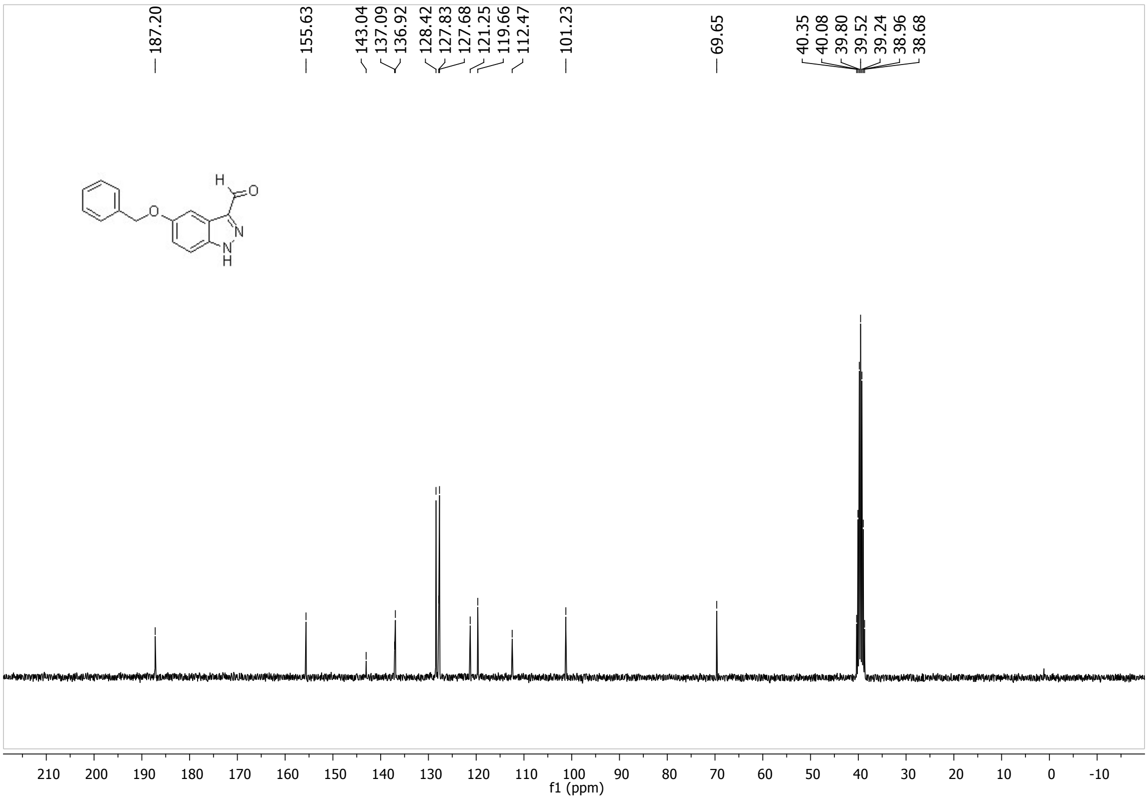

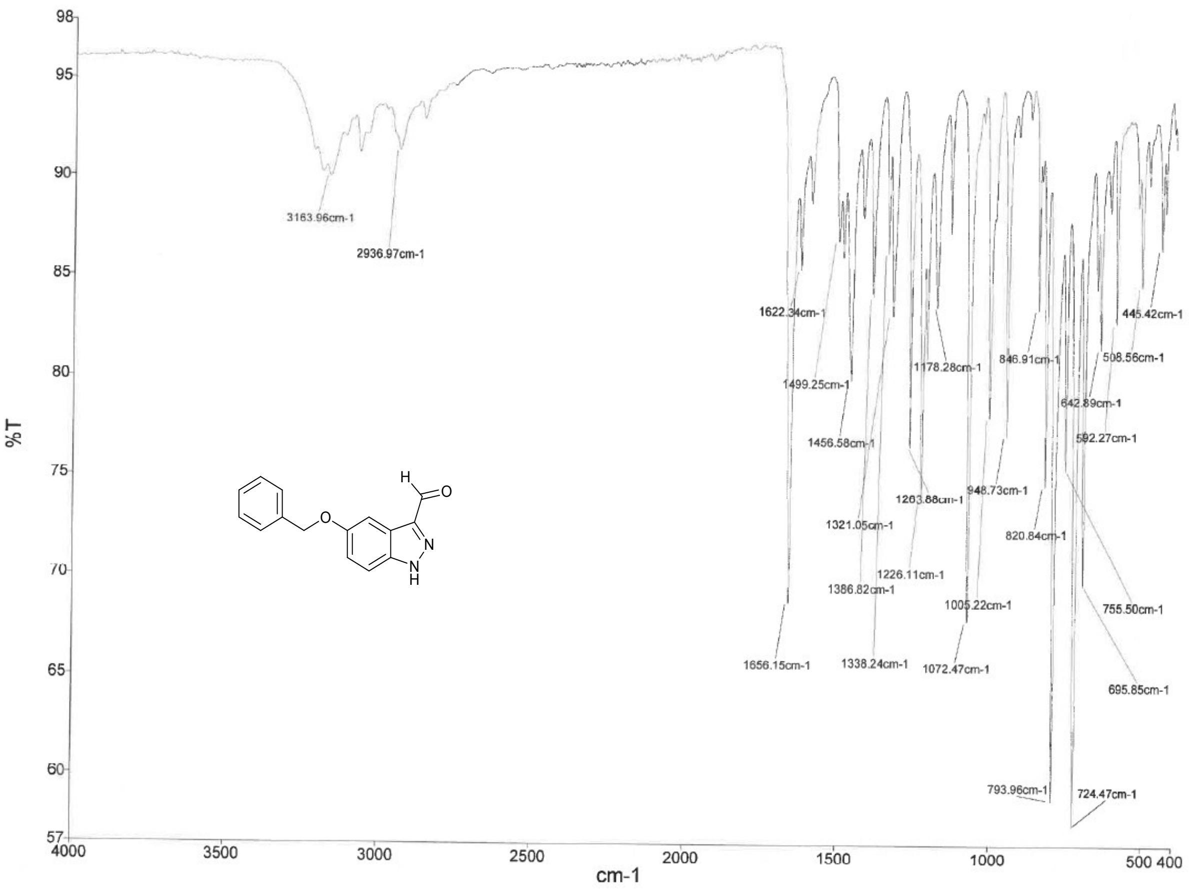
98
95
90
85
80
75
70
65
60
57
%T
4000
3500
3000
2500
2000
1500
1000
500 400
cm-1
3163.96cm-1
2936.97cm-1
1622.34cm-1
1499.25cm-1
1456.58cm-1
1321.05cm-1
1386.82cm-1
1656.15cm-1
1338.24cm-1
1226.11cm-1
1263.88cm-1
1178.28cm-1
1072.47cm-1
1005.22cm-1
948.73cm-1
846.91cm-1
820.84cm-1
793.96cm-1
755.50cm-1
724.47cm-1
695.85cm-1
642.89cm-1
592.27cm-1
508.56cm-1
445.42cm-1
H
O
N
H

1-(1*H*-indazol-3-yl)ethanone (19b)

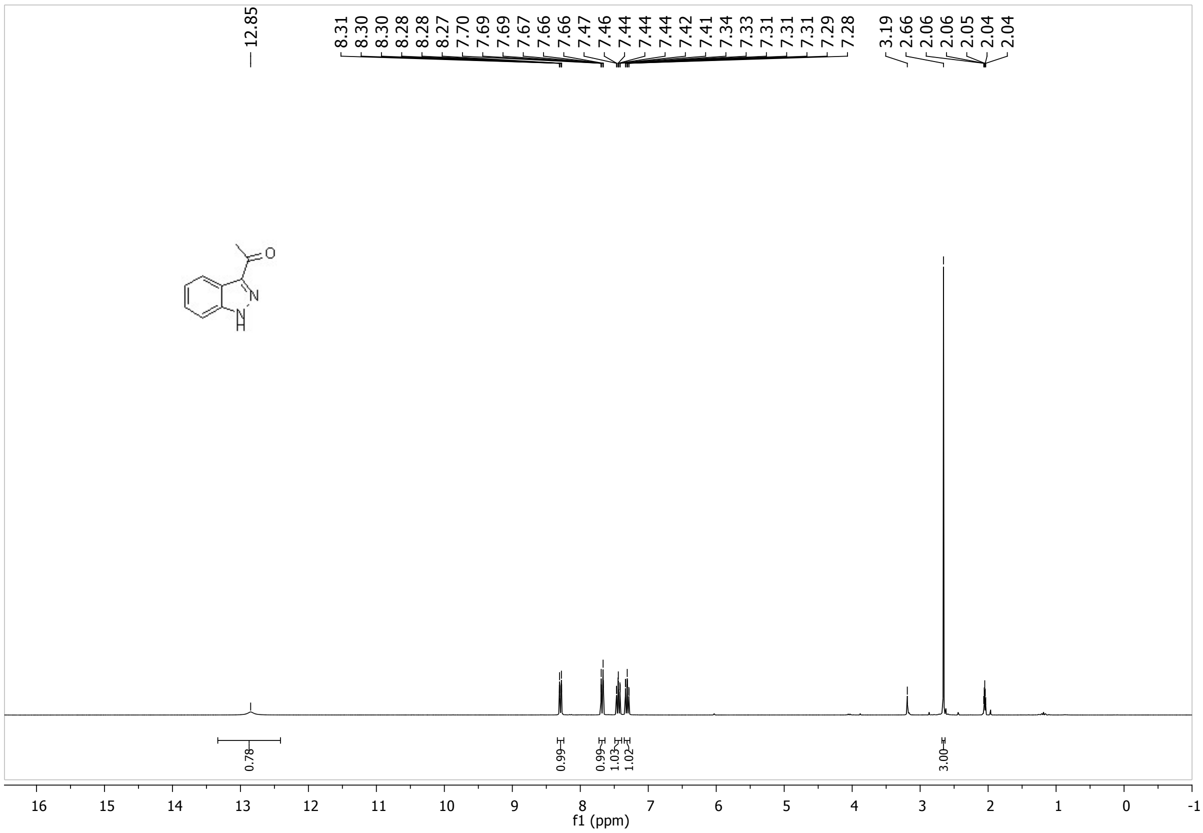

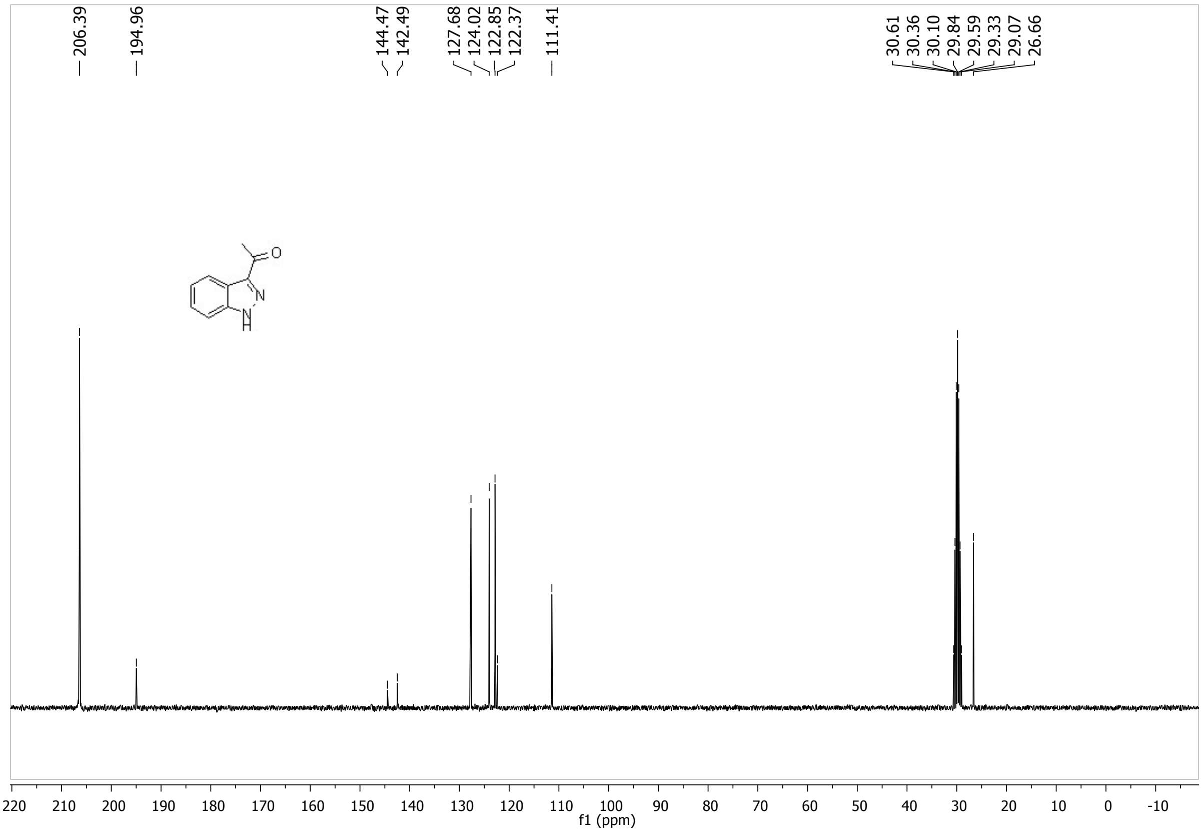

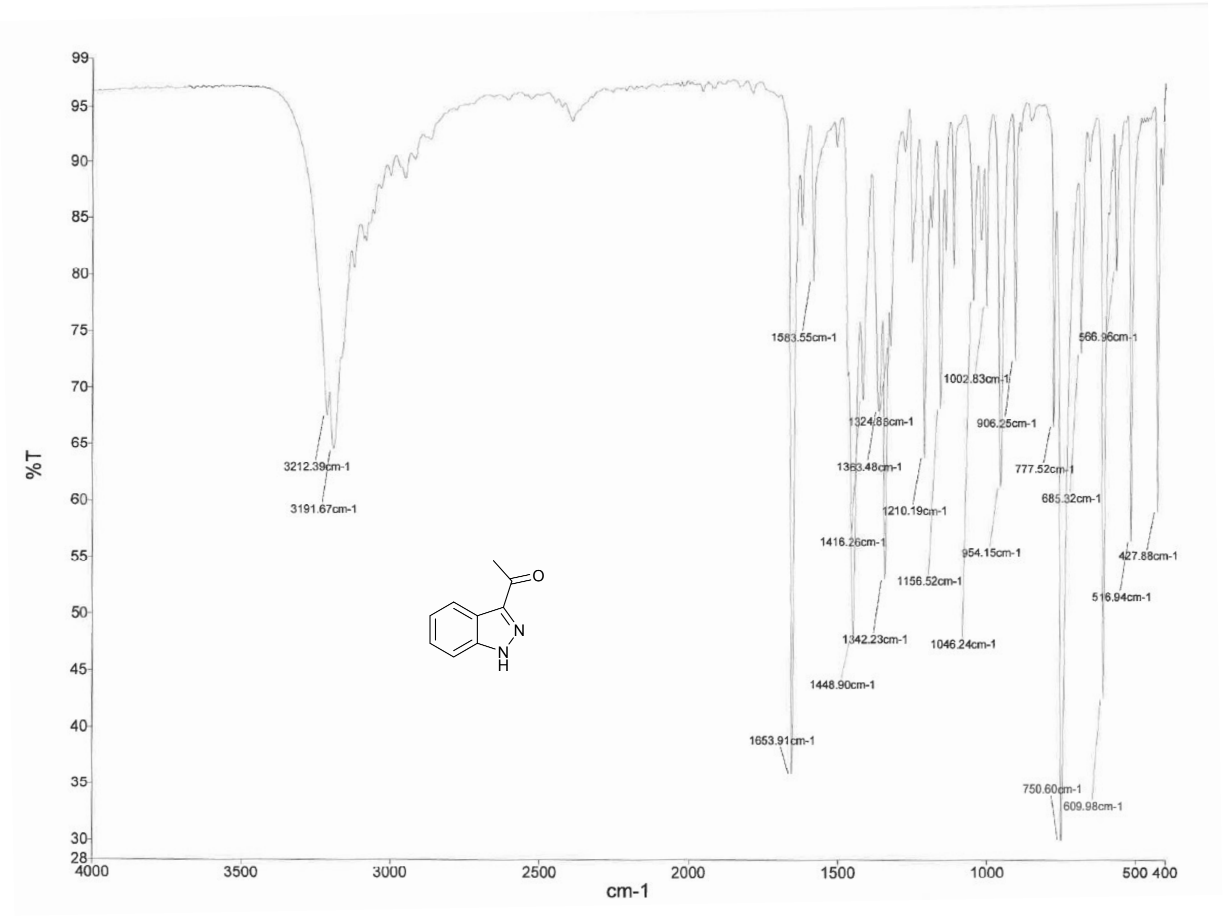
99
95
90
85
80
75
70
65
60
55
50
45
40
35
30
28
%T
4000
3500
3000
2500
2000
1500
1000
500 400
cm-1
3212.39cm-1
3191.67cm-1
1583.55cm-1
1653.91cm-1
1448.90cm-1
1416.26cm-1
1383.48cm-1
1342.23cm-1
1324.86cm-1
1210.19cm-1
1156.52cm-1
1046.24cm-1
1002.83cm-1
954.15cm-1
906.25cm-1
777.52cm-1
750.60cm-1
685.32cm-1
609.98cm-1
566.96cm-1
516.94cm-1
427.88cm-1
O
N
H

7-methyl-1*H*-indazole-3-carboxaldehyde (20b)

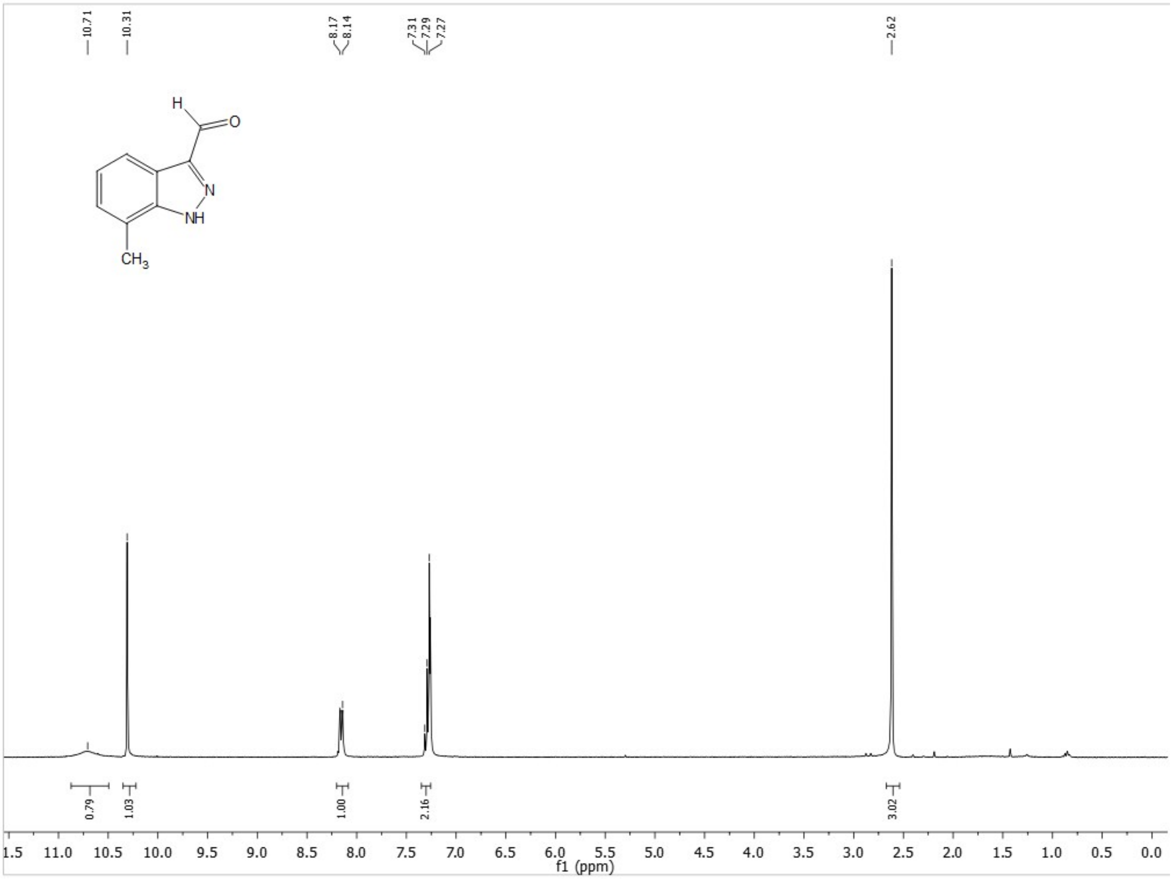

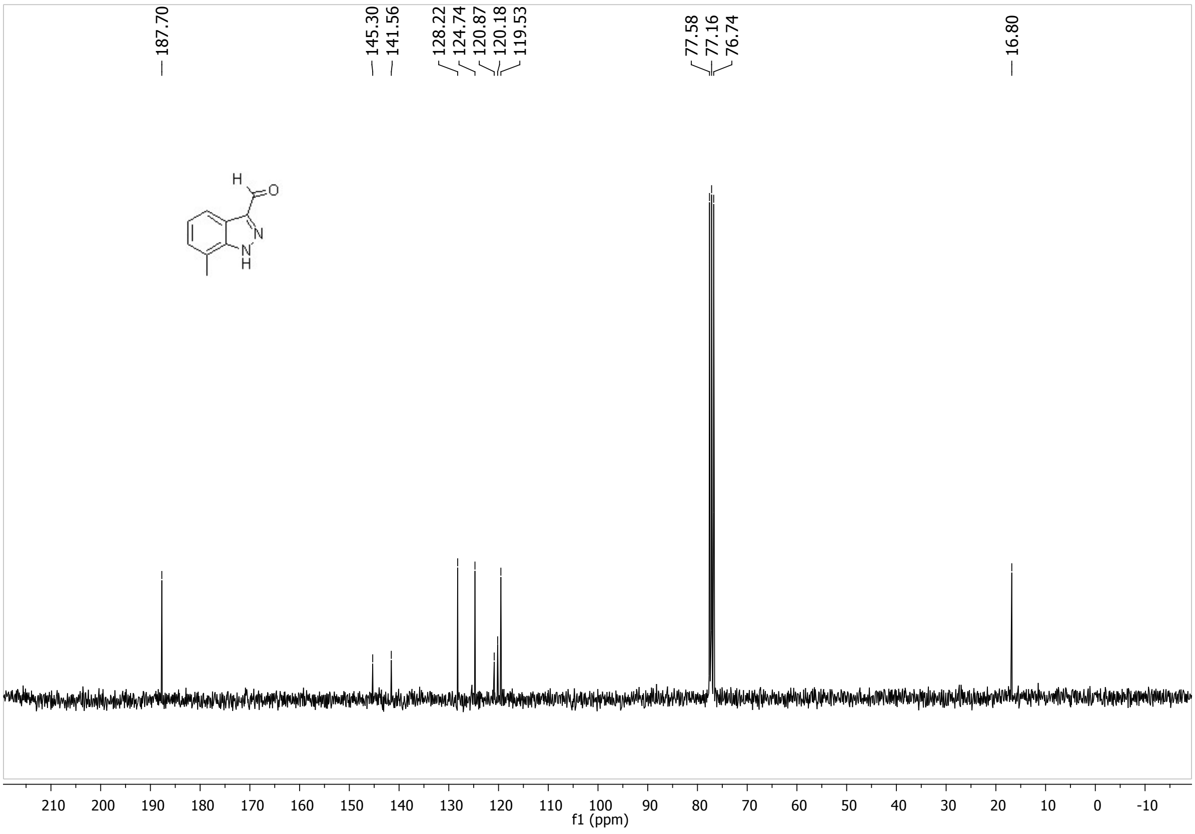

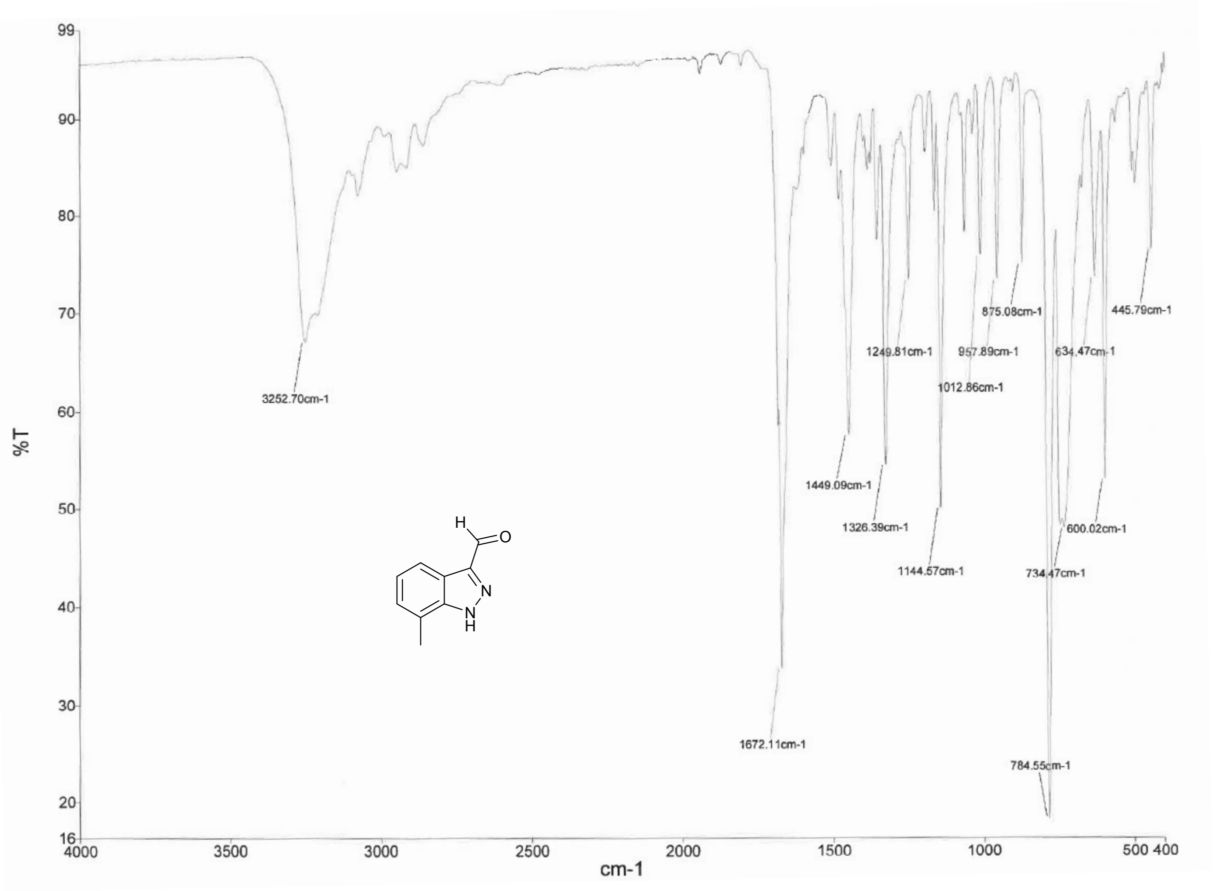
99
90
80
70
60
50
40
30
20
16
%T
4000
3500
3000
2500
2000
1500
1000
500 400
cm-1
3252.70cm-1
1672.11cm-1
1449.09cm-1
1326.39cm-1
1249.81cm-1
1144.57cm-1
1012.86cm-1
957.89cm-1
875.08cm-1
784.55cm-1
734.47cm-1
634.47cm-1
600.02cm-1
445.79cm-1
H
O
N
H

5-carboxy-1*H*-Indazole-3-carboxaldehyde (21b)

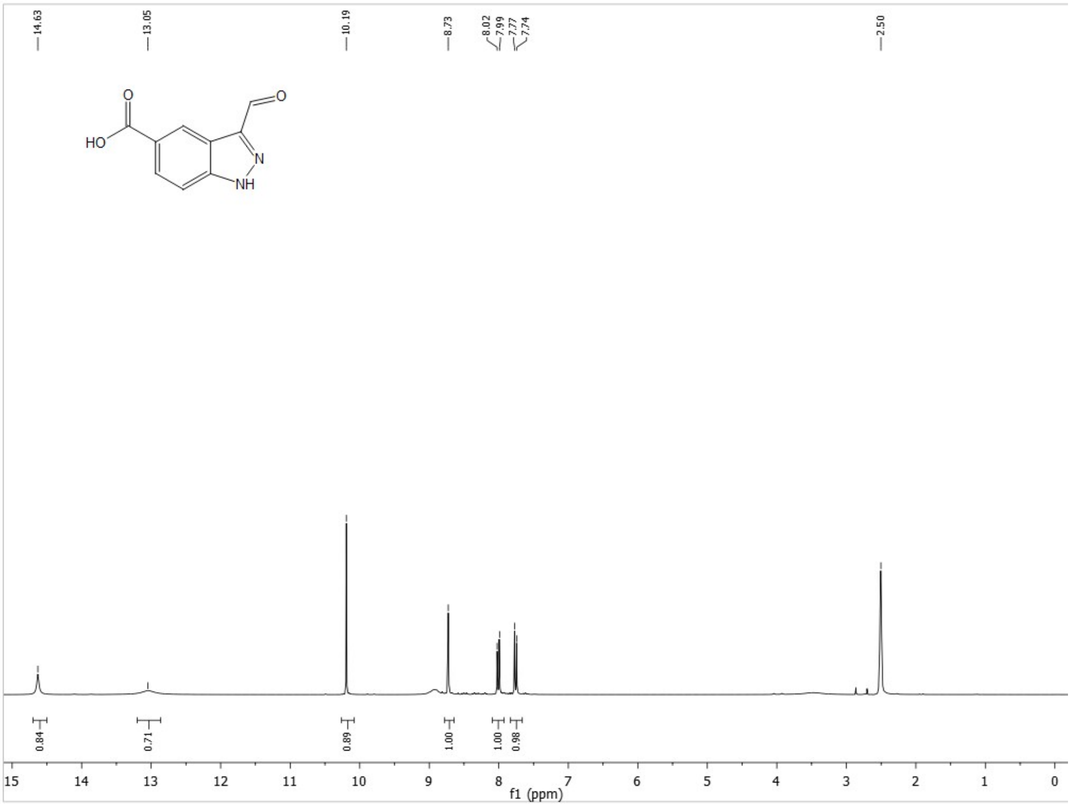

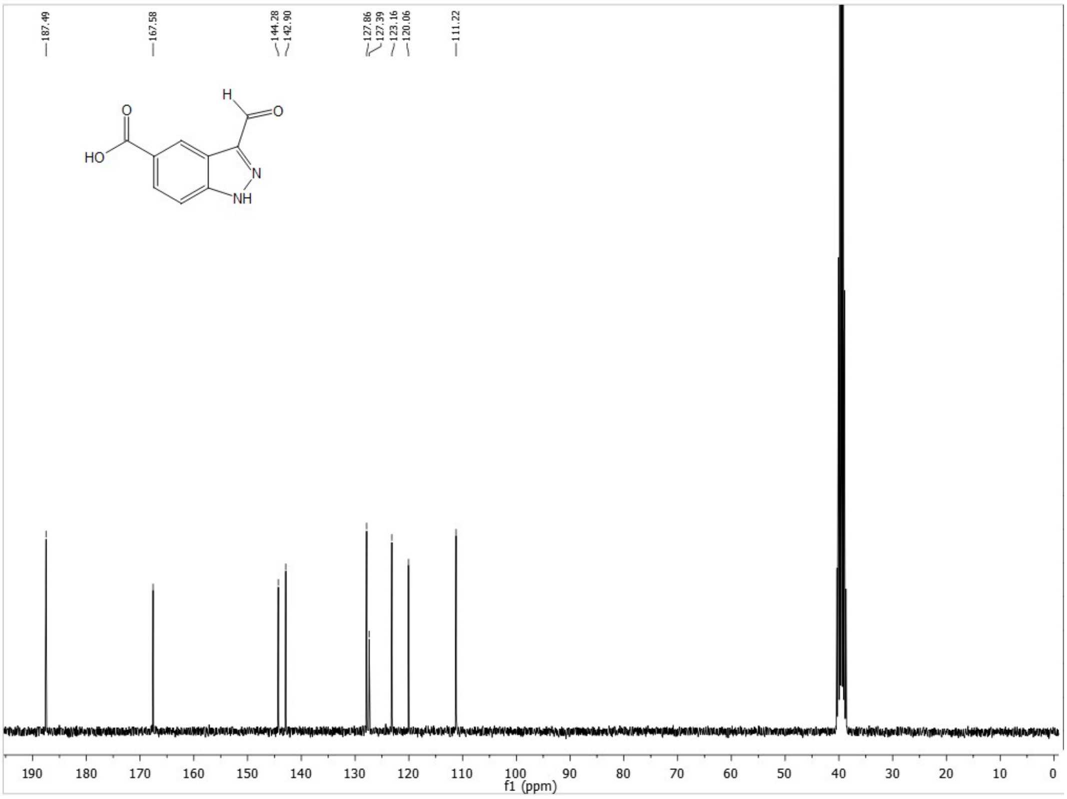

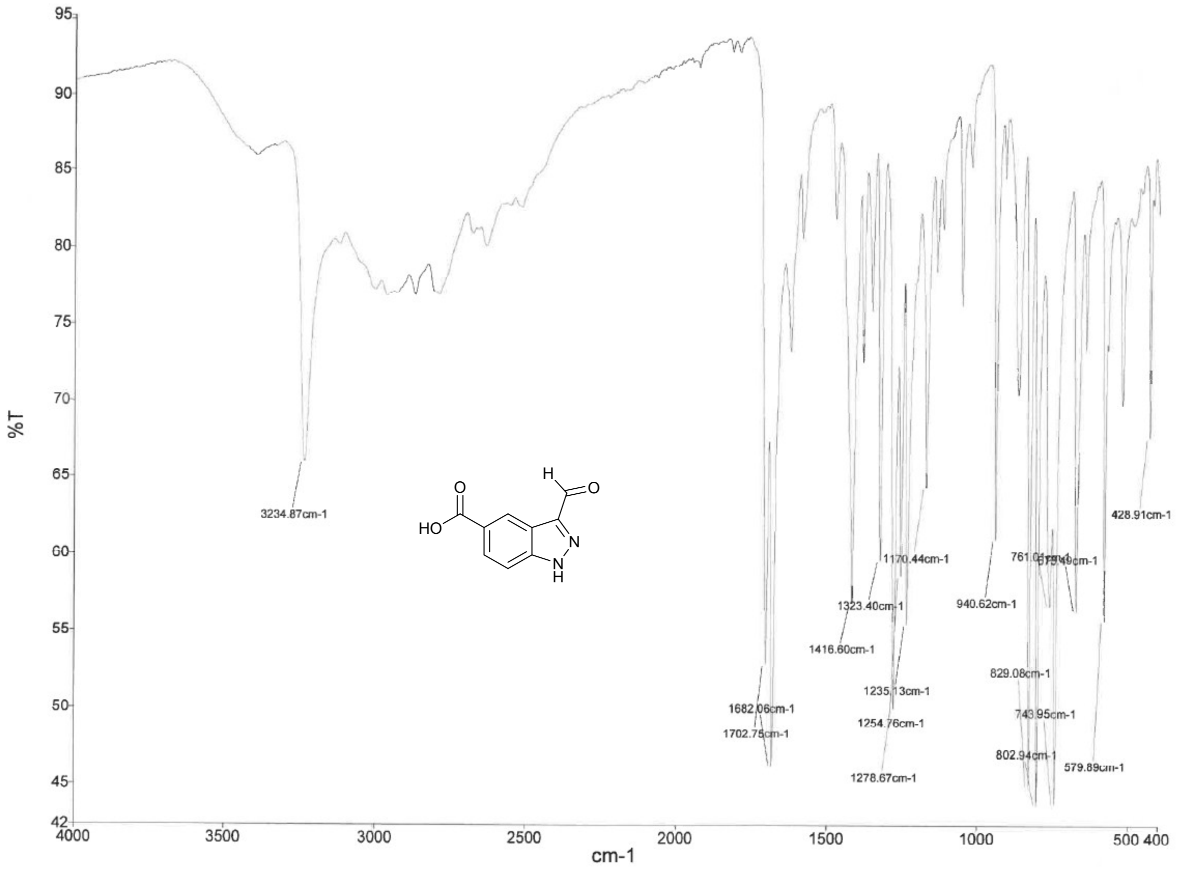
%T
cm-1
3234.87cm-1
1682.06cm-1
1702.75cm-1
1416.60cm-1
1323.40cm-1
1278.67cm-1
1254.76cm-1
1235.13cm-1
1170.44cm-1
940.62cm-1
829.08cm-1
802.94cm-1
743.95cm-1
579.89cm-1
428.91cm-1
HO
O
H
O
N
H

5-NHBoc -1*H*-indazole-3-carboxaldehyde (22b)

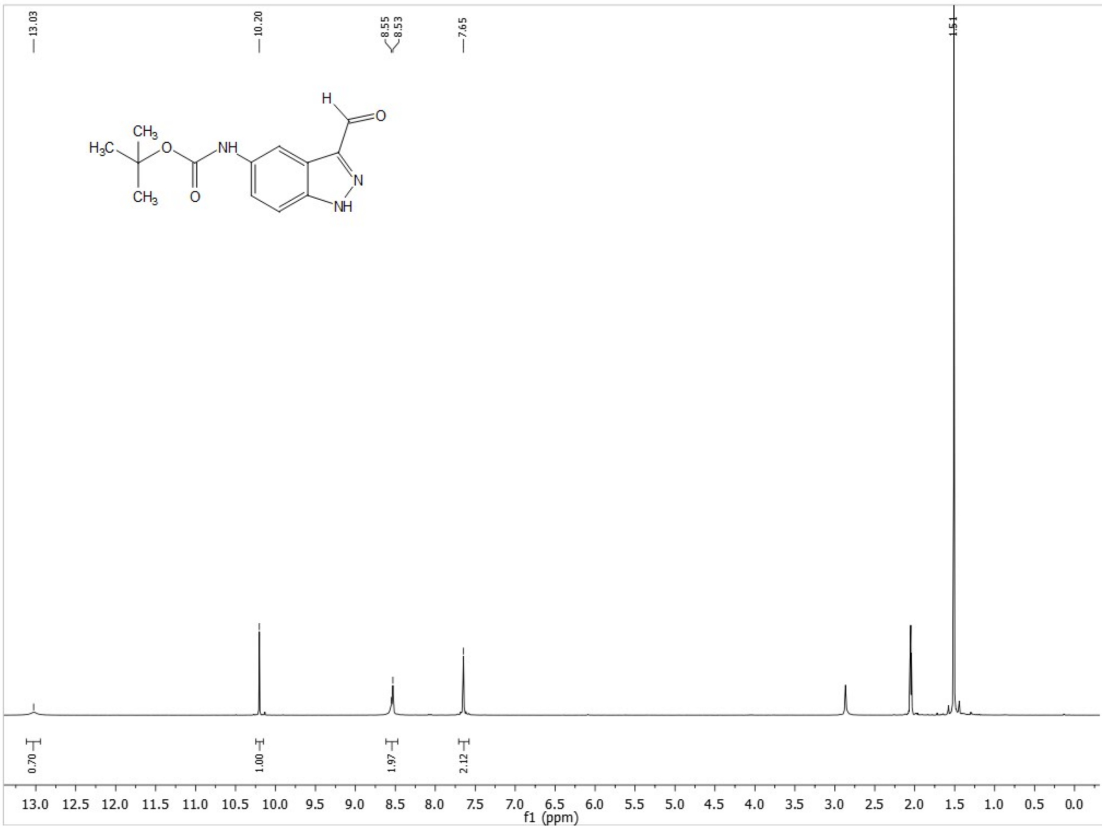

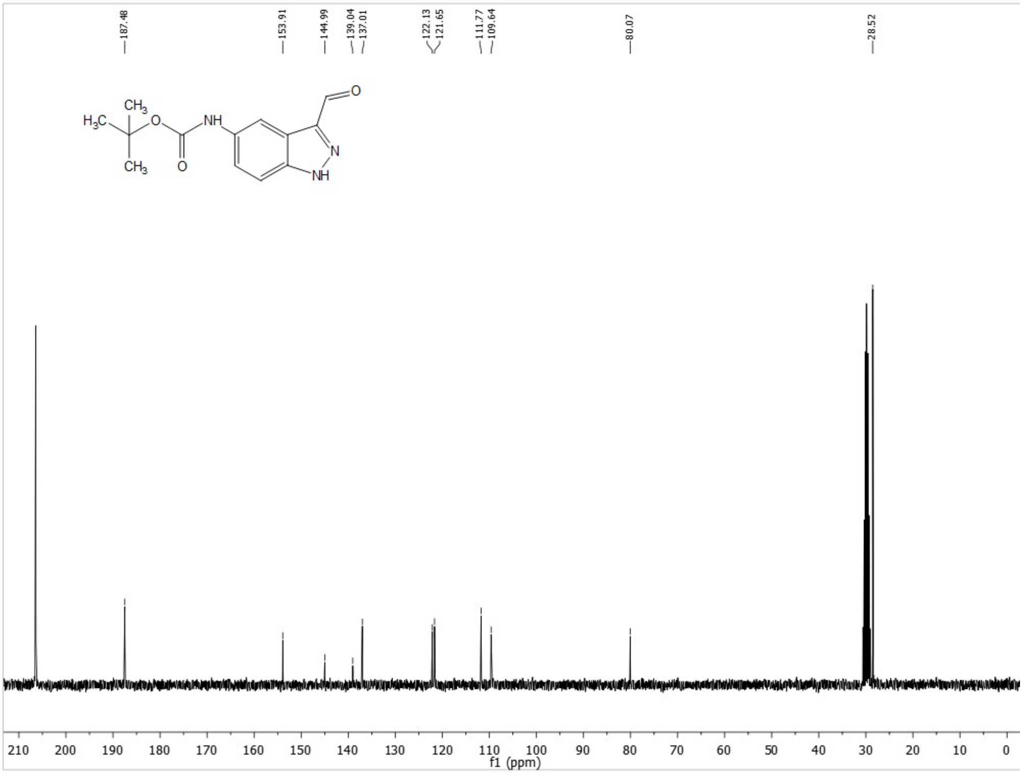

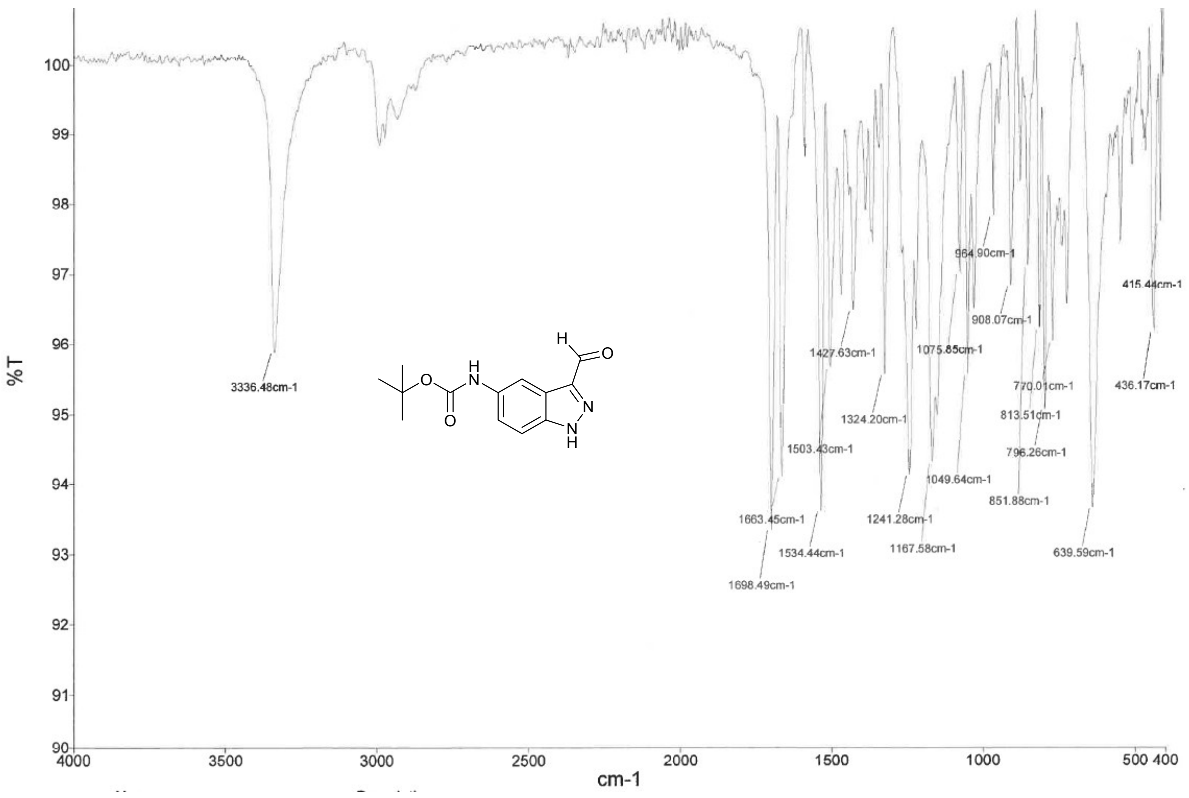

%T
cm-1
100
99
98
97
96
95
94
93
92
91
90
4000
3500
3000
2500
2000
1500
1000
500 400
3336.48cm-1
1698.49cm-1
1663.45cm-1
1534.44cm-1
1503.43cm-1
1427.63cm-1
1324.20cm-1
1241.28cm-1
1167.58cm-1
1075.85cm-1
1049.64cm-1
964.90cm-1
908.07cm-1
851.88cm-1
813.51cm-1
796.26cm-1
770.01cm-1
639.59cm-1
436.17cm-1
415.44cm-1

5-piperidyl-1*H*-indazole-3-carboxaldehyde (23b)

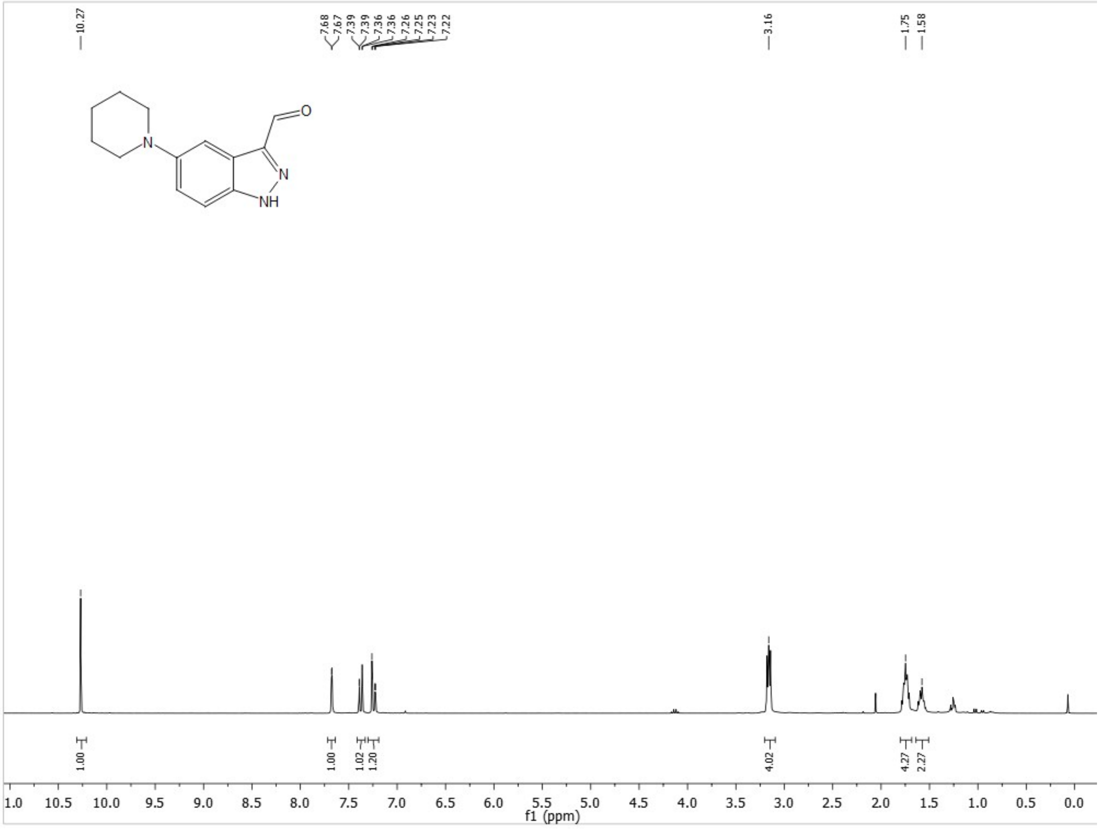

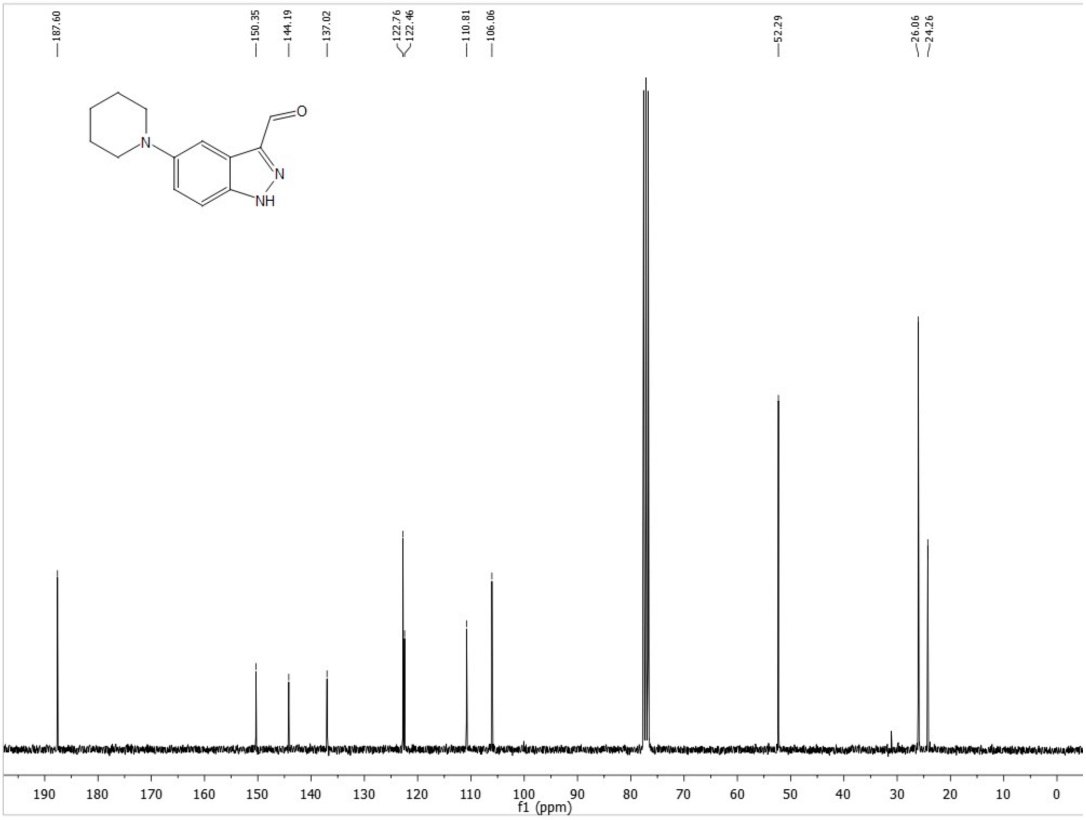

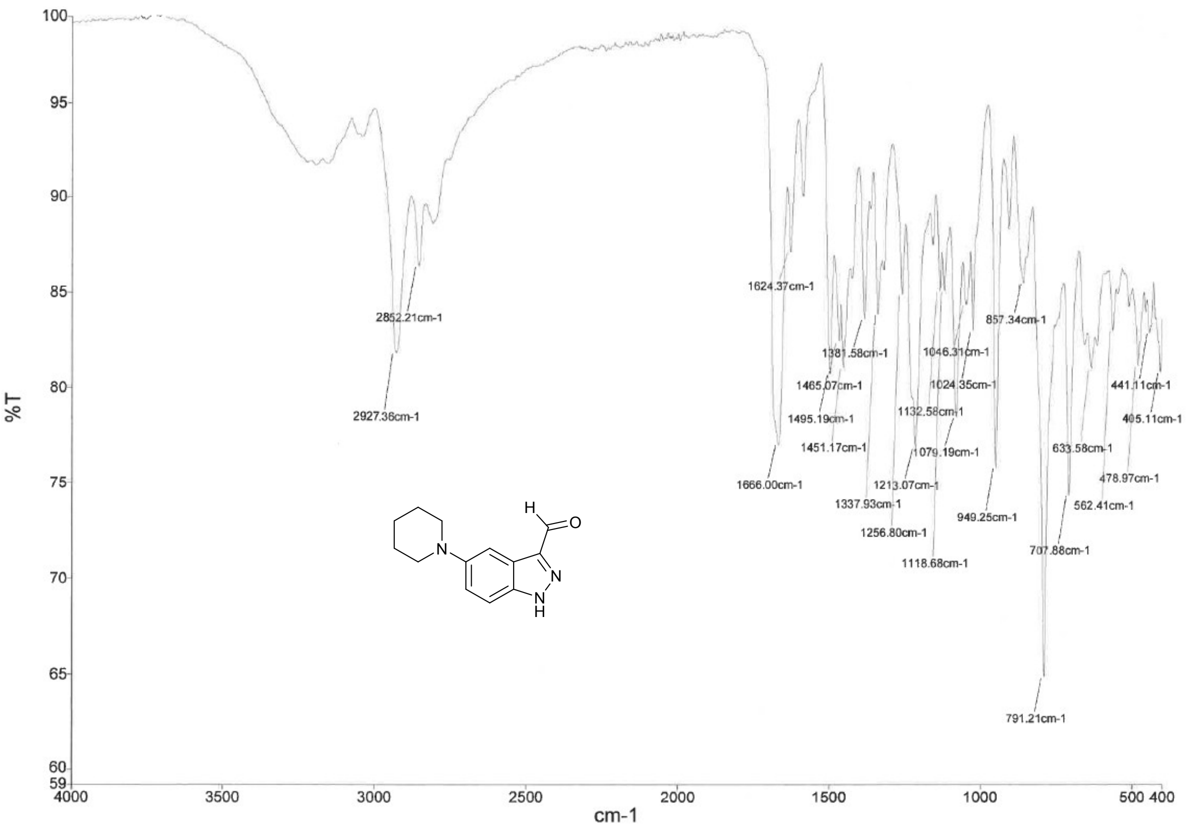

2852.21cm-1
2927.38cm-1
1624.37cm-1
1666.00cm-1
1381.58cm-1
1465.07cm-1
1495.19cm-1
1451.17cm-1
1337.93cm-1
1256.80cm-1
1213.07cm-1
1132.58cm-1
1118.68cm-1
1079.19cm-1
1046.31cm-1
1024.35cm-1
949.25cm-1
857.34cm-1
791.21cm-1
707.88cm-1
633.58cm-1
562.41cm-1
478.97cm-1
441.11cm-1
405.11cm-1
%T
cm-1
H
O
N
N
H

1*H*-indazole-3,5-dicarboxaldehyde (24b)

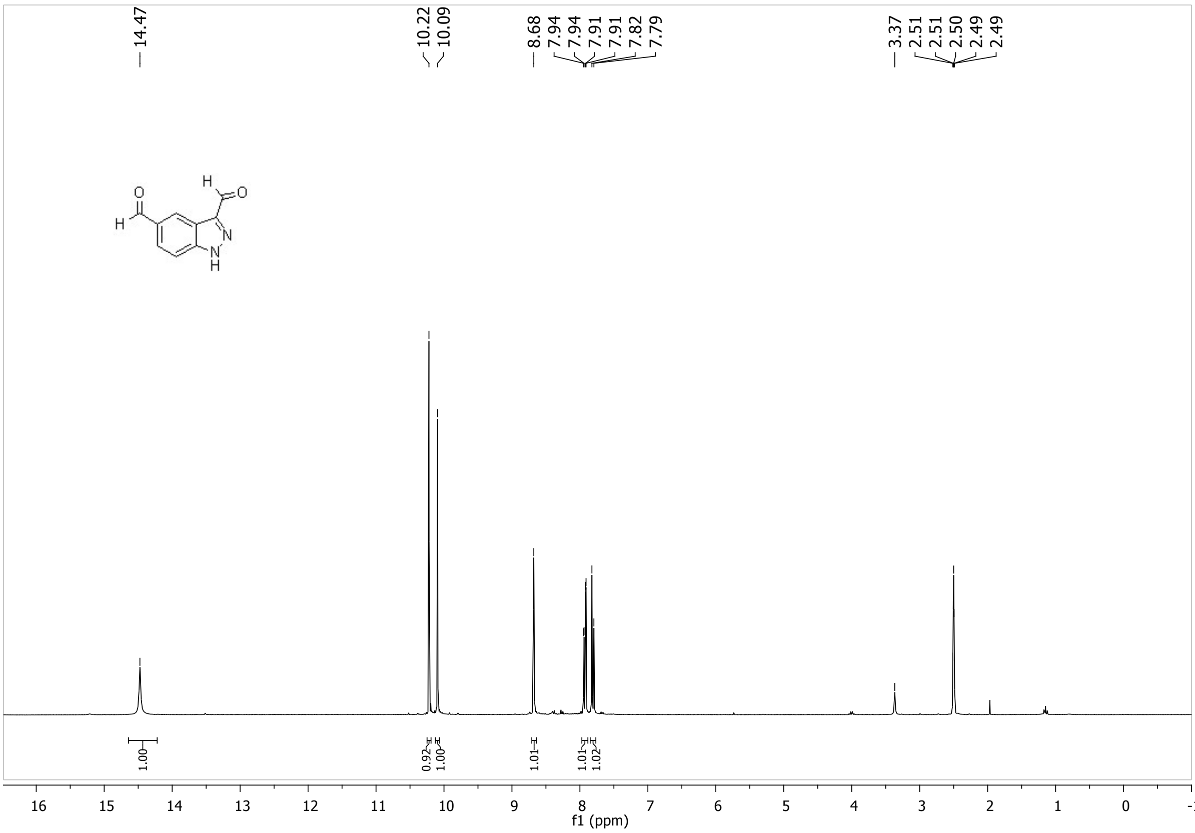

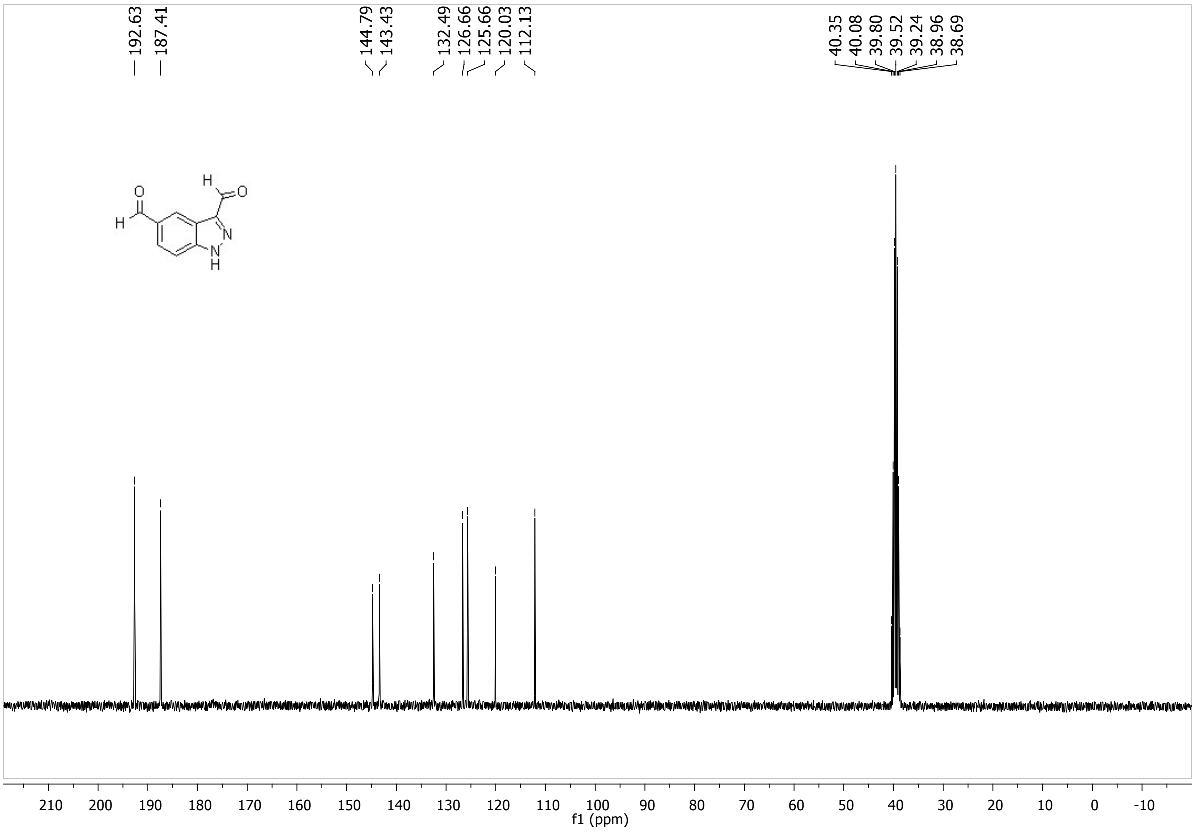

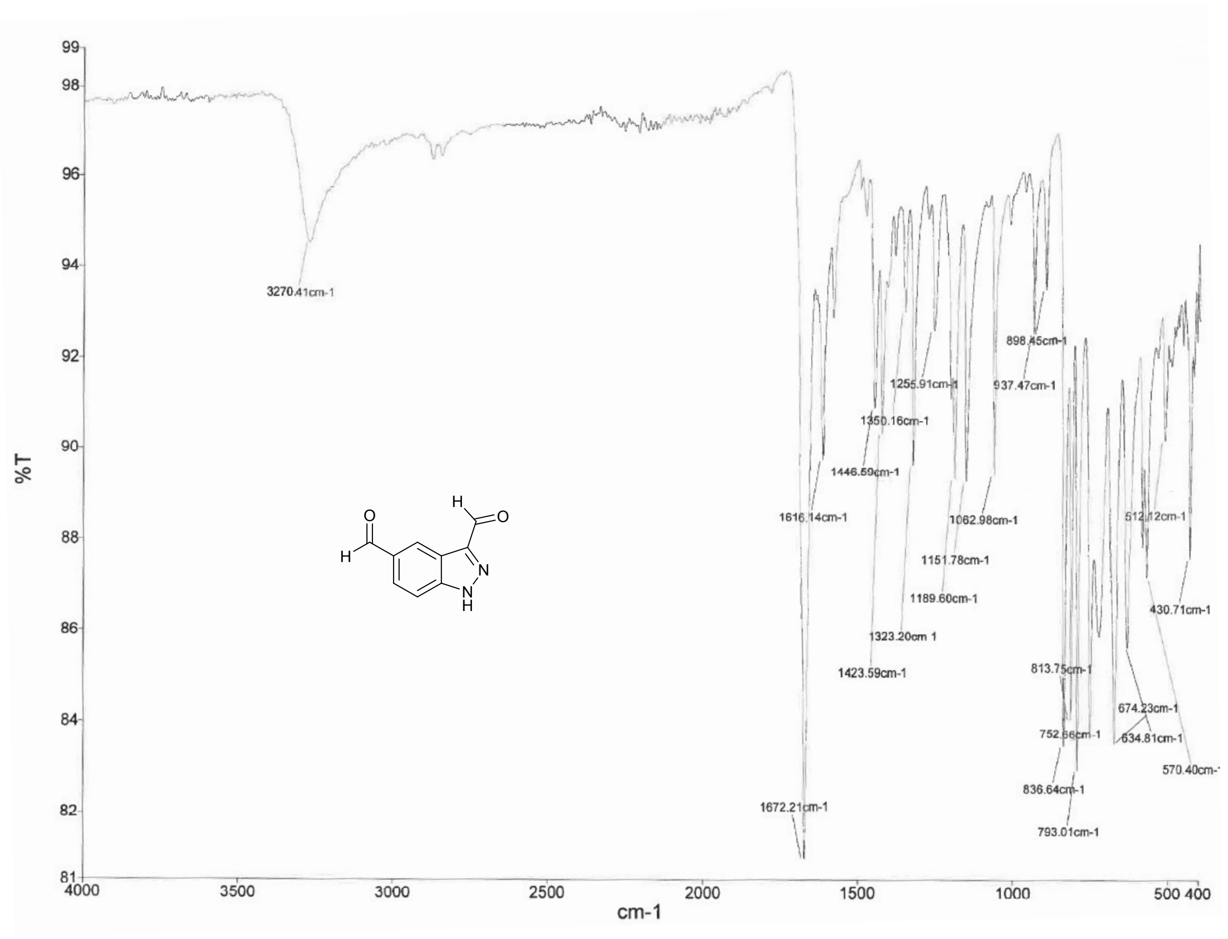

%T
cm-1
99
98
96
94
92
90
88
86
84
82
81
4000
3500
3000
2500
2000
1500
1000
500 400
3270.41cm-1
1672.21cm-1
1616.14cm-1
1446.59cm-1
1423.59cm-1
1350.16cm-1
1323.20cm 1
1255.91cm-1
1189.60cm-1
1151.78cm-1
1062.98cm-1
937.47cm-1
898.45cm-1
836.64cm-1
813.75cm-1
793.01cm-1
752.66cm-1
674.23cm-1
634.81cm-1
570.40cm-
512.12cm-1
430.71cm-1

5-cyano-1*H*-indazole-3-carboxaldehyde (25b)

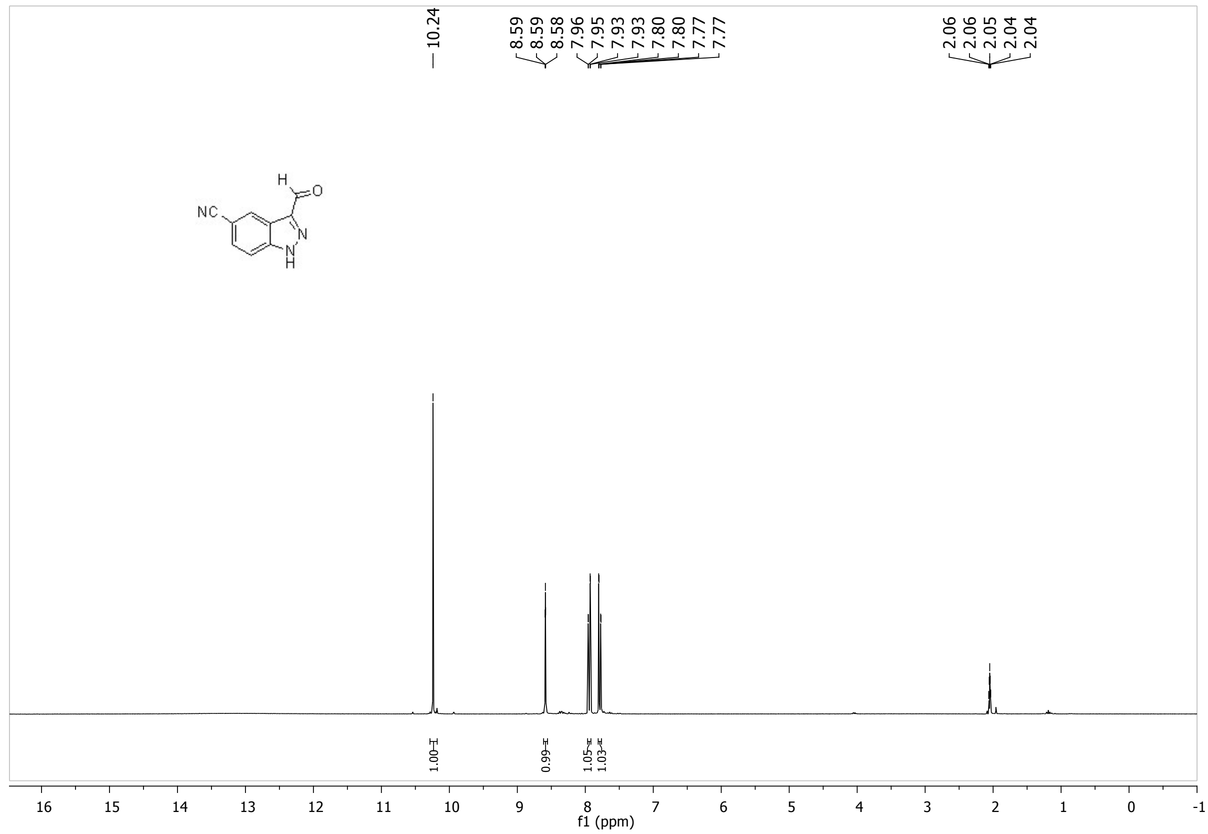

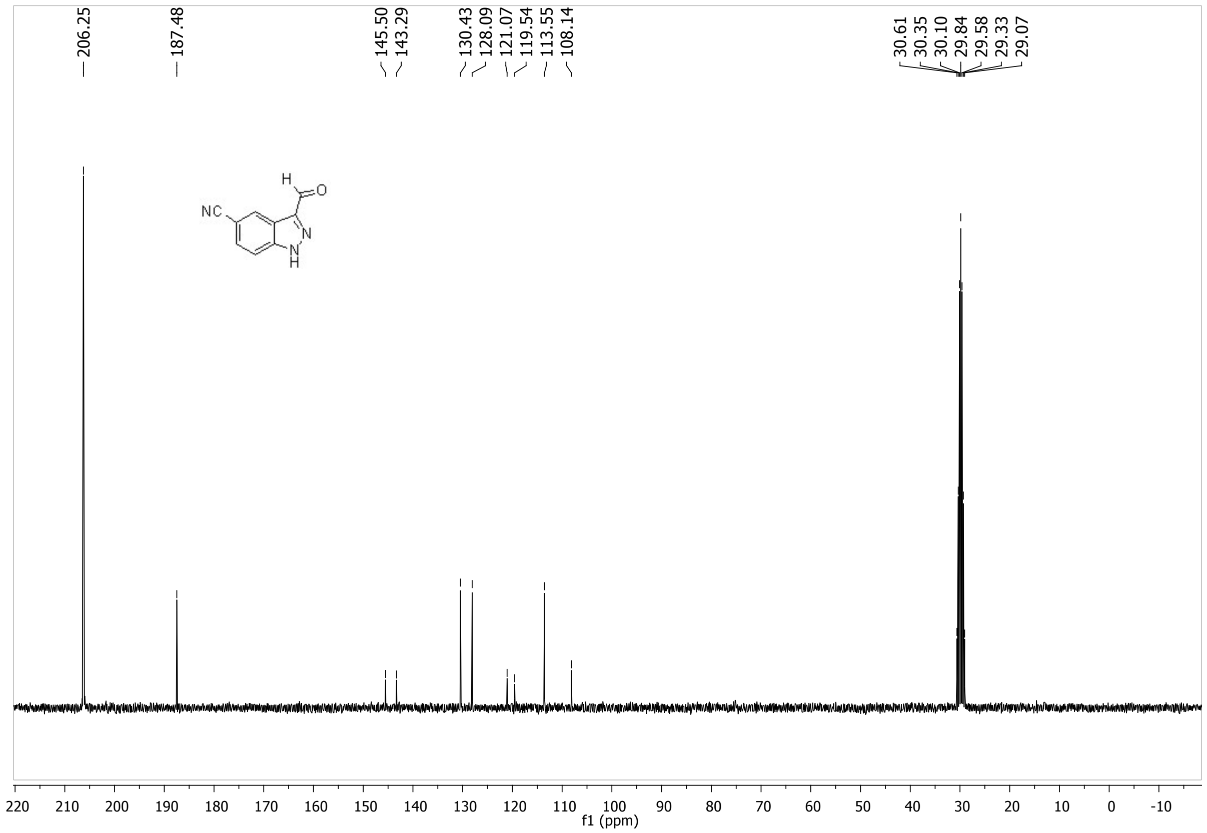

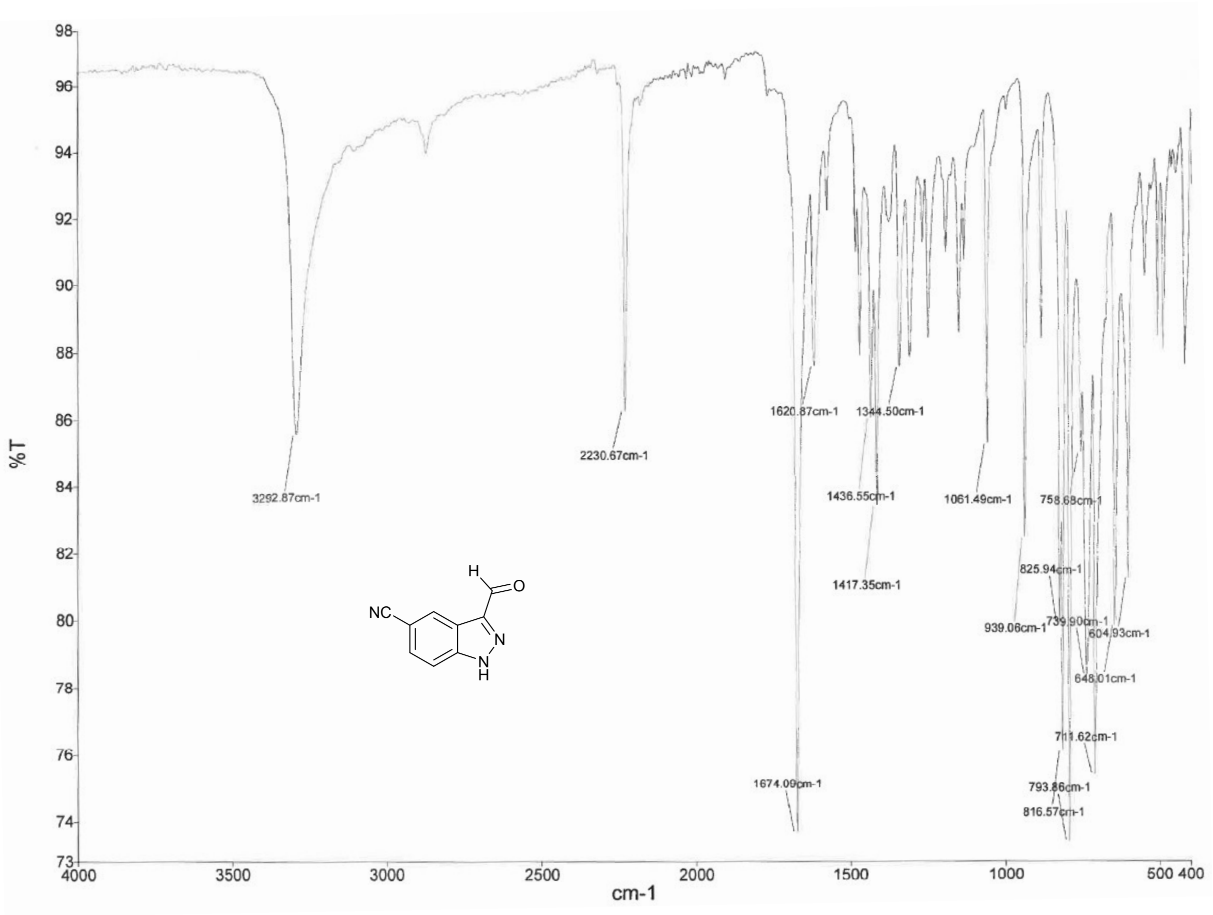
%T
cm-1
98
96
94
92
90
88
86
84
82
80
78
76
74
73
4000
3500
3000
2500
2000
1500
1000
500 400
3292.87cm-1
2230.67cm-1
1620.87cm-1
1344.50cm-1
1436.55cm-1
1417.35cm-1
1061.49cm-1
758.68cm-1
825.94cm-1
939.06cm-1
739.90cm-1
604.93cm-1
648.01cm-1
711.62cm-1
1674.09cm-1
793.86cm-1
816.57cm-1
H
O
NC
N
H

5-nitro-1*H*-indazole-3-carboxaldehyde (26b).

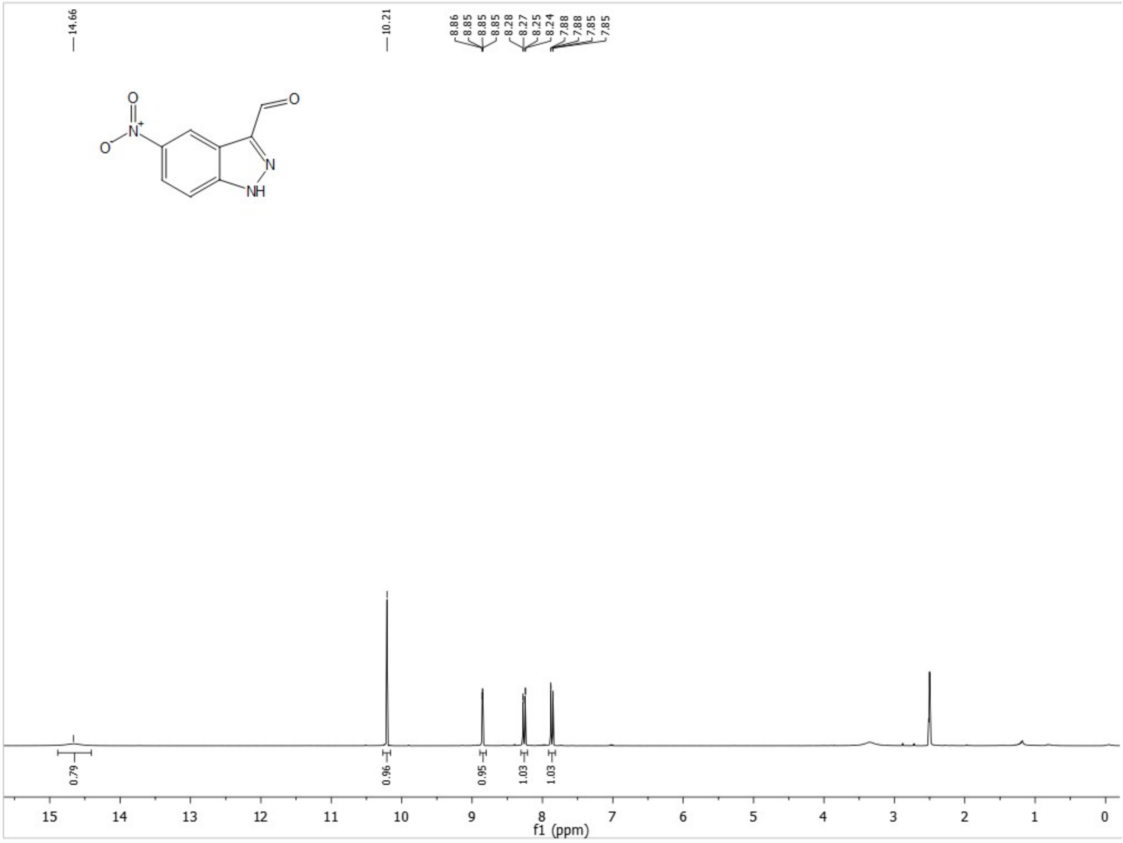

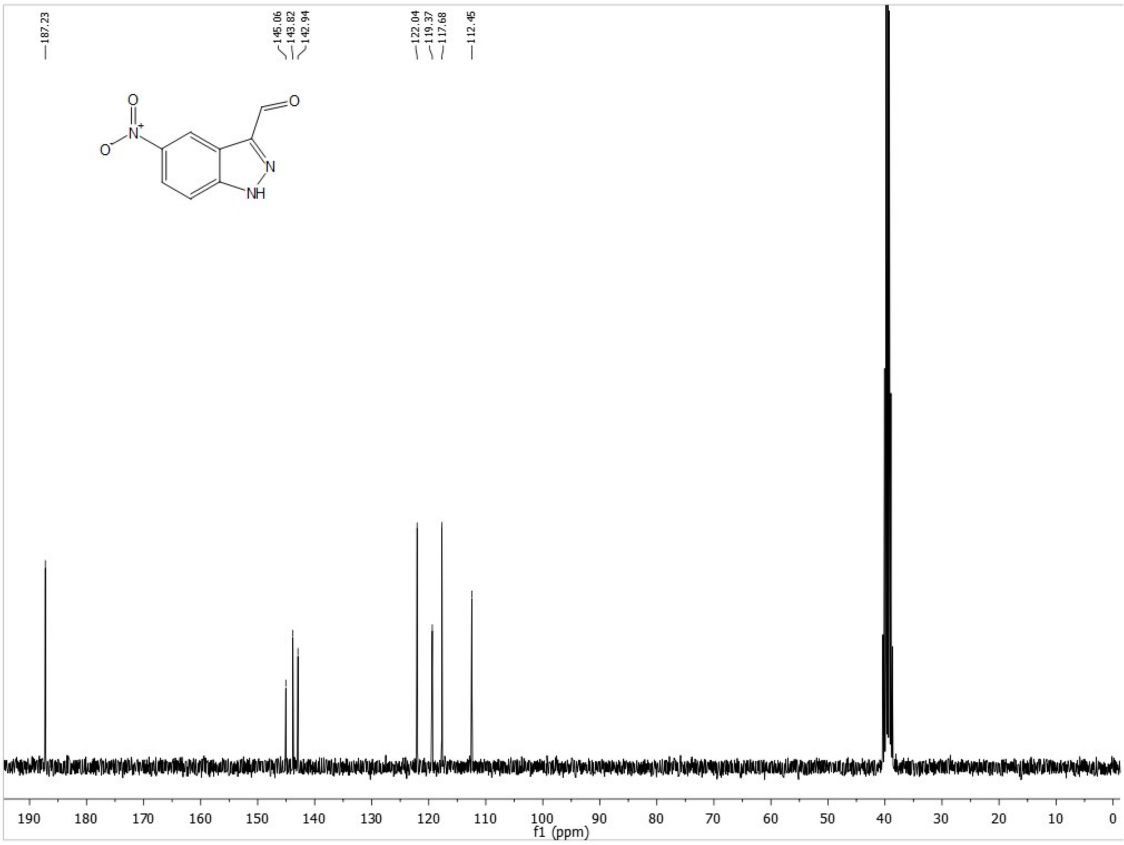

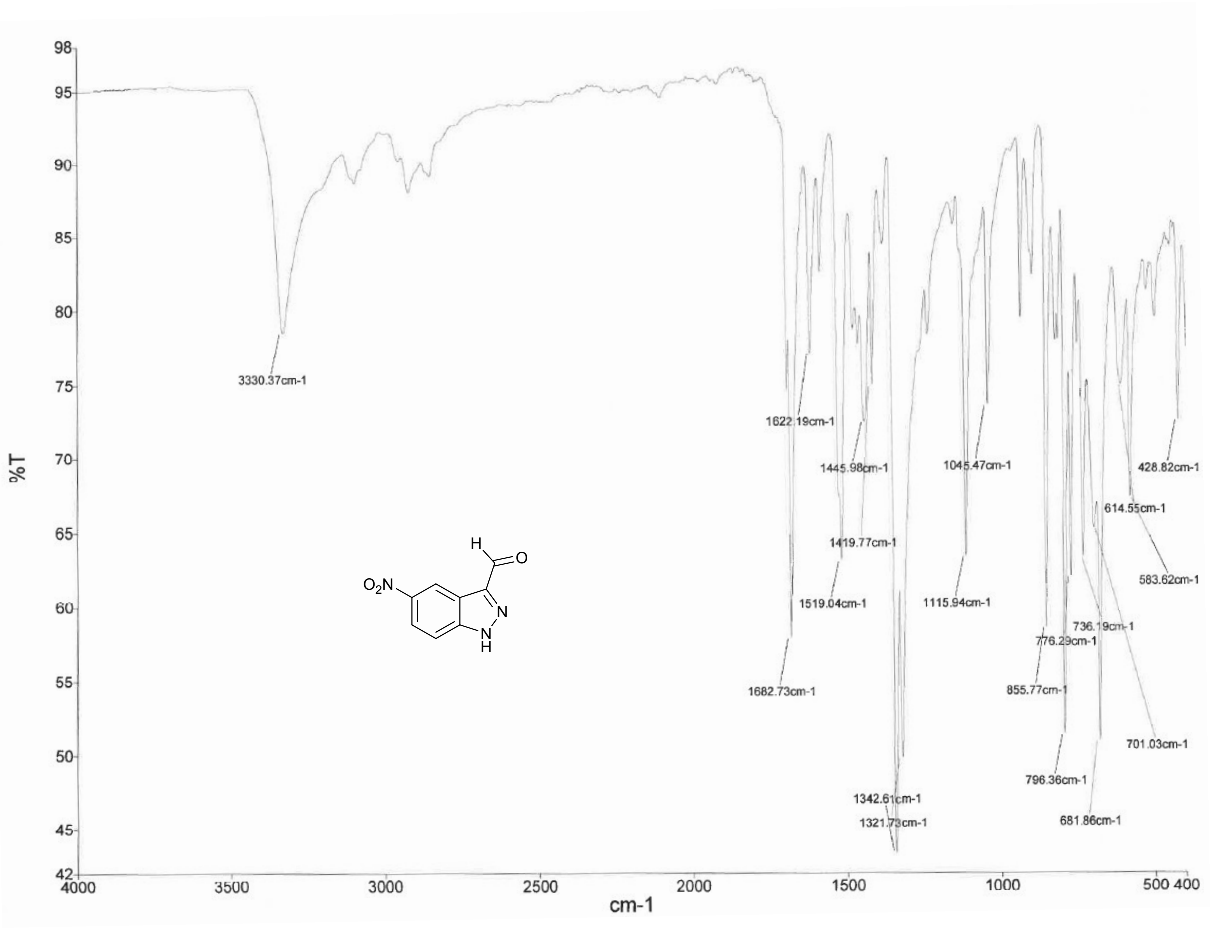
98
95
90
85
80
75
70
65
60
55
50
45
42
%T
4000
3500
3000
2500
2000
1500
1000
500 400
cm-1
3330.37cm-1
1622.19cm-1
1445.98cm-1
1419.77cm-1
1519.04cm-1
1682.73cm-1
1342.61cm-1
1321.73cm-1
1045.47cm-1
1115.94cm-1
855.77cm-1
776.29cm-1
736.19cm-1
796.36cm-1
681.86cm-1
701.03cm-1
614.55cm-1
583.62cm-1
428.82cm-1
H
O
O_2N
N
N
H

6-nitro-1*H*-indazole-3-carboxaldehyde (27b)

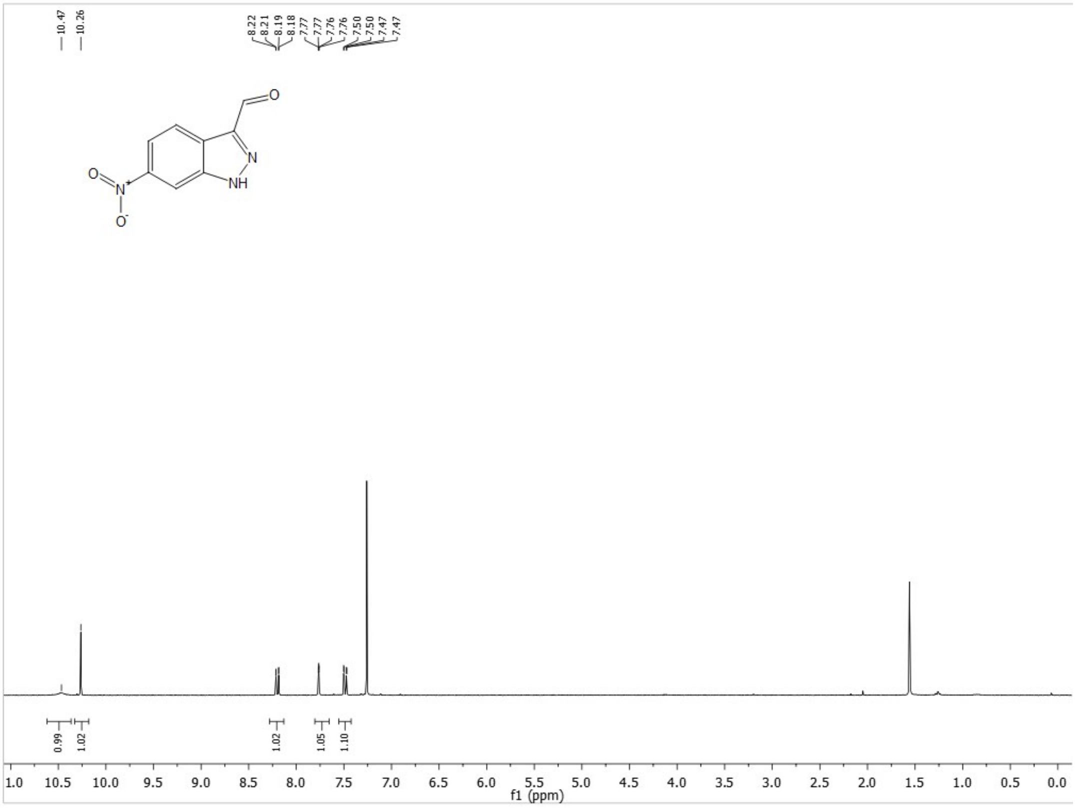

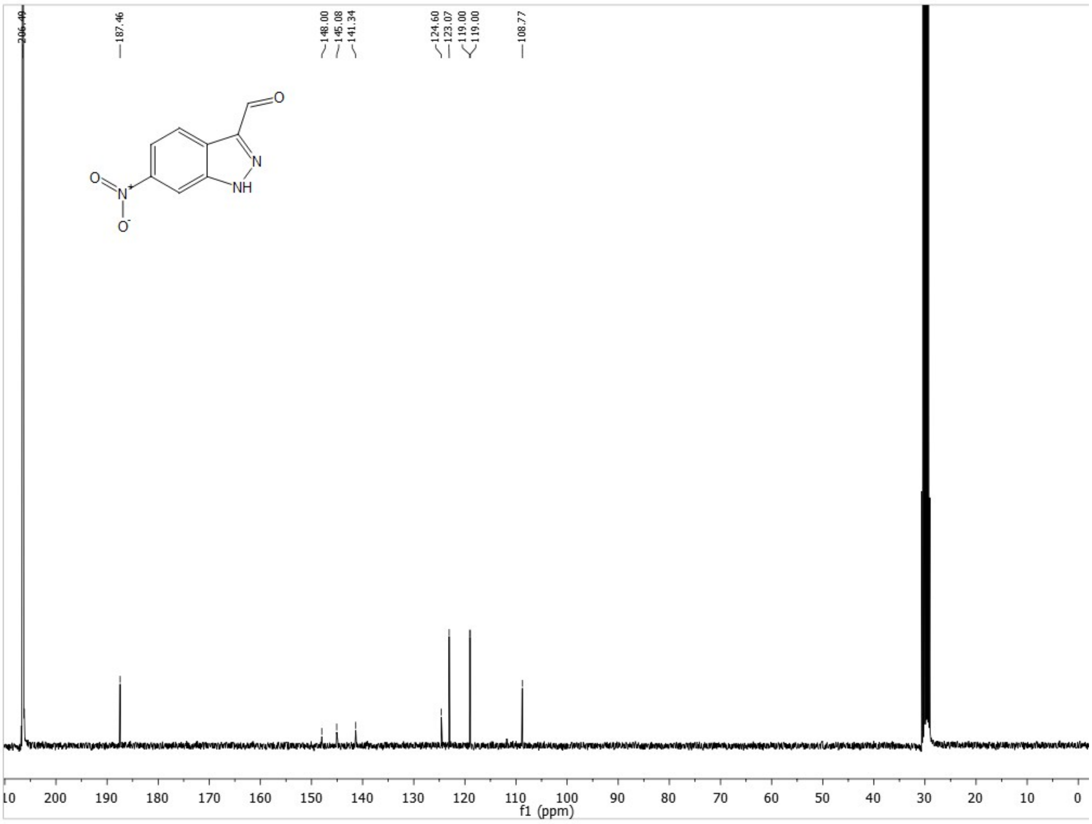

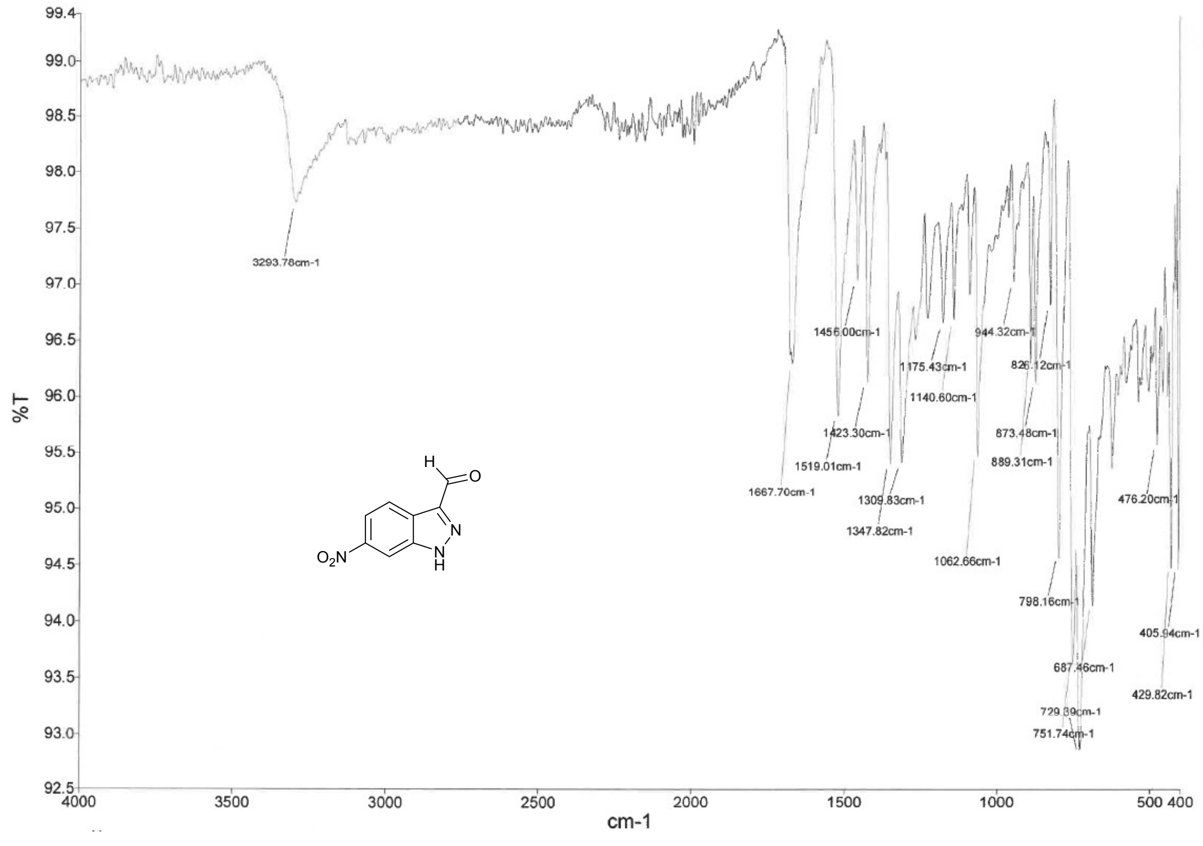
%T
cm-1
3293.78cm-1
1667.70cm-1
1519.01cm-1
1456.00cm-1
1423.30cm-1
1347.82cm-1
1309.83cm-1
1175.43cm-1
1140.60cm-1
1062.66cm-1
944.32cm-1
889.31cm-1
873.48cm-1
826.12cm-1
798.16cm-1
751.74cm-1
729.89cm-1
687.46cm-1
476.20cm-1
429.82cm-1
405.94cm-1
